# Development of a Joint Hydrogen and Syngas Combustion Mechanism Based on an Optimization Approach

**DOI:** 10.1002/kin.21006

**Published:** 2016-05-20

**Authors:** Tamás Varga, Carsten Olm, Tibor Nagy, István Gy. Zsély, Éva Valkó, Róbert Pálvölgyi, Henry. J. Curran, Tamás Turányi

**Affiliations:** ^1^ Institute of ChemistryEötvös University (ELTE) 1117BudapestHungary; ^2^ MTA‐ELTE Research Group on Complex Chemical Systems 1117BudapestHungary; ^3^ Institute of Materials and Environmental ChemistryMTA Research Centre for Natural Sciences 1117 BudapestBudapestHungary; ^4^ Combustion Chemistry CentreNational University of Ireland University RdGalway (NUIG)Ireland

## Abstract

A comprehensive and hierarchical optimization of a joint hydrogen and syngas combustion mechanism has been carried out. The Kéromnès et al. (*Combust Flame*, 2013, 160, 995–1011) mechanism for syngas combustion was updated with our recently optimized hydrogen combustion mechanism (Varga et al., *Proc Combust Inst*, 2015, 35, 589–596) and optimized using a comprehensive set of direct and indirect experimental data relevant to hydrogen and syngas combustion. The collection of experimental data consisted of ignition measurements in shock tubes and rapid compression machines, burning velocity measurements, and species profiles measured using shock tubes, flow reactors, and jet‐stirred reactors. The experimental conditions covered wide ranges of temperatures (800–2500 K), pressures (0.5–50 bar), equivalence ratios (*ϕ* = 0.3–5.0), and C/H ratios (0–3). In total, 48 Arrhenius parameters and 5 third‐body collision efficiency parameters of 18 elementary reactions were optimized using these experimental data. A large number of directly measured rate coefficient values belonging to 15 of the reaction steps were also utilized. The optimization has resulted in a H_2_/CO combustion mechanism, which is applicable to a wide range of conditions. Moreover, new recommended rate parameters with their covariance matrix and temperature‐dependent uncertainty ranges of the optimized rate coefficients are provided. The optimized mechanism was compared to 19 recent hydrogen and syngas combustion mechanisms and is shown to provide the best reproduction of the experimental data.

## INTRODUCTION

In recent years, there has been an increased interest in studying the combustion of hydrogen, and fuel mixtures consisting of carbon monoxide and hydrogen, referred to as syngas or “wet CO,” potentially including additional species such as CO_2_ and/or H_2_O. The development of clean and efficient combustion technologies for these fuels requires an in‐depth knowledge of the chemical processes that occur during combustion. The high‐temperature combustion of hydrocarbons and oxygenates is also governed by the chemistry of hydrogen and syngas combustion. Therefore, accurate knowledge of these processes is essential in the development of combustion mechanisms for any larger fuel molecules.

Several combustion mechanisms have been published for hydrogen and syngas in the past years, as discussed in the reviews of Davis et al. 1, Sun et al. 2, Li et al. 3, Ó Conaire et al. 4, Konnov et al. 5, Alekseev et al. 6, Hong et al. 7, Burke et al. 8, and Kéromnès et al. 9. Most of these recent mechanisms were assembled based on directly measured or theoretically calculated rate coefficients, but some rate parameters were commonly modified to improve agreement with measured ignition delay times, burning velocities or concentration profile measurements. These types of experimental data are usually referred to as indirect measurements, since such experimental results are interpretable only by simulations based on a detailed chemical kinetic model.

Owing to advances in computational performance and simulation techniques in the field of combustion, the optimization of mechanisms also became a viable method for developing better combustion models. Mechanism optimization is a process which involves a systematic search of parameter values of the combustion model within their physically realistic domain to achieve the best possible reproduction of a selected set of experimental results. In most studies, rate parameters are modified in this way. In principle, thermodynamic and transport parameters can also be included in an optimization task, but changing the values of these parameters within their uncertainty ranges usually has a much smaller effect on simulation results than the rate parameters. This is especially true for the H_2_/CO combustion system 10, 11 and in general for systems that involve only small molecules, whose physical parameters are known with little uncertainty.

The use of parameter optimization techniques to improve detailed combustion models was first proposed by Frenklach and Miller 12, 13, 14, and an algorithm was described in the article of Frenklach et al. 15. GRI‐Mech 3.0 16 was developed for natural gas combustion using this methodology, and it is still one of the most widely used mechanisms today. Frenklach et al. further developed the mechanism optimization approach toward data collaboration 17, 18, 19, 20, 21, providing an implementation of the method on the PrIMe website 22, and recommended the usage of the PrIMe data format 23. Another series of mechanism optimization papers was published by Wang et al. 1, 24, 25 and Sheen and Wang 26, 27. The method of uncertainty quantification and minimization using polynomial chaos expansions proposed by Sheen and Wang 27 also provides a way to calculate the covariance matrix of the fitted parameters. These methods were summarized in a recent review article 28.

Frenklach et al. and Wang et al. selected a small number of optimization targets based on representative indirect measurement data and identified the most influential rate parameters (called “active parameters”) at these conditions using local sensitivity analysis. The selected active parameters included frequency (*A*)‐factors of the rate expressions, third‐body collision efficiency parameters, and enthalpies of formation of certain species. The authors created polynomial surrogate models (“response surfaces”) for each optimization target, each of which expressed the simulation result at the conditions of an optimization target as a function of the values of the active parameters.

It was found by both Frenklach et al. and Wang et al. that after optimization many of the *A*‐factors obtained were at the edges of their assigned uncertainty intervals. To address this issue, the objective function was modified in their more recent studies so that the deviation of the *A*‐factors from their initial values is penalized 21, 23, 26, 27. As the initial values of the *A*‐factors were based on direct measurements or other recommendations, adherence to these values could be improved with this penalization and the optimized values did not approach the edges of the uncertainty intervals.

Using the methods of Frenklach et al. and Wang et al., several hydrogen and syngas combustion mechanisms were optimized. Davis et al. 1 produced an optimized syngas combustion mechanism, based on both hydrogen and syngas combustion optimization targets. They considered 36 optimization targets, including measured laminar burning velocities, concentration maxima in flat flames, flow reactor measurements, and ignition delay measurements in shock tubes. The original mechanism contained 14 species and 30 reaction steps. The authors optimized 22 of the *A*‐factors and also 6 of the third‐body efficiencies.

You et al. 23 used the data collaboration method for the optimization of a hydrogen combustion mechanism. They used 8 ignition delay times measured in shock tubes and 4 flow reactor measurements as optimization targets and optimized the *A*‐factors of all of the 21 reaction steps of their initial mechanism. Li et al. 29 created an optimized H_2_/CO combustion model, based on the optimized hydrogen combustion model of You et al. 23, also using the data collaboration methodology. They used 39 optimization targets, including 23 laminar burning velocity measurements, 7 flow reactor measurements, and 9 ignition delay measurements. Eighteen *A*‐factors were optimized within their assigned uncertainty ranges, and the resulting model could describe 35 of the optimization targets within their respective assigned uncertainty ranges. Note that results pertaining to the performance of these mechanisms are reported in Table III of this article.

Cai and Pitsch 30 suggested the optimization of rate rules for the development of combustion models for larger hydrocarbons. By optimizing rate rules instead of rate parameters of selected reactions, the dimensionality of the task can be reduced and the consistency of rate coefficients of kinetically similar reactions can be guaranteed. However, this technique cannot be applied to a syngas combustion mechanism, as the chemistry of syngas combustion does not contain analogous reactions. Mechanism optimization was also used as a correction step after mechanism reduction 31, 32.

Nagy et al. 33 recently published a review on the most important elementary reaction steps in the hydrogen and syngas combustion system. Rate parameters suggested in the literature for 22 elementary reactions were systematically evaluated, and temperature‐dependent uncertainties of the rate coefficients and joint uncertainty domains of the Arrhenius parameters were determined. These domains were stored efficiently in the form of the covariance matrix of Arrhenius parameters. The evaluated uncertainty domains are used in the present work as constraints in the parameter space in the global optimization of the rate parameters.

The authors of the present article also suggested an optimization methodology 34, 35, which is applied here. This method is different in many respects from the previously reported optimization methods. Identification of the active parameters is also carried out using local sensitivity analysis, which is a widespread tool for the analysis of combustion models 36. The experimental data are stored using the ReSpecTh kinetics data format 37, which is an extension of the PrIMe data format 22. Response surfaces are also used to improve the computational efficiency of the method. The main differences are that (i) a large number of indirect experimental data are used as optimization targets instead of a small selected set, (ii) all Arrhenius parameters (*A*, *n*, *E*) of the important reactions are optimized (not only the *A*‐factors), (iii) the response surfaces are employed to replace flame calculations only, (iv) new algorithms are used for generating response surfaces and for the global parameter estimation, and (v) temperature‐dependent uncertainties are estimated for the optimized rate coefficients. Also, instead of penalizing the deviation of the optimized rate coefficients from the recommended values, direct rate coefficient measurements are included as optimization targets. In this way, a comprehensive optimization can be carried out, and since almost all available related experimental data are utilized, the rate parameters obtained can be considered to be the best representation of the kinetic information that can be extracted from the utilized experimental results.

The methodology described above has been used for interpretation of experimental data 34, 38, 39. Moreover, Varga et al. 35 used this method for the optimization of a hydrogen combustion mechanism and it was also employed by Olm et al. in creating an optimized ethanol combustion mechanism 40.

The optimization of a complete combustion mechanism using a large amount of experimental data from different sources required a further extension of the methodology defined in 34, which could not be described in detail in the paper 35 due to space limitations. Therefore, this is the first article in which the technical details of this extension to our mechanism optimization method are described.

This work describes the development of an optimized, joint hydrogen and syngas combustion mechanism, which was carried out in the following steps: the collection of indirect experimental data (second section), assembling the initial mechanism (third section), selection of rate parameters to be optimized (fourth section), selection of methods for parameter optimization and calculation of parameter uncertainties (fifth section), calculation of response surfaces (sixth section), and the definition of the hierarchical optimization strategy (seventh section). Using this range of methods, an optimized mechanism was obtained that was tested against a large range of experimental data and compared to other recent hydrogen and syngas reaction mechanisms (eighth section).

## COLLECTION OF INDIRECT EXPERIMENTAL DATA

A large set of indirect experimental data relevant to both hydrogen and syngas combustion was collected and used. For hydrogen combustion, all data that were used in the modeling study of Olm et al. 41 and during the development of our optimized hydrogen combustion mechanism 35 were also considered here. New experimental data were added from Hashemi et al. 42. Altogether 770 ignition delay measurements from shock tubes (53 data sets), 229 ignition delay measurements from rapid compression machines (RCMs) (20 data sets), 443 concentration measurements from flow reactors (17 data sets), 152 concentration measurements from jet‐stirred reactors (JSRs) (9 data sets), and 631 laminar burning velocity measurements (73 data sets) relevant to hydrogen combustion were used in this study. A data set contains those data points that were consecutively measured using the same apparatus at similar conditions except for one experimental condition that was systematically varied.

For syngas combustion, a large set of indirect experimental data collected by Olm et al. 43 was used. While in many practical applications, syngas can contain CH_4_ and other hydrocarbons, in the present work only fuel mixtures of H_2_ and CO are considered, as well as pure CO and pure H_2_ diluted with CO_2_. In total, 732 ignition delay measurements in shock tubes (62 data sets), 492 ignition delay measurements from rapid compression machines (47 data sets), 1104 concentration measurements from flow reactors (58 data sets), 90 concentration measurements from jet‐stirred reactors (3 data sets), 436 concentration measurements from shock tubes (4 data sets), and 2116 laminar burning velocity measurements (217 data sets) were used.

A detailed list of the collected data can be found in Tables S1–S13 of the Supporting Information. All of the data collected were stored in XML files according to the ReSpecTh kinetics data format specification 37, which is an extension of the PrIMe experimental XML format 22. A formal definition of the ReSpecTh kinetics data format specification and all XML file used in this work can be downloaded from the ReSpecTh website 44.

Recently, several authors reviewed the various methods for carrying out indirect combustion experiments. They assessed the typical sources of systematic errors and the level of accuracy of these measurements. Excellent reviews were written by Egolfopoulos et al. 45 and Varea et al. 46 about uncertainties in burning velocity measurements methods; by Dryer et al. 47 concerning flow reactors; by Kéromnès 48 and Sung and Curran 49 relating to rapid compression machines; and by Pang et al. 50 for facility effects in shock tubes. In this work, we took into account the suggestions of these authors at the selection and interpretation of the data.

Based on the principles described in the reviews above, a part of the collected data was excluded from the optimization targets, or other restrictions were made. Shock tube data measured below temperatures of 1000 K (behind the reflected shock wave) were excluded due to the possible influence of facility effects which cannot be accounted for using homogeneous, adiabatic simulations. At these conditions, the pressure behind the reflected shock wave cannot be considered to be constant in time 50. For most of the shock tube measurements, pressure−time profiles, which can be used to take into account this effect, were not reported. Thi et al. 51 published their experimental data together with a characterization of the pressure increase observed during their experiments, which could be used to adequately model the experiments below 1000 K, and these data were used in the present work.

It has been noted by Burke et al. 8 and in our previous paper on the optimization of a hydrogen combustion mechanism 35 that speciated flame measurements cannot be used well for optimization, since all simulation results are far more sensitive to the temperature profile than to the kinetic parameters used for the simulations. Therefore, such measurements were also not used in our present work.

Similarly, as in our optimization study on hydrogen combustion 35, we found that at the conditions of the JSR experiments the measured values showed relatively low sensitivity to the rate parameters of most reactions and were not used as optimization targets, but were used to test the final optimized mechanism. Turbulent flow reactor experiments were interpreted by shifting the simulated species profiles for small species such as H_2_ and CO to match the simulated half‐fuel depletion time with the experiments, as recommended by Dryer et al. 47. In cases where the half‐fuel depletion point was not observable in the reported results, a smaller degree of conversion was used as the matching point. Dryer et al. highlighted that this method has to be used with care, as it is possible to obtain physically unrealistic results if the shapes of the simulated and measured concentration profiles are very different. During our optimization procedure, many rate parameter sets were investigated that can result in physically unrealistic simulation results at some experimental conditions, but these parameter sets were disregarded in favor of those that produce acceptable results at all conditions.

However, such a time shift raises concerns during the uncertainty estimation of the fitted parameters. To estimate the uncertainty of the flow reactor measurements caused by the uncertainty of the time shift, we would have to know the uncertainty of the related physical/chemical processes (e.g., mixing, heat transfer, potential impurities). If measured time shift values and associated uncertainties were available, it would be possible to meaningfully propagate such uncertainties to those of the calculated rate parameters. However, in most cases the physical/chemical processes behind the time shift are not well characterized and are compounded into a single time shift effect. If the time shift was carried out without taking into account the associated uncertainties, then, from a parameter estimation point of view, such a time shift would be a free parameter. By introducing such a free parameter, our error estimation procedure would strongly underestimate the uncertainties of rate parameters, as most of the systematic discrepancies between the experimental data and the simulation results would be eliminated by shifting the concentration profiles in time.

As these types of experiments provide very valuable information on the combustion of both hydrogen and syngas, the data were used as optimization targets, but were omitted from our error estimation procedure. Also, only experimental results between fuel depletion of 90% and 10% were taken into account, since the data points relating to a little or almost complete conversion contain very little kinetic information. This also meant the complete exclusion of a small number of data sets, where no points were measured in this range of conversion.

Varga et al. 35 identified some data sets relevant to hydrogen combustion that could not be simultaneously well reproduced with the majority of the optimization targets. For syngas combustion, Olm et al. 43 also identified some measurements that could not be reproduced within 3*σ* of the experimental uncertainty by any of the mechanisms investigated, and this was indicated in the Supplementary Material of the corresponding article. In these articles 35, 43, we also demonstrated that the badly reproducible experiments are not all related to some well‐defined sets of conditions, such as high‐pressure or high equivalence ratio, and reproducible experimental measurements carried out at similar conditions are available. These few irreproducible hydrogen and syngas experiments were not used. Altogether, 12 ignition delay (11 measured in shock tubes and 1 in RCM) and 11 laminar burning velocity data sets relevant to hydrogen combustion, as well as 8 ignition delay (3 measured in shock tubes and 5 in RCMs), 8 laminar burning velocity and 17 concentration profile (15 measured using flow reactors, 1 using JSR, and 1 using shock tube) data sets relevant to syngas combustion were excluded in this way.

The indirect experiments for hydrogen and syngas combustion that were used as optimization targets consisted of 1723 ignition delays measured in shock tubes and RCMs from 156 data sets, 2311 laminar burning velocity measurements from 256 data sets, and 968 concentration values measured using shock tubes and flow reactors from 53 data sets. Furthermore, 103 concentration measurements in JSRs from 11 data sets were also included in our final comparison between mechanisms.

## THE INITIAL MECHANISM

According to our previous studies on hydrogen and syngas combustion mechanisms 41, 43, the mechanism of Kéromnès et al. 9 provides one of the best overall descriptions of the indirect experimental data related to both, hydrogen and syngas combustion. This model was also developed based on the most recent direct measurements and high‐level theoretical calculations of reaction rate coefficients. Therefore, it was used as the starting point for further mechanism development.

The hydrogen combustion submechanism was replaced with the optimized mechanism of Varga et al. 35, which was previously shown to produce the best results for the description of direct and indirect experimental data relevant to the combustion of hydrogen.

The reaction HĊO + M = Ḣ + CO + M was described in the model of Kéromnès et al. as being second order at all pressures. This elementary reaction was also handled in this way in all other published syngas combustion mechanisms. However, a deviation from the second‐order behavior at pressures of 10 bar and above has been observed experimentally by Hippler et al. 52 and Krasnoperov et al. 53. The theoretical study of Yang et al. 54 provided a high‐pressure limiting rate coefficient, a Troe fit for the falloff region, and third‐body collision efficiencies for argon and helium, relative to nitrogen. In our initial model we considered the pressure dependence for the HĊO decomposition reaction, by retaining the second‐order rate coefficient of Kéromnès et al. 9 as the low‐pressure limit for the reaction which is based on the value suggested by Li et al. 3, and using the high‐pressure limiting rate coefficient, Troe parameters and third‐body collision efficiencies of Yang et al. 54 for He and Ar, and the third‐body collision efficiencies of Kéromnès et al. for other colliders.

The third‐body collision efficiencies were handled differently in our initial mechanism compared to the Kéromnès et al. mechanism. Temperature‐dependent third‐body collision efficiencies were used by Kéromnès et al. by defining reactions that involve specific third bodies (e.g., ${\dot{H}+O}_{2}+{\mathrm{Ar}=H\dot{O}}_{2}+\mathrm{Ar},\;\;\;{\dot{H}+O}_{2}+N_{2}={H\dot{O}}_{2}+N_{2}$), and providing different parameterizations for temperature dependence of these low‐pressure limiting rate coefficients. However, this formalism produces incorrect results at high pressures, since the calculated rate coefficients are effectively multiplied at high pressures by the number of collider specific reactions. In our initial model, we converted such multiple reactions into a single one, with the closest equivalent temperature‐independent third‐body collision efficiency.

For each reaction involving third bodies, the rate coefficient was expressed with nitrogen as the reference collider having unit efficiency. Efficiencies were explicitly defined for several stable species, such as H_2_, CO, O_2_, H_2_O, CO_2_, Ar, and He. Unit relative collider efficiencies were used for most other species.

## SELECTION OF RATE PARAMETERS TO BE OPTIMIZED

A local sensitivity analysis using the initial mechanism was carried out at the conditions of the indirect experimental data. Normalized sensitivity coefficients were calculated for each of the experimental data points with respect to the *A*‐factors of each reaction, including the *A*‐factors describing the low‐pressure limiting rate coefficients. The sensitivity coefficients of the third‐body collision efficiencies were also calculated. The sensitivity coefficients were calculated using the finite difference method, and the threshold for importance was defined as 10% of the largest absolute value of the normalized sensitivity coefficients.

The rate parameters of those reactions were selected for optimization that produced high sensitivity coefficient values at several experimental conditions. Usually all three Arrhenius parameters (*A*, *n*, *E*) were optimized, unless it was reported by Nagy et al. 33 that the temperature dependence of both the rate coefficient and its uncertainty can be adequately described using fewer Arrhenius parameters (see Table I for details).

**Table I:** The Reactions Selected and the Number of Direct Measurements Used for Optimization, and the Optimized Values of the Parameters

		Direct Experiments		Optimized Parameters		
Optimized Subset of Reactions		Data Points	Data Sets	ln*A*	*n*	*E*/R
R24	CO + OH = CO_2_ + H	205	15	9.717	2.221	–694.7
R1	H + O_2_ = O + OH	745	9	36.16	–0.4859	8116
R9*a*	H + O_2_ + M = HO_2_ + M	149	10	45.41	–1.373	–
R2	O + H_2_ = H + OH	288	10	14.04	2.270	3501
R11	HO_2_ + H = OH + OH	–	–	31.69	–	86.07
R13	HO_2_ + OH = H_2_O + O_2_	67	4	27.59	0.4201	–477.4
R8*b*	H + OH + M = H_2_O + M	2	1	55.66	–2.538	60.79
R10	H + HO_2_ = H_2_ + O_2_	10	1	14.57	2.113	–817.7
R3	OH + H_2_ = H + H_2_O	181	7	16.40	1.878	1586
R18	H_2_O_2_ + H = H_2_ + HO_2_	–	–	46.03	–1.925	4743
R16	OH + OH + M = H_2_O_2_ + M	113	6	42.14	–1.178	–2150
R23	CO + O_2_ = CO_2_ + O	39	1	28.69	–	24005
R25	CO + HO_2_ = CO_2_ + OH	–	–	16.53	1.680	9139
R15	HO_2_ + HO_2_ = H_2_O_2_ + O_2_	73	4	35.01	–	7826
R26*c*	HCO + M = H + CO + M	170	8	24.62	0.9596	7368
R28	HCO + H = CO + H_2_	–	–	31.79	–	–
R4	OH + OH = O + H_2_O	173	4	11.35	2.2642	–898.2
R5	H + H + M = H_2_ + M	2	1	43.05	–1.213	308.0

^*a*^Optimized values of third‐body collision efficiency parameters (±1*σ*) of reaction Ḣ + O_2_ (+M) = HȮ_2_ (+M): *m*(H_2_) = 1.51 ± 0.25, *m*(Ar) = 0.474 ± 0.020, *m*(H_2_O) = 11.37 ± 0.95.

^*b*^Optimized value of the third‐body collision efficiency for helium (±1*σ*) of reaction Ḣ + ȮH + M = H_2_O + M: *m*(He) = 0.44 ± 0.21.

^*c*^Optimized value of the third‐body collision efficiency for helium (±1*σ*) of reaction HĊO + M = Ḣ + CO + M: *m*(He) = 0.79 ± 0.12.

For reactions with a third‐body “+M”, the optimized parameters refer to the low‐pressure limit. The order of the reactions corresponds to the order of inclusion according to the optimization strategy discussed in the seventh section. Units of the Arrhenius parameters are cm^3^ mol s K.

The list of the rate parameters chosen for optimization is given in Table I. Altogether, 48 Arrhenius parameters and 5 third‐body collision efficiencies of 18 reactions were optimized. The selected elementary reactions included all of those that were previously optimized in our recently published hydrogen combustion model 35, as well as the reactions that are relevant only to the combustion of syngas mixtures due to involving carbon containing species. Two further reactions $\dot{O}H\;+\;\dot{O}H\;=\;\ddot{O}\;+\;H_{2}O\;\left(R4\right)$ and Ḣ + Ḣ + M = H_2_ + M (R5) were also included, since the rate coefficients of these hydrogen reactions are important at the conditions of several syngas experiments, but only at a few hydrogen experiments.

The temperature dependence of the rate coefficient of reaction ${H\dot{O}}_{2}+{H\dot{O}}_{2}=H_{2}O_{2}+O_{2\;}\left(R15\right)$ can be described with the sum of two Arrhenius expressions. In the present work, the parameters of the Arrhenius expression that are relevant at higher temperatures were optimized only, while those relevant at lower temperatures were not modified.

It was found that the reactions optimized in our previous study on hydrogen combustion 35 also have a large influence on the simulation results of the syngas combustion experiments. This indicated that information on the values of the rate parameters of these elementary hydrogen combustion reactions can be inferred from syngas combustion experiments and could therefore be further optimized based on the whole (hydrogen and syngas) experimental data set.

Direct rate coefficient measurements were found for most of the selected reactions. Altogether, 2217 data points in 81 data sets that were used as optimization targets. Most of these direct measurements were indicated in review articles 1, 2, 3, 4, 5, 6, 7, 8, 9 as highly reliable ones. These include almost all direct measurements utilized for the optimization of our hydrogen mechanism 35. Primarily, measurements that were performed at temperatures above 700 K and below 3000 K were collected, which is the most relevant temperature range for the combustion of hydrogen and syngas. In the cases of reactions where few data were available, measurements closer to room temperature were also used. The number of direct measurements used as optimization targets for each reaction step is given in Table I, and the detailed list can be seen in Table S14 of the Supporting Information. All direct rate coefficient measurement results, together with the conditions of determinations were also encoded into XML files according to the ReSpecTh format specification 37.

## PARAMETER OPTIMIZATION

We applied our previously described global parameter optimization method 34 to the indirect and direct experimental data collected to determine the optimal values of the Arrhenius parameters and third‐body collision efficiencies. The optimal set of parameters was achieved by the minimization of the following objective function:

(1) E(p)=1N∑i=1N1Ni∑j=1NiYij mod (p)−Yij exp σ(Yij exp )2 where Yijmod/exp=yijmod/exp if σ(yijexp)≈ constant  for  all jlnyijmod/exp if σ(lnyijexp)≈ constant  for  all ji=1,...,N

Here **p** is the vector of the parameters selected for optimizations, *N* is the number of data sets, and *N_i_* is the number of data points in the *i*th data set. The values yij exp  and σ(yij exp ) are the *j*th measured (experimental) data point and its standard deviation, respectively, in the *i*th data set. For the indirect measurement data, the simulated (modeled) value is $y_{ij}^{\;\mathrm{mod}\;}$ and is obtained from a simulation using the detailed mechanism investigated. For the direct measurements, the corresponding modeled value $y_{ij}^{\;\mathrm{mod}\;}$ is calculated at a given temperature, pressure, and bath gas composition. In the formula of *E*(**p**), values, $Y_{ij}^{\;\mathrm{mod}\;}$ and $Y_{ij}^{\exp}$ were compared, which were derived from $y_{ij}^{\;\mathrm{mod}\;}$ and $y_{ij}^{\exp}$ values depending on the nature of error distribution characteristic for the type of experiment in which data set *i* was determined.

Constant *absolute* error, corresponding to identical $\sigma (y_{ij}^{\exp})$values for all data *j* within data set *i* was assumed for the measured burning velocities and concentrations; in this case $Y_{ij}=y_{ij}$ applies. Constant *relative* error, implying identical $\sigma (\ln y_{ij}^{\exp})$ values for all data *j* within data set *i* was assumed for the ignition delay measurements and the rate coefficients determined in direct experiments; in this case $Y_{ij}=\ln y_{ij}$. The standard deviations were estimated for each data set separately, based on their scatter. Within each data set, the data points were plotted as a function of the systematically varied experimental parameter. A trend line was fitted using a high‐order polynomial or spline function, and the lowest order function that already described the trend of the experimental data was used, to avoid overfitting. The standard deviation of the data points in a data set was determined by calculating the root mean square of the deviations between the data points and the fitted trend line. In order to avoid overweighting very smooth data series in the error function, a minimum standard deviation of *σ*_min_(ln *τ*) = 0.1, *σ*_min_(ln *k*) = 0.1, *σ*_min_(*S*_L_) = 2 cm/s was assigned to ignition delay, rate coefficient, and laminar burning velocity measurements, respectively. For concentration profile measurements, 1% of the maximum measured concentration was used as the minimally assigned standard deviation (i.e., *σ*_min_(*c*) = *c* ^max^/100). The estimated standard deviations for each data set are listed in Tables S1−S14 of the Supporting Information. Figures S21 and S22 in the Supporting Information show examples of residuals of the fits for laminar burning velocity measurements. These figures provide justification for choosing absolute standard deviations for these types of measurements.

As a part of our method, a global minimum search of the above error function is carried out. Global parameter optimization methods require the definition of a domain of the parameters in which the optimum is sought. Nagy et al. 33 have published temperature‐dependent uncertainty limits for the rate coefficients of the elementary reactions selected for optimization in the present work. These limits were based on an extensive review of the experimental and theoretical determinations of the rate coefficients. The objective of this was to provide an upper estimation of the uncertainties of the rate coefficients at each temperature within the combustion temperature interval. The corresponding joint uncertainty domain of the rate parameters for the selected reaction was then determined. All physically realistic Arrhenius parameters lie within these domains, and their respective limits can be used as boundaries in a global optimization. Nagy et al. 33 have also provided nonrestrictive uncertainty ranges for third‐body collision efficiencies for pressure‐dependent rate coefficients, which were also used in this work. The global minimum search of influential kinetic parameters (Arrhenius and third‐body efficiency parameters) is performed by a stochastic algorithm detailed in 34.

The evaluation of the error function requires simulations of the experiments. The simulation programs SENKIN 55, PREMIX 56, and PSR 57 of the CHEMKIN‐II package 58 were used, and the control parameters of all simulation codes were chosen so that the numerical errors were minimized, i.e. the integrator tolerances were set to strict values for all simulations. For the PREMIX calculations, the flame grid was set to contain at least 600 points and the GRAD and CURV settings were required to be less than 0.1. Typically GRAD and CURV values near 0.01–0.03 were needed to achieve a grid of 600 points. Both thermal diffusion and multicomponent diffusion models were used for the flame simulations.

The covariance matrix of the optimized parameters can be estimated 34 using the following equation:

(2) $$\begin{array}{ccc} \Sigma_{p} & = & \left[{\left(J_{o}^{T}{W\Sigma }_{Y}^{-1}J_{o}\right)}^{-1}J_{o}^{T}{W\Sigma }_{Y}^{-1}\right]\left(\Sigma_{Y}+\Sigma_{\Delta }\right)\\ & & \times \;{\left[{\left(J_{o}^{T}{W\Sigma }_{Y}^{-1}J_{o}\right)}^{-1}J_{o}^{T}{W\Sigma }_{Y}^{-1}\right]}^{T} \end{array}$$

Here $\Sigma_{p}$ is the covariance matrix of the optimized parameters, and matrices $\Sigma_{Y}$ and $\Sigma_{\Delta }$ represent the estimated statistical and the systematic errors of the experimental results, respectively. **W** is the matrix of weights of the individual data points. In accordance with Eq. (1), the off‐diagonal elements of **W** are zero, whereas the diagonal elements are 1/*N*_i_ for each data point, where *N_i_* is the number of data points in the respective data set. **J**_0_ is the Jacobian (the first derivative matrix) of the model results according to the optimized parameters at the optimal parameter set.

In our previous studies 34, 35, 38, 39, the systematic errors of the experiments were estimated based on the remaining discrepancy between the model results of the optimal model and the experimental results, using the equation $\Sigma_{\Delta }=\Delta Y\Delta Y^{T}$. In this equation, $\Delta Y={\bar{Y}}^{\;\mathrm{mod}\;}-Y^{\exp}$, and ${\bar{Y}}^{\;\mathrm{mod}\;}$ and $Y^{\exp}$ are the vectors of the optimal simulated and experimental results, respectively. In this way, the estimation of systematic errors is based on the assumption that the systematic errors average to zero over the complete set of experimental data and the “real” values of the parameters can be obtained from the full data set. However, using the expression $\Sigma_{\Delta }=\Delta Y\Delta Y^{T}$ also implies that all systematic errors are either fully correlated or anticorrelated. This can be simply demonstrated by examining an element of the $\Sigma_{\Delta }$ matrix:

(3) $$\begin{array}{c} {\left(\Sigma_{\Delta }\right)}_{ij}=\Delta Y_{i}\cdot \Delta Y_{j} \end{array}$$

where $\Delta Y_{i}$ and $\Delta Y_{j}$ are the differences between the modeled and experimental results for the *i*th and *j*th data points, respectively. If we consider that $\Sigma_{\Delta }$ is the covariance matrix representing the systematic deviations, then its elements can be written in the following form:

(4) ΣΔij=σ syst ,i·σ syst ,j·r syst ,ij=ΔYi·ΔYj

In our previous studies, σ syst ,i is approximated by $\Delta Y_{i}$, therefore according to this approximation r syst ,ij=1 and the actual value of the correlation coefficient is r syst ,ij=1, if the signs of $\Delta Y_{i}$ and $\Delta Y_{j}$ are identical, and r syst ,ij=−1, if they are different. Therefore, the estimation of the systematic errors previously used involved the assumption that all systematic errors are nearly fully correlated or anticorrelated. While it is true that the systematic errors for experimental results within one data set or several data sets measured on the same apparatus are expected to be correlated with each other, the same cannot be said for experiments carried out by different groups in different facilities. Furthermore, since the equation $\Delta Y={\bar{Y}}^{\;\mathrm{mod}\;}-Y^{\exp}$ describes the full difference between the modeled and experimental results, it also includes the differences arising from the statistical errors, which cannot be assumed to be correlated. Therefore, in our present work we used a modified estimation of the systematic errors by considering them to be uncorrelated. We still take into account the discrepancies between the simulated and experimental results by using equations ${\left(\Sigma_{\Delta }\right)}_{ii}=\Delta Y_{i}^{2}$ and ${\left(\Sigma_{\Delta }\right)}_{ij}=0$.

## CALCULATION OF RESPONSE SURFACES

As discussed in the preceding section, in Eq. (1) the modeled value is $Y_{ij}^{\;\mathrm{mod}\;}$ for the indirect measurement data and it is obtained from a simulation using the detailed mechanism with the investigated parameter set. Such a simulation is always fast for spatially homogeneous problems (i.e., for SENKIN and PSR calculations), but it can be very slow for flame simulations. Therefore, a polynomial response surface was calculated for each laminar burning velocity measurement to decrease the computational costs of the optimization. Without using response surfaces for flames, the computational cost of the optimization task would have been approximately two orders of magnitude larger, therefore would have been unfeasible. Utilization of response surfaces was not necessary for other experiment types, as ignition delay and homogeneous concentration profile measurements could be simulated sufficiently fast using the CHEMKIN‐II programs.

For each burning velocity data point, 20,000 random, uniformly distributed samples of the active parameters, previously identified using sensitivity analyses, were generated within their joint domain of uncertainty and all other parameters were fixed at their original values. The uniform sampling algorithm for the Arrhenius parameters described in 33 was used. Simulations were performed at all experimental conditions using each generated parameter set, with the strict integrator options and diffusion models described earlier. The simulation results were fitted by orthonormal polynomials using the method described in 59. Monomials were restricted to be at most fourth order and to have at most two variables of which one is at most first order. Our trial calculations indicated that the response surface polynomials generated this way are similarly accurate compared to using a full fourth‐order orthonormal polynomial expansion approximation, but they can be evaluated using less computer time.

The polynomials obtained were tested against simulation results generated from 1000 new, random sets of parameters. The maximum allowed difference between the test set of simulation results and the polynomial was 2 cm/s, which is equal to the minimum 1*σ* experimental uncertainty assigned to our data sets. For most data points, a satisfactory response surface was obtained based on this criterion. The typical root‐mean‐square error of the response surfaces was around 0.1 cm/s. Those laminar burning velocity measurements for which an accurate response surface could not be calculated were omitted from the optimization, but were included in the evaluation of the optimized models performance and comparison with other models. This meant the exclusion of 75 hydrogen and 344 syngas laminar burning velocity data points from the optimization.

There is a twofold reason why we could not obtain accurate response surfaces. In some cases, some rate parameter combinations are nonphysical and simulation results could not be obtained in these regions of the parameter space. This typically occurs when the measurements were carried out near flame extinction conditions. In other cases, we have good simulation results in the entire parameter space investigated but the surrogate model describes these points with high error. This is an issue of fitting the data, which could be avoided using a more complex fitting function.

## THE HIERARCHICAL OPTIMIZATION STRATEGY

Simultaneous optimization of a large number of parameters is a computationally challenging task. To reduce the computational costs, the same hierarchical optimization strategy was used as the one applied in our previous work 35.

For each experimental data point, the important reactions were identified, based on the normalized sensitivity coefficients and a cutoff value. Data points with identical sets of important reactions were grouped together. First, the largest group where only a single reaction was found to be important was selected, and the rate parameters of this reaction were optimized with the data points of the first group as optimization targets. Next, a new reaction was selected for optimization and all experimental data where the new and also the previously selected reaction were important were also selected as optimization targets, and the rate parameters of both reactions were optimized to them. Reactions were added one by one, initiating the usage of new experimental data groups, and the optimization steps were performed. Adding new parameters and data was repeated until all reactions and experimental data were used. The order of selected reactions was chosen in such a way that the amount of added experimental data points was always maximal for each additional reaction. The optimal order of inclusion of data sets and important reactions in this hierarchical optimization procedure was automated by a homemade code. In Table I, the order of the reactions corresponds to the inclusion order of reaction steps according to the optimization strategy.

Up to this point, the third‐body collision efficiencies were not modified, as these parameters can have identical effects to the frequency factor Arrhenius parameters at high dilutions allowing them to completely compensate each other in some cases. In the final stage of the optimization all Arrhenius parameters were fixed, and all important third‐body collision efficiencies were optimized at the same time. Little modifications were made to the third‐body collision efficiencies in this second stage, and only a small improvement of the model could be achieved.

## RESULTS AND DISCUSSION

As a result of the optimization, the error function value decreased significantly and a better description of the collected indirect and direct experimental data was achieved. Optimized values, shown in Table I, were obtained for 53 rate parameters (48 Arrhenius parameters and 5 third‐body collision efficiencies). The covariance matrix of the optimized parameters was estimated using the method described in the fifth section, which meant that the statistical scatter and the remaining discrepancies between the measurements and the modeled results were propagated to the uncertainty of the optimized parameters. From the covariance matrix of the rate parameters, temperature‐dependent uncertainty ranges were obtained for the rate coefficients of each optimized reaction. The optimized mechanism in CHEMKIN format together with the transport data file used and the covariance matrix of the Arrhenius parameters are given in the Supporting Information.

The performance of the optimized mechanism was compared to several hydrogen and syngas combustion mechanisms, and also larger mechanisms that have been validated or extensively used for the simulation of hydrogen and syngas combustion 1, 2, 3, 4, 5, 6, 7, 8, 9, 16, 29, 35, 60, 61, 62, 63, 64, 65, 66. All simulations were carried out with CHEMKIN‐II codes, without using response surfaces. All burning velocity experiments that were excluded from the optimization due to lack of accurate response surfaces were also taken into account here.

**Figure 1:**
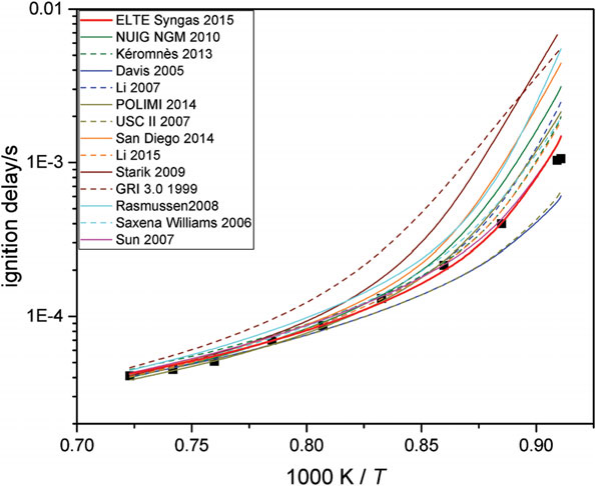
Ignition delay measurements (black squares) of Krejci et al. 67 and simulation results (lines) from each of the investigated syngas combustion mechanisms. Experimental conditions are *p* = 12 atm, *ϕ* = 0.5, H_2_/CO/O_2_/Ar = 0.005/0.005/0.01/0.98.

**Figure 2:**
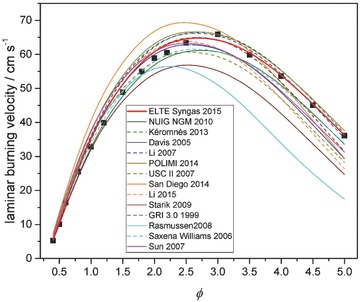
Laminar burning velocity measurements (black squares) of Bouvet et al. 68 and simulation results (lines) from each of the investigated syngas combustion mechanisms. Experimental conditions are *p* = 1 atm, *T* = 295 K, H_2_/CO = 0.5/0.95 in air.

**Figure 3:**
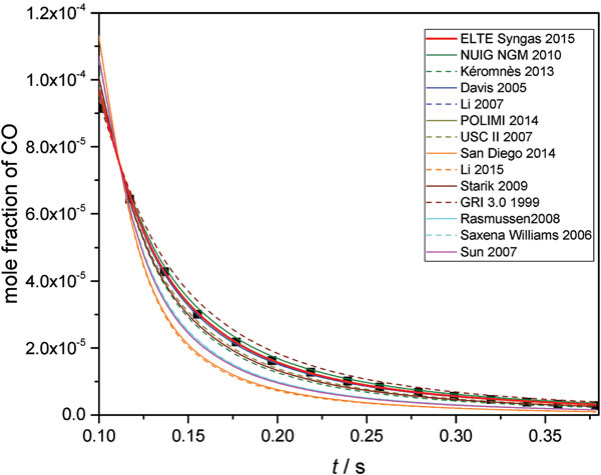
Concentration−time profile measurements (black squares) of Yetter et al. 69 and simulation results (lines) from each of the investigated syngas combustion mechanisms. Experimental conditions are *p* = 1 atm, *T* = 1138 K, *ϕ* = 0.013, CO/O_2_/H_2_O/N_2_ = 0.00016/0.0191/0.0154/0.96534.

The comparisons have been performed by investigating the error function values by experiment type for each model. Also, plots of the experimental data together with the simulation results obtained using each mechanism were created. Figures 1, 2, 3 show examples of experimental data sets and simulation results obtained with the optimized and other investigated models. Tables II and III contain the calculated error function values for each of the investigated mechanisms for the hydrogen and syngas combustion data, respectively. The error function values were also calculated separately for the ignition delay time, laminar burning velocity, and concentration profile measurements. In several burning velocity measurements, helium was used as the bath gas or as a component of the diluent mixture. Only mechanisms that contain helium as a species and have assigned third‐body collision efficiency values to pressure‐dependent reactions were used for the simulation of these experiments. In Tables II and III, the calculated error function values are given, considering the measurements where helium was not used (“noHe”) and also for the complete data set, including measurements with helium (“wHe”). Overall, there is no large difference in the reproduction of burning velocities considering the He and non‐He data, and the overall ranking of mechanisms is not affected by the selection of these groups of test data in most cases.

**Table II:** Comparison of Error Function Values between Our Optimized and 19 Other Mechanisms by Experiment Type, for Hydrogen Combustion

Hydrogen Combustion							
		Average Error Function					
Mechanism	Ref.	IDT	Conc	Flame noHe	Flame wHe	Total noHe	Total wHe
ELTE Syngas 2015	This work	6.66	4.97	7.24	5.80	**6.48**	**6.00**
ELTE Hydrogen 2015	35	6.17	5.66	6.11	4.86	**6.05**	**5.54**
Kéromnès 2013	9	8.11	8.03	5.88	8.11	**7.41**	**8.10**
NUIG NGM 2010	60	10.72	4.87	9.94	7.24	**9.25**	**8.25**
Ó Conaire 2004	4	13.00	5.33	8.90	−	**10.13**	−
Konnov 2008	5	15.17	6.73	6.37	−	**10.71**	−
Li 2015	29	13.77	6.88	15.54	10.80	**12.85**	**11.32**
Li 2007	3	18.73	5.23	7.07	7.61	**12.33**	**11.75**
Alekseev 2015	6	11.88	7.01	10.34	14.76	**10.38**	**12.19**
Hong 2011	7	10.74	5.43	18.72	−	**12.05**	−
Burke 2012	8	24.09	3.18	5.91	4.57	**14.14**	**12.34**
Saxena Williams 2006	61	22.16	15.79	8.13	7.60	**16.54**	**15.04**
POLIMI 2014	62	25.60	10.06	10.81	7.97	**17.82**	**15.58**
Davis 2005	1	36.73	3.98	7.58	5.83	**20.94**	**18.19**
Starik 2009	63	30.84	3.95	16.40	12.77	**20.77**	**18.61**
San Diego 2014	64	17.09	12.22	17.62	25.21	**16.23**	**19.56**
USC II 2007	65	36.36	3.97	13.81	−	**22.65**	−
GRI 3.0 1999	16	69.51	6.90	23.97	−	**42.43**	−
Sun 2007	2	103.10	14.48	18.60	15.31	**58.66**	**51.21**
Rasmussen 2008	66	202.58	10.60	21.23	−	**106.83**	−
No. of data sets		62	27	39	62	128	151
No. of data points		785	294	319	432	1398	1511

The error function values are normalized by the number of data sets within each column. The columns contain results for ignition delay time measurements (IDT), concentration profiles (Conc), laminar burning velocity measurements, (Flame) and over the whole data set (Total). In the case of laminar burning velocities and overall results error function values calculated both with the exclusion of experimental data where He was used as a bath gas (noHe) and including these (wHe) are given.

**Table III:** Comparison of Error Function Values between Our Optimized and 13 Other Mechanisms by Experiment Type Considering Only Syngas Combustion Data

Syngas Combustion							
		Average Error Function					
Mechanism	Ref.	IDT	Conc	Flame noHe	Flame wHe	Total noHe	Total wHe
ELTE Syngas 2015	This work	14.83	7.95	4.95	4.84	**8.43**	**8.08**
NUIG NGM 2010	60	26.52	11.72	7.59	7.84	**14.05**	**13.69**
Kéromnès 2013	9	38.09	21.34	6.60	6.29	**18.33**	**17.20**
Davis 2005	1	52.04	13.49	4.26	4.36	**20.43**	**19.19**
POLIMI 2014	62	45.28	29.17	5.49	5.89	**20.93**	**19.93**
Li 2015	29	19.80	105.73	5.27	5.92	**22.27**	**21.30**
Li 2007	3	50.77	30.11	5.58	5.79	**22.82**	**21.57**
USC II 2007	65	64.17	10.78	5.17	−	**24.41**	−
San Diego 2014	64	30.38	50.92	15.81	16.20	**24.73**	**24.25**
Starik 2009	63	36.04	75.02	15.58	14.66	**29.37**	**27.71**
GRI 3.0 1999	16	77.23	55.56	5.49	−	**34.24**	−
Rasmussen 2008	66	87.12	74.65	16.15	−	**45.70**	−
Saxena Williams 2006	61	77.51	162.54	5.31	5.39	**47.47**	**44.14**
Sun 2007	2	133.69	84.05	5.74	6.85	**55.65**	**52.32**
No. of data sets		94	37	168	194	299	325
No. of data points		938	777	1649	1879	3364	3594

The error function values are normalized by the number of data sets within each column. The columns contain results for ignition delay time measurements (IDT), concentration profiles (Conc), laminar burning velocity measurements, (Flame) and over the whole data set (Total). In the case of laminar burning velocities and overall results error function values calculated both with the exclusion of experimental data where He was used as a bath gas (noHe) and including these (wHe) are given.

The results show that the present optimized model produces the best overall results on the syngas combustion data. It is the best performing mechanism for ignition delay and concentration profile measurements, and the second best for laminar burning velocity measurements after the mechanism of Davis et al. 1.

Considering the hydrogen combustion data, a slightly worse overall performance was obtained than for our previously published, optimized hydrogen combustion mechanism 35. While the present joint optimized mechanism is not the best performing one in any of the experimental data categories for hydrogen combustion, its error function values are only slightly higher than those of the best performing previously published mechanisms.

The fact that low error function values were obtained for each experimental category considering both hydrogen and syngas combustion means that the present optimized mechanism is well balanced and provides good results in the whole validation range. This indicates that an optimization approach including multiple combustion systems over a wide range of conditions is feasible, and models that can accurately describe the combustion of several fuels simultaneously can be created by this method.

For the development of mechanisms for the combustion of more complex fuels, a similar approach could be recommended, i.e. optimization of all highly sensitive rate parameters, including those that have been optimized during the development of a less complex fuel. The most “complete” characterization of rate coefficients can be achieved in this way, as in the experimental investigation the combustion properties can carry information on reaction rate parameters that play a significant role in the combustion of less complex fuels. For example, the laminar burning velocities of most hydrocarbons are known to be largely governed by the chemistry of the hydrogen and syngas system.

However, such an approach would be hindered by the increasing computational demand, as both the number of optimization targets and the parameters to be optimized increase. A viable option is to keep the previously optimized rate parameters unmodified and to optimize only the “new” reactions that play an important role in the combustion of the more complex fuel. In this way, the computational requirements can be decreased and it can be guaranteed that a new mechanism also provides good results for the combustion of smaller fuels, since the relevant chemistry is not modified. However, in this way the joint uncertainty of the newly and previously optimized reactions cannot be investigated, and the uncertainty limits obtained for the rate coefficients will represent only the information that was obtained based on the respective subsets of experimental data.

The optimized rate coefficients were also compared to the values obtained from direct experimental and theoretical determinations and recommendations of review articles taking into consideration the calculated temperature‐dependent uncertainty ranges of the optimized rate coefficients. Figures S1–S18 of the Supporting Information show the rate coefficients of the 18 optimized reactions and their 3*σ* uncertainty limits, together with selected direct measurements and theoretical results. In the cases where a small number of recent data were available, rate coefficient expressions recommended by recent reviews were also used to provide a basis for comparison. A more comprehensive list of the corresponding rate coefficient determinations and recommendations is available in 33.

In most cases, the optimized rate coefficients are in good agreement with the most recent determinations and recommendations. This had been expected, since many of the most recent direct experimental results were used as optimization targets for the development of the present optimized mechanism. However, a key value of this work is to demonstrate that it is possible to create an optimized mechanism that can well describe both indirect experiments and direct rate coefficient determinations.

The reactions ${\dot{H}+O}_{2}=\ddot{O}\;+\;\dot{O}H\;\left(R1\right)$, ${\ddot{O}+H}_{2}=\;\dot{H}+\;\dot{O}H\;\left(R2\right)$, ${\dot{H}+O}_{2}\;{\left(+M\right)=H\dot{O}}_{2}\left(+M)(R9\right)$, and  CO +O˙H= CO 2+H˙(R24) are known to be among the most important reactions in the combustion of hydrogen and syngas and have been extensively investigated in both experimental and theoretical studies. For these reactions, very precise rate coefficient values could be determined by the optimization, since indirect measurements such as ignition delays and laminar burning velocities are usually very sensitive to these reactions. The optimized results are also consistent with the literature data for these rate coefficients.

For reactions ȮH + H_2_ = Ḣ + H_2_O (R3) and ȮH + ȮH (+M) = H_2_O_2_ (+M) (R16), a smaller number of rate coefficient determinations were available in the literature. Very narrow posterior uncertainty ranges were obtained for the rate coefficients of these reactions as a result of optimization, due to their high sensitivities at the conditions of some of the indirect experimental data. The obtained uncertainty ranges are smaller than the scatter of several independent direct determinations, and the optimized rate coefficient values fall very close to most of the recent determinations.

The rate coefficients of the reactions ȮH + ȮH = Ö + H_2_O (R4), Ḣ + Ḣ + M = H_2_ + M (R5), Ḣ + ȮH + M = H_2_O + M (R8), Ḣ + HȮ_2_ = H_2_ + O_2_ (R10), HȮ_2_ + Ḣ = ȮH + ȮH (R11), HȮ_2_ + ȮH = H_2_O + O_2_ (R13), CO + O_2_ = CO_2_ + Ö (R23), CO + HȮ_2_ = CO_2_ + ȮH (R25), HĊO + M = Ḣ + CO + M (R26), and HĊO + Ḣ = CO + H_2_ (R28) could be determined with somewhat lower uncertainties than the prior values, and an overall good agreement was obtained with the available direct determinations.

**Figure 4:**
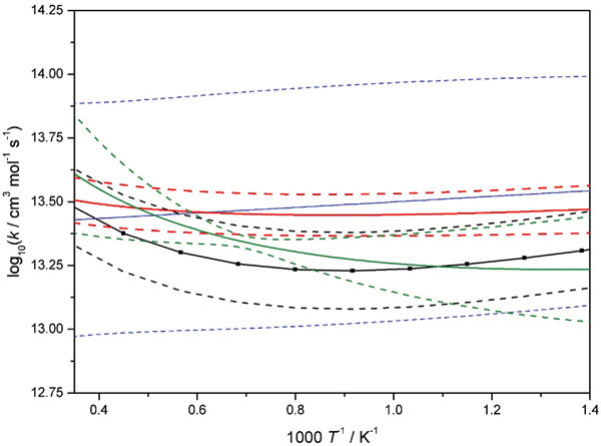
Arrhenius plot of the rate coefficient of reaction HȮ_2_ + ȮH = H_2_O + O_2_. The blue solid line shows the recommended mean value of Nagy et al. 33, the red solid line shows the result of the present optimization, and the green solid line shows the result of the optimization of Varga et al. 35. The correspondingly colored dashed lines show the respective 3*σ* uncertainty limits. The black solid line with square symbols shows the rate coefficient obtained by Burke et al. 70, and the corresponding dashed lines show the 3*σ* uncertainty bounds of Burke et al.

For the rate coefficients of the reactions Ḣ + HȮ_2_ = H_2_ + O_2_ (R10) and HȮ_2_ + Ḣ = ȮH + ȮH (R11), only few direct measurements are available. The rate coefficient values obtained here are similar to those obtained in our previous optimization study on hydrogen combustion 35, but the obtained posterior uncertainties were smaller than the prior ones. For the reactions Ḣ + ȮH + M = H_2_O + M (R8) and HȮ_2_ + ȮH = H_2_O + O_2_ (R13), somewhat different values were obtained for the rate coefficient than in our previous optimization study, although the uncertainty ranges overlap at almost all temperatures. Burke et al. 70 have recently published a detailed study of reaction (R13). They have considered both ab initio calculation results and indirect experimental data using the multiscale modeling methodology to address the inconsistencies between experimental measurements of the rate coefficient. Burke et al. found that there is a minimum of the rate coefficient near 1200 K, but it is not as sharp as it was indicated by several previous experimental results. In our previous study on hydrogen combustion 35, we obtained very similar results to those of Burke et al., although the minimum in the rate coefficient occurred at a lower temperature, around 750 K. Interestingly, in our present study a much shallower minimum was obtained compared to both our previous study and the results of Burke et al., but the position of the minimum is now near 1100 K. The Burke et al. recommendation and the previous and current optimized rate parameter functions together with their 3*σ* uncertainty limits are plotted in Fig. 4. The differences between the present optimization results and those of Varga et al. 35 are indicative of a degree of inconsistency between the sets of optimization targets. The results of direct measurements and theoretical calculations for this reaction are given in Fig. S10 of the Supporting Information.

The reaction CO + HȮ_2_ = CO_2_ + ȮH (R25) has been investigated recently in review articles and with theoretical methods 2, 71, 72, and different rate parameters have been suggested by different authors. Our obtained rate coefficient–temperature functions are in between the most recent recommendations, and all of the previously recommended rate coefficients are within our posterior uncertainty limits.

A larger number of studies were available for the reaction HĊO + M = Ḣ + CO + M (R26), and the optimized rate coefficient is near the most recent recommendations and measurements. The uncertainty of the optimized rate coefficient could be decreased compared to the prior uncertainty. The rate coefficient of reaction HĊO + Ḣ = CO + H_2_ (R28) was observed to be independent of temperature by all experimental observations, and therefore only the *A*‐factor was fitted for this reaction.

Owing to the obtained low uncertainties and overall good agreement with previous studies, we consider the optimized rate parameters of reactions (R1)−(R5), (R8)−(R11), (R13), (R16), (R23)−(R26), and (R28) as new recommended values, and not just fitted values within the context of the present optimized model.

Rather different values were obtained for the rate coefficient of reaction HȮ_2_ + HȮ_2_ = H_2_O_2_ + O_2_ (R15) compared to our previous optimization study on hydrogen. Nevertheless, in the temperature range of 800–900 K where this reaction was shown to be important in our sensitivity analysis, the two recommended rate expressions are within each other's uncertainty domains. The discrepancy at high temperatures suggests a degree of inconsistency between the H_2_ and the H_2_/CO data sets, or that the effect of changing of the rate coefficient of this reaction can be easily compensated through changes in other reactions. Also, it is entirely possible that for this reaction, indirect measurements carried out on different fuel systems will have better constraints for the rate coefficient at higher temperatures. In this case, a reevaluation of the rate coefficients could be beneficial when developing mechanisms, e.g., for more complex fuels.

The optimized rate coefficient for the reaction H_2_O_2_ + Ḣ = H_2_ + HȮ_2_ (R18) exhibits an unusual curvature at high temperatures, which does not agree with the recent theoretical determinations and recommendations. Unfortunately, no direct measurements in this temperature range are available in the literature. However, the optimized rate coefficient is very similar to that obtained in our previous optimization study 35.

For the reasons above, we conclude that the optimized rate coefficients of reactions HȮ_2_ + HȮ_2_ = H_2_O_2_ + O_2_ (R15) and H_2_O_2_ + Ḣ = H_2_ + HȮ_2_ (R18) can be used in our model to provide a very good overall reproduction of the available indirect and direct measurements of hydrogen and syngas combustion, but are not necessarily good recommended values for general use. The case of the reaction H_2_O_2_ + Ḣ = H_2_ + HȮ_2_ (R18) also demonstrates that using theoretical rate coefficient determinations in a similar way to direct measurements would most likely be beneficial. However, a comprehensive collection of such publications and an accurate assessment of the uncertainties of the theoretical methods is beyond the scope of the present paper. For more complex combustion systems, in which key elementary reactions have been studied less frequently in experiments, such an approach would be highly useful.

Figures S19–S20 of the Supporting Information show the posterior uncertainty parameter *f* as a function of temperature for each optimized reaction. The posterior uncertainties represent how precisely the rate coefficients can be determined from the available data. It is important to note that this is not only a measure of the information content of the experimental data utilized, but also refers to the degree of inconsistency between the experimental results. Further narrowing of the uncertainty ranges would primarily require resolving these inconsistencies. Note that in the present work some experimental data were not utilized based on known issues in the experimental techniques. Also, some clearly outlying data has been identified and removed from the set of optimization targets, as discussed in the second section.

## CONCLUSIONS

A joint hydrogen and syngas combustion mechanism was developed using an optimization approach, starting from the models of Kéromnès et al. 9 and Varga et al. 35. A comprehensive set of experimental data, both indirect and direct, were used as optimization targets. A significant overall improvement was achieved in the description of the syngas combustion data compared to the previously published syngas mechanisms, and a similarly good overall performance was achieved for hydrogen combustion compared to our previous optimization study 35. It was shown that the optimized mechanism provides the best performance based on the experiments used. As it provides good results in wide ranges of experimental conditions, it is a good starting point for the development of larger combustion mechanisms as well as for automatic mechanism generation.

The covariance matrix of the optimized parameters was calculated, and temperature‐dependent uncertainty ranges were obtained for the rate coefficients of each of the optimized reactions. The rate parameters of reactions (R1)−(R5), (R8)−(R11), (R13), (R16), (R23)−(R26), and (R28) could be determined with high precision, and we consider the optimized values as recommendations for the physical values (see Table I). The rate parameters of reactions (R15) and (R18) are not necessarily recommended to be used outside the current optimized model.

All collected experimental data in ReSpecTh XML data format, the optimized mechanism, and the covariance matrix of the optimized parameters are available on the ReSpecTh web site (http://respecth.hu).

## Supporting information

Disclaimer: Supplementary materials have been peer‐reviewed but not copyedited.

Supplementary Material

Supplementary Material

Supplementary Material

Supplementary Material
