# Supplementary material for: Development of a Joint Hydrogen and Syngas Combustion Mechanism Based on an Optimization Approach
Source: Int J Chem Kinet. 2016 May 20;48(8):407–22. doi: 10.1002/kin.21006 (PMC5084827; doi:10.1002/kin.21006)
Supplement: Supplementary file 1 — Supplementary Material [file KIN-48-407-s001.docx]

**Supplementary Material for**

**Development of a joint hydrogen and syngas combustion mechanism based on an optimization approach**

Tamás Varga^1,2^, Carsten Olm^1,2^, Tibor Nagy^1,3^, István Gy. Zsély^1^, Éva Valkó^1,2^,
Róbert Pálvölgyi^1^, Henry. J. Curran^4^, Tamás Turányi^1,^*

^1^Institute of Chemistry, Eötvös University (ELTE), Budapest, Hungary

^2^ MTA-ELTE Research Group on Complex Chemical Systems, Budapest, Hungary

^3^ Institute of Materials and Environmental Chemistry,

MTA Research Centre for Natural Sciences, Budapest, Hungary

^4^ Combustion Chemistry Centre, National University of Ireland, Galway (NUIG), Ireland

Tables S1 – S13 contain the summary of the indirect experimental data relevant to hydrogen and syngas combustion that were used as optimization targets and for comparison between mechanisms. The data contained in these tables are taken from the Supplementary Materials of Olm *et al.* [[1](#_ENREF_1), [2](#_ENREF_2)], with the exception of Table S6, which contains data not used by Olm *et al*.

Each row denotes a dataset for which an identifier string, the number of data points within the dataset, the assigned experimental error and the experimental conditions are given. The comment columns contain information on the exact source of the data, and additional information on the interpretation of the data if necessary. In cases where not all experimental data were used from a dataset, based on experimental considerations the number of data points used as optimization targets and in the comparison between mechanisms is given in parentheses. For laminar burning velocity measurements the experimental techniques are also given, which are the flame cone method (FCM), the outwardly propagating spherical flame method (OPF), the counterflow twin-flame technique (CTF), and the heat flux burner method (HFM). Datasets that were collected by Olm *et al.* [[1](#_ENREF_1), [2](#_ENREF_2)] but were fully excluded are not listed in the following tables.

Table S14 contains the summary of the direct experimental data that were used as optimization targets.

Figures S1-S18 show the Arrhenius plots of the rate coefficients of the 18 optimized reactions and their 3*σ* uncertainty limits, together with selected direct measurements, theoretical results. In cases where a small number of recent data was available, values recommended by recent reviews were also used to provide a basis for comparison.

Figures S19-S20 show the *f* uncertainty parameter values as a function of temperature for the 18 optimized rate coefficients.

Figures S21-S22 show the residuals of the spline fits to two laminar burning velocity datasets. These figures demonstrate the reason for choosing absolute errors over relative errors for these datasets. Similar figures could be create for all other laminar burning velocity measurement datasets.

# Utilized indirect experimental data – hydrogen combustion

Table S1. Ignition delay time measurements of hydrogen−oxygen mixtures in shock tubes

| **ID [.xml]** | ***N_i_*** | ***σ* [%]** | **Diluents** | ***φ*** | ***p* [atm]** | ***T* [K]** | **Comment** |
| --- | --- | --- | --- | --- | --- | --- | --- |
| **Pang *et al.* (2009)** [[3](#_ENREF_3)]; ignition criterion: [OH] maximum | | | | | | | |
| g00000007 | 33 (6) | 19 | Ar | 1 | 3.37–3.71 | 924–1118 | Fig. 3, solid square |
| g00000008 | 13 (2) | 18 | Ar | 0.42 | 3–3.5 | 906–1049 | Fig. 5, solid square |
| **Herzler and Naumann (2009**) [[4](#_ENREF_4)]; ignition criterion: [OH*] maximum | | | | | | | |
| g00000009 | 10 (1) | 12 | Ar | 0.5 | 1.02–1.1 | 923–1027 | Fig. 12, solid triangle |
| g00000010 | 8 (3) | 17 | Ar | 0.5 | 3.78–4.09 | 958–1035 | Fig. 12, solid circle |
| g00000011 | 12 | 10 | Ar | 0.5 | 15.13–16.37 | 1018-1121 | Fig. 12, solid square |
| g00000012 | 15 (11) | 10 | Ar | 1 | 0.87–1.13 | 918–1718 | Fig. 13, solid triangle |
| g00000013 | 13 (9) | 10 | Ar | 1 | 3.82–4.12 | 962–1160 | Fig. 13, solid circle |
| **Petersen *et al.* (1996)** [[5](#_ENREF_5)]; ignition criterion: d[OH]/d*t* maximum | | | | | | | |
| x00000065 | 14 | 10 | Ar | 1 | 33 | 1648–1855 | Fig. 3, solid circle, *X*_H2_ = 0.005 |
| x00000066 | 8 | 39 | Ar | 1 | 33 | 1189–1300 | Fig. 3, solid circle, *X*_H2_ = 0.02 |
| x00000067 | 17 | 26 | Ar | 1 | 57 | 1655–1930 | Fig. 3,crossed circle, *X*_H2_ = 0.005 |
| x00000068 | 7 | 10 | Ar | 0.99 | 64 | 1684–1779 | Fig. 3,open circle, *X*_H2_ = 0.0033 |
| x00000069 | 16 | 18 | Ar | 1 | 64 | 1361–1876 | Fig. 3,open circle, *X*_H2_ = 0.001 |
| x00000070 | 3 | 18 | Ar | 1 | 64 | 1279–1334 | Fig. 3,open circle, *X*_H2_ = 0.005 |
| x00000071 | 6 | 10 | Ar | 1 | 87 | 1701–1715 | Fig. 3,open diamond, *X*_H2_ = 0.0025 |
| **Cheng and Oppenheim (1984)** [[6](#_ENREF_6)]; ignition criterion: d*p*/d*t* maximum | | | | | | | |
| x00000356 | 57 | 24 | Ar | 1 | 1.35–2.90 | 1012–1427 | Fig. 4, open triangle |
| x10000019 | 55 | 19 | Ar | 0.5 | 1.06–2.84 | 1004–1397 | Fig. 4, open diamond |
| **Cohen and Larsen(1967)** [[7](#_ENREF_7)]; ignition criterion: d*p*/d*t* maximum | | | | | | | |
| x00000357 | 22 (19) | 27 | Ar | 1 | 0.25–1.44 | 941–1583 | Fig. 4. open circles |
| **Skinner and Ringrose (1965)** [[8](#_ENREF_8)]; ignition criterion: [OH] maximum | | | | | | | |
| x10000001 | 7 (4) | 21 | Ar | 2 | 5 | 964–1075 | Table I |
| **Schott and Kinsey (1958)** [[9](#_ENREF_9)] ; ignition criterion: [OH] maximum | | | | | | | |
| x10000002 | 17 | 33 | Ar | 0.25 | 0.74–1.99 | 1086–1836 | Fig. 3, open triangle; incident shock wave, *X*_dil_ = 0.97 |
| **Slack (1977)** [[10](#_ENREF_10)]; ignition criterion: d[OH]/d*t* maximum | | | | | | | |
| x10000005 | 16 (12) | 17 | N_2_ | 1 | 2 | 984–1184 | Fig. 2, solid circle |
| **Petersen *et al.* (2003)** [[11](#_ENREF_11)]; ignition criterion: d[OH*]/d*t* maximum | | | | | | | |
| x10000008 | 24 | 10 | Ar | 1 | 1 | 1009–1431 | Fig. 2, solid square |
| x10000016 | 4 | 14 | Ar | 1.47 | 1 | 1111–1511 | Fig. 4, solid triangle |
| **Bhaskaran *et al.* (1973)** [[12](#_ENREF_12)]; ignition criterion: d*p*/d*t* maximum | | | | | | | |
| x10000020 | 14 | 10 | N_2_ | 1 | 2.5 | 1038–1323 | Fig. 4, open circle |

| **Wang *et al.* (2003)** [[13](#_ENREF_13)]; ignition criterion: [OH] maximum | | | | | | | |
| --- | --- | --- | --- | --- | --- | --- | --- |
| x10000021 | 10 | 22 | N_2_/H_2_O | 0.42 | 4 | 1051–1272 | Fig. 8, solid square |
| x10000022 | 14 (12) | 27 | N_2_ | 0.42 | 4 | 955–1173 | Fig. 6, solid circle |
| x10000023 | 21 | 37 | N_2_/H_2_O | 0.42 | 4 | 1011–1239 | Fig. 7, solid triangle |
| x10000025 | 10 | 15 | N_2_/H_2_O | 0.42 | 9.5 | 1099–1252 | Fig. 10, open circle |
| x10000026 | 11 | 10 | N_2_/H_2_O | 0.42 | 16 | 1049–1209 | Fig. 10, asterisk |
| **Asaba *et al.* (1965)** [[14](#_ENREF_14)]; ignition criterion: [OH] maximum | | | | | | | |
| x10000027 | 12 | 32 | Ar | 0.17 | 0.23–0.41 | 1428–2320 | Fig. 6, solid circle; incident shock wave, *X*_dil_ = 0.96 |
| x10000028 | 15 | 29 | Ar | 0.5 | 0.27–0.46 | 1602–2554 | Fig. 6, open circle; incident shock wave, *X*_dil_ = 0.98 |
| **Zhang *et. al.* (2012)** [[15](#_ENREF_15)]; ignition criterion: d[OH]/d*t* maximum | | | | | | | |
| x10000031 | 10 | 47 | Ar | 0.5 | 9.87 | 1035–1222 | Fig. 9, red circle |
| x10000032 | 9 | 23 | Ar | 0.5 | 19.74 | 1011–1267 | Fig. 9, black triangle |
| **Naumann *et. al.* (2011)** [[16](#_ENREF_16)]; ignition criterion: [OH*] maximum | | | | | | | |
| x10001009 | 13 | 26 | Ar | 0.1 | 15.77–17.57 | 1037–1255 | Table 3.1, H_2_/O_2_/Ar, *φ* = 0.1, dilution 1:5, 16 bar series |
| x10001010 | 19 (14) | 10 | Ar | 0.1 | 3.36–4.15 | 935–1360 | Table 3.1, H_2_/O_2_/Ar, *φ* = 0.1, dilution 1:5, 4 bar series |
| x10001011 | 19 (17) | 12 | Ar | 0.1 | 0.73–1.32 | 939–2109 | Table 3.1, H_2_/O_2_/Ar, *φ* = 0.1, dilution 1:5, 1 bar series |
| x10001013 | 9(5) | 15 | Ar | 0.1 | 4.07–4.48 | 932–1131 | Table 3.1, H_2_/O_2_/Ar, *φ* = 0.1, dilution 1:1, 4 bar series |
| x10001014 | 19 (11) | 14 | Ar | 0.1 | 0.68–1.20 | 889–1675 | Table 3.1, H_2_/O_2_/Ar, *φ* = 0.1, dilution 1:1, 1 bar series |
| x10001015 | 16 (13) | 10 | Ar | 3.99 | 14.01–16.38 | 947–1227 | Table 3.1, H_2_/O_2_/Ar, *φ* = 0.5, dilution 1:5, 16 bar series |
| x10001016 | 20 (15) | 13 | Ar | 3.99 | 3.65–4.44 | 967–1463 | Table 3.1, H_2_/O_2_/Ar, *φ* = 0.5, dilution 1:5, 4 bar series |
| x10001017 | 26 (18) | 17 | Ar | 3.99 | 0.91–1.35 | 943–2136 | Table 3.1, H_2_/O_2_/Ar, *φ* = 0.5, dilution 1:5, 1 bar series |
| x10001018 | 10 | 13 | Ar | 0.5 | 14.90–16.05 | 1060–1243 | Table 3.1, H_2_/O_2_/Ar, *φ* = 1.0, dilution 1:5, 16 bar series |
| x10001019 | 13 | 10 | Ar | 0.5 | 3.86–4.48 | 1006–1257 | Table 3.1, H_2_/O_2_/Ar, *φ* = 1.0, dilution 1:5, 4 bar series |
| x10001020 | 11 (6) | 20 | Ar | 0.5 | 0.93–1.04 | 932–1954 | Table 3.1, H_2_/O_2_/Ar, *φ* = 1.0, dilution 1:5, 1 bar series |

Table S2. Ignition delay time measurements of hydrogen−oxygen mixtures in rapid compression machines (RCM)

| **ID [.xml]** | ***N_i_*** | ***σ*[%]** | **Diluents** | ***φ*** | ***p* [atm]** | ***T* [K]** | **Comment** |
| --- | --- | --- | --- | --- | --- | --- | --- |
| **Kéromnès *et al.* (2013)** [[17](#_ENREF_17)]; ignition criterion: d*p*/d*t* maximum | | | | | | | |
| x4000001 | 16 | 13 | Ar/N_2_ | 0.35 | 7.71–7.84 | 975–1017 | *φ* = 0.35, 8 bar series (personal communication) |
| x4000002 | 35 | 14 | Ar/N_2_ | 0.35 | 14.75–15.27 | 948–1010 | *φ* = 0.35, 16 bar series (personal communication) |
| x4000003 | 27 | 17 | Ar/N_2_ | 0.35 | 26.54–29.83 | 940–1002 | *φ*= 0.35, 32 bar series (personal communication) |
| x4000004 | 17 | 12 | Ar/N_2_ | 0.5 | 7.90–8.10 | 963–1012 | Fig. 6,black square, 8 bar series, Supplement Table S2 |
| x4000006 | 15 | 17 | Ar/N_2_ | 0.5 | 15.09–15.39 | 943–992 | Fig. 6, red circle, 16 bar series, Supplement Table S2 |
| x4000007 | 24 | 26 | Ar/N_2_ | 0.5 | 15.09–15.39 | 897–997 | Fig. 6, blue triangle, 32 bar series, Supplement Table S2, 3 additional data points (personal communication) |
| x4000008 | 10 | 36 | Ar/N_2_ | 0.5 | 30.55–32.26 | 922–970 | 32 bar series, 2^nd^ version (personal communication) |
| x4000009 | 12 | 10 | Ar/N_2_ | 0.5 | 14.62–14.82 | 1005–1056 | *φ* = 0.5, 16 bar series, dilution experiment(personal communication) |
| **Das *et al.* (2012)** [[18](#_ENREF_18)]; ignition criterion: d*p*/d*t* maximum | | | | | | | |
| x40000011 | 4 | 10 | N_2_ | 1 | 9.87 | 993–1042 | Fig. 10, open square, mixture #1 (0% H_2_O), 10 bar series |
| x40000012 | 8 | 10 | N_2_ | 1 | 29.61 | 917–1029 | Fig. 11, open square, mixture #1 (0% H_2_O), 30 bar series |
| x40000013 | 7 | 10 | N_2_ | 1 | 69.08 | 915–1010 | Fig. 12, open square, mixture #1, (0% H_2_O), 70 bar series |
| x40000014 | 5 | 10 | N_2_/H_2_O | 1 | 9.87 | 996–1048 | Fig. 10, full circle, mixture #2 (10% H_2_O), 10 bar series |
| x40000015 | 8 | 10 | N_2_/H_2_O | 1 | 29.61 | 917–1023 | Fig. 11, full circle, mixture #2 (10% H_2_O), 30 bar series |
| x40000016 | 7 | 10 | N_2_/H_2_O | 1 | 69.08 | 908–1000 | Fig. 12, full circle, mixture #2 (10% H_2_O), 70 bar series |
| x40000017 | 6 | 10 | N_2_/H_2_O | 1 | 29.61 | 927–982 | Fig. 11, open diamond, mixture #3 (40% H_2_O), 30 bar series |
| x40000018 | 6 | 10 | N_2_/H_2_O | 1 | 69.08 | 914–976 | Fig. 12, open diamond, mixture #3 (40% H_2_O), 70 bar series |
| **Mittal *et al.* (2006)** [[19](#_ENREF_19)]; ignition criterion: d*p*/d*t* maximum | | | | | | | |
| x40001002 | 5 | 10 | Ar/N_2_ | 1 | 49.35 | 963–1044 | Fig. 6, open circle, 50 bar series, 1 data point excluded (no heat loss data available) |
| x40001007 | 6 | 10 | Ar/N_2_ | 1 | 29.61 | 952–1047 | Fig. 7, open circle, 30 bar series, 2 data points excluded (no heat loss data available) |
| x40001012 | 6 | 10 | Ar/N_2_ | 1 | 14.80 | 983–1066 | Fig. 8, open circle, 15 bar series, 2 data points excluded (no heat loss data available) |

Table S3. Flow reactor measurements of hydrogen−oxygen mixtures

| **ID [.xml]** | ***N_i_*** | ***σ*** | **Diluents** | ***φ*** | ***p* [atm]** | ***T* [K]** | | **Comment** | |
| --- | --- | --- | --- | --- | --- | --- | --- | --- | --- |
| **Mueller *et al.* (1999)** [[20](#_ENREF_20)] | | | | | | | | | |
| x30000010 | 15 (57) | 6.80E–4 (H_2_) 6.98E–4 (H_2_O)  3.66E–4 (O_2_) | N_2_ | 0.97 | 3.02 | 934 | | Fig. 4 | |
| x30000011 | 0 (15) | 4.54E–3 (H_2_) 4.66E–3 (H_2_O)  2.23E–3 (O_2_) | N_2_ | 0.97 | 2.55 | 935 | | Fig. 5a | |
| x30000012 | 27 (51) | 6.33E–4 (H_2_) 8.04E–4 (H_2_O)  3.86E–4 (O_2_) | N_2_ | 0.97 | 3.44 | 933 | | Fig. 5b | |
| x30000013 | 48 (57) | 2.13E–3 (H_2_) 1.52E–4 (H_2_O)  1.30E–3 (O_2_) | N_2_ | 0.97 | 6.00 | 934 | | Fig. 5c | |
| x30000014 | 21 (30) | 4.97E–5(H_2_) 4.55E–5 (H_2_O)  2.66E–3 (O_2_) | N_2_ | 0.50 | 0.30 | 880 | | Fig. 8 | |
| x30000015 | 4 (11) | 1.03E–4 (H_2_) | N_2_ | 0.74 | 0.60 | 897 | | Fig. 10a, open circle | |
| x30000016 | 4 (10) | 1.94E–4 (H_2_) | N_2_ | 0.33 | 0.60 | 896 | | Fig. 10a, open square | |
| x30000017 | 0 (6) | 5.10E–4 (H_2_) | N_2_ | 0.97 | 2.55 | 935 | | Fig. 10b, open circle | |
| x30000018 | 7 (15) | 6.20E–4 (H_2_) | N_2_ | 0.33 | 2.50 | 943 | | Fig. 10b, open square | |
| x30000019 | 13 (15) | 1.16E–3 (H_2_) | N_2_ | 0.97 | 15.70 | 914 | | Fig. 10c, open circle | |
| x30000020 | 7 (10) | 3.16E–4 (H_2_) | N_2_ | 0.27 | 15.70 | 914 | | Fig. 10c, open square | |
| x30000021 | 0 (16) | 1.15E–2 (H_2_) | N_2_ | 0.29 | 6.50 | 884 | | Fig. 13, open circle | |
| x30000022 | 10 (17) | 5.86E–3 (H_2_) | N_2_ | 0.29 | 6.50 | 889 | | Fig. 13, open square | |
| x30000023 | 12 (17) | 1.52E–3 (H_2_) | N_2_ | 0.30 | 6.50 | 906 | | Fig. 13, open triangle | |
| x30000024 | 10 (16) | 3.68E–4 (H_2_) | N_2_ | 0.30 | 6.50 | 914 | | Fig. 13, open reverse triangle | |
| x30000025 | 7 (12) | 1.60E–4 (H_2_) | N_2_ | 0.30 | 6.50 | 934 | | Fig. 13, open diamond | |
| **Yetter *et al.*** (1991) [[21](#_ENREF_21)] | | | | | | | | | |
| x30000026 | 3 (17) | 4.96E–4 (H_2_)  1.33E–4 (O_2_) | N_2_ | 0.28 | 1 | | 910 | | Fig. 11 |

Table S4 Perfectly stirred reactor measurements of hydrogen−oxygen mixtures

| **ID [.xml]** | ***N_i_*** | ***σ*** | **Diluents** | ***φ*** | ***p* [atm]** | ***T* [K]** | **Comment** |
| --- | --- | --- | --- | --- | --- | --- | --- |
| **Le Cong and Dagaut (2009)** [[22](#_ENREF_22)] | | | | | | | |
| g00000001psr | 20 | 4.5E–4 (H_2_) 3.1E–4 (H_2_O) | N_2_ | 0.22 | 1 | 800–1050 | Fig. 1. H_2_ open diamond, H_2_O open circle |
| g00000002psr | 16 | 1.60E–3 (H_2_)  5.20E–4 (H_2_O) | N_2_ | 0.54 | 1 | 825–1000 | Fig. 2. H_2_ open diamond, H_2_O open circle |
| g00000003psr | 16 | 1.60E–3 (H_2_)  1.60E–3 (H_2_O) | N_2_ | 2.20 | 1 | 850–1025 | Fig. 3. H_2_ open diamond, H_2_O open circle |
| g00000004psr | 24 | 6.4E–4 (H_2_) 2.6E–4 (H_2_O) | N_2_ | 0.09 | 10 | 820–1150 | Fig. 4. H_2_ open diamond, H_2_O open circle |
| g00000005psr | 9 | 2.2E–4 (H_2_) 9.1E–5 (H_2_O) | N_2_ | 2.27 | 10 | 850–1150 | Fig. 5. H_2_ open diamond, H_2_O open circle, 3 H_2_O points excluded |
| g00000006psr | 16 | 8.9E–4 (H_2_) 3.2E–4 (O_2_) | N_2_/H_2_O | 0.23 | 1 | 886–1097 | Fig. 6. H_2_ open diamond, O_2_ open circle |
| g00000007psr | 14 | 8.7E–4 (H_2_) 2.3E–4 (O_2_) | N_2_/H_2_O | 0.42 | 1 | 888–1026 | Fig. 7. H_2_ open diamond, O_2_ open circle |
| g00000008psr | 16 | 2.9E–4 (H_2_) 6.9E–4 (O_2_) | N_2_/H_2_O | 1.13 | 1 | 850–1028 | Fig. 8. H_2_ open diamond, O_2_ open circle |
| g00000009psr | 18 | 4.2E–4 (H_2_) 4.5E–4 (O_2_) | N_2_/H_2_O | 2.38 | 1 | 850–1049 | Fig. 9. H_2_ open diamond, O_2_ open circle |

Table S5 Laminar flame speed measurements of hydrogen−oxygen mixtures

| **ID [.xml]** | **Type** | ***N_i_*** | ***σ* [cm/s]** | **Diluents** | | ***φ*** | ***p* [atm]** | ***T* [K]** | **Comment** |
| --- | --- | --- | --- | --- | --- | --- | --- | --- | --- |
| **Koroll *et al.* (1993)** [[23](#_ENREF_23)] | | | | | | | | | |
| gal_fl_1 | OPF | 10 (14) | 12.07 | N_2_ | | 0.15–5.56 | 0.99 | 298 | Fig. 4, solid circle |
| **Bradley *et al.* (2007)** [[24](#_ENREF_24)] | | | | | | | | | |
| gal_fl_3 | OPF | 11 (12) | 12.74 | N_2_ | | 0.30–1.00 | 0.99 | 365 | Fig. 10, open circle, 0.1 MPa |
| gal_fl_4 | OPF | 5 (7) | 7.77 | N_2_ | | 0.40–1.00 | 4.93 | 365 | Fig. 10, open circle,  0.5 MPa |
| gal_fl_5 | OPF | 5 (8) | 11.61 | N_2_ | | 0.30–1.00 | 9.87 | 365 | Fig. 10, open circle,  1 MPa |
| **Taylor (1991)** [[25](#_ENREF_25)] | | | | | | | | | |
| x00000185 | OPF | 16 | 2.00 | N_2_ | | 0.41–3.45 | 1 | 296 | Fig. 15 |
| **Takahashi *et al.* (1983)** [[26](#_ENREF_26)] | | | | | | | | | |
| x20000001 | FCM | 8 (9) | 8.53 | N_2_ | | 0.93–4.38 | 1 | 298 | Fig. 1, open circle |
| **Iijima and Takeno (1986)** [[27](#_ENREF_27)] | | | | | | | | | |
| x20000005 | OPF | 7 | 9.07 | N_2_ | | 0.53–3.94 | 1 | 291 | Fig. 7, solid circle |
| **Tse *et al.* (2000)** [[28](#_ENREF_28)] | | | | | | | | | |
| x20000002 | OPF | 16 | 4.85 | N_2_ | | 0.45–4.02 | 1 | 298 | Fig. 3, solid diamond |
| x20000007 | OPF | 11 | 3.66 | He | | 0.60–3.50 | 3 | 298 | Fig. 5a, open square |
| x20000008 | OPF | 10 | 5.15 | He | | 0.50–3.50 | 5 | 298 | Fig. 5a, open triangle |
| x20000009 | OPF | 6 | 8.25 | He | | 0.84–2.00 | 10 | 298 | Fig. 5b, open circle |
| x20000010 | OPF | 6 | 4.63 | He | | 0.85–2.00 | 15 | 298 | Fig. 5b, open square |
| x20000011 | OPF | 6 | 4.26 | He | | 0.84–2.00 | 20 | 298 | Fig. 5b, open triangle |
| **Vagelopoulos *et al.*** **(1994)** [[29](#_ENREF_29)] | | | | | | | | | |
| x20000014 | CTF | 4 (6) | 2.00 | N_2_ | 0.30–0.55 | | 1 | 298 | Fig. 7, open square |
| **Egolfopoulos and Law** **(1990)** [[30](#_ENREF_30)] | | | | | | | | | |
| x20000022 | CTF | 17 (22) | 3.10 | N_2_ | 0.25–1.49 | | 1 | 298 | Fig. 1, open square |
| x20000035 | CTF | 6 (16) | 2.32 | N_2_ | 0.15–0.60 | | 1 | 298 | Fig. 3, open square |
| x20000036 | CTF | 6 (8) | 2.00 | N_2_ | 0.51–1.25 | | 1 | 298 | Fig. 4, open square |
| x20000037 | CTF | 8 (10) | 4.42 | N_2_ | 0.80–2.20 | | 1 | 298 | Fig. 5, open square |
| **Hermanns *et al.* (2007)** [[31](#_ENREF_31)] | | | | | | | | | |
| x20000023 | HFM | 19 (29) | 2.00 | N_2_ | 0.80–3.30 | | 1 | 298 | Fig. 4, solid circle |
| **Lamoreux *et al.* (2003)** [[32](#_ENREF_32)] | | | | | | | | | |
| x20000025 | OPF | 12 (15) | 4.19 | N_2_ | 0.26–3.57 | | 1 | 298 | Fig. 11, open circle |
| **Aung *et al.* (1998)** [[33](#_ENREF_33)] | | | | | | | | | |
| x20000027 | OPF | 10 | 6.81 | N_2_ | 0.45–4.00 | | 0.5 | 298 | Fig. 5 center, solid circle |
| x20000028 | OPF | 10 | 5.90 | N_2_ | 0.45–4.00 | | 1 | 298 | Fig. 5 top, solid circle |
| x20000029 | OPF | 9 (10) | 6.82 | N_2_ | 0.45–4.00 | | 2 | 298 | Fig. 5 top, solid circle |
| x20000030 | OPF | 9 (10) | 6.86 | N_2_ | 0.45–4.00 | | 3 | 298 | Fig. 6 center, solid circle |
| x20000031 | OPF | 7 (8) | 23.50 | N_2_ | 0.45–4.00 | | 4 | 298 | Fig. 6 top, solid circle |
| x20000034 | OPF | 12 | 2.32 | N_2_ | 0.60–4.00 | | 1 | 298 | Fig. 7 upper center, solid circle |

| **Kwon and Faeth (2001)** [[34](#_ENREF_34)] | | | | | | | | |
| --- | --- | --- | --- | --- | --- | --- | --- | --- |
| x20000038 | OPF | 9 | 4.42 | N_2_ | 0.60–4.50 | 1 | 298 | Table 1 |
| x20000039a | OPF | 4 | 19.93 | He | 0.60 | 0.5–3.0 | 298 | Table 3, *φ* = 0.6, *p* varied |
| x20000039b | OPF | 6 | 23.94 | He | 0.90–3.75 | 1 | 298 | Table 3, *p* = 1 atm,  *φ* varied |
| x20000039c | OPF | 3 | 7.50 | He | 4.50 | 1–3 | 298 | Table 3, *φ* = 4.5, *p* varied |
| x20000040a | OPF | 3 | 2.00 | Ar | 0.60 | 0.3–1.0 | 298 | Table 2, *φ* = 0.6, *p* varied |
| x20000040b | OPF | 7 | 6.34 | Ar | 0.90–3.75 | 1 | 298 | Table 2, *p* = 1 atm,  *φ* varied |
| x20000040c | OPF | 4 | 2.41 | Ar | 4.5 | 0.5–3.0 | 298 | Table 2, *φ* = 4.5, *p* varied |
| x20000040d | OPF | 4 | 30.30 | Ar | 4.5 | 1 | 298 | Table 2, *φ* = 4.5, O_2_/(O_2_+Ar) varied |
| x20000040e | OPF | 5 | 5.62 | Ar | 0.6 | 1 | 298 | Table 2, *φ* = 0.6, O_2_/(O_2_+Ar) varied |
| **Burke *et al.* (2010)** [[35](#_ENREF_35)] | | | | | | | | |
| x20000041 | OPF | 4 (6) | 4.57 | He | 0.85 | 1–25 | 295 | Suppl. Mat. Table A1,  Fig. 4, solid square |
| x20000042 | OPF | 3 | 9.52 | He | 1 | 1–10 | 295 | Suppl. Mat. Table A1,  Fig. 6, red diamond |
| x20000043 | OPF | 2 (6) | 3.57 | He | 1 | 1–25 | 295 | Suppl. Mat. Table A1,  Fig. 6, black square |
| x20000044 | OPF | 6 | 2.40 | He | 1 | 1–25 | 295 | Suppl. Mat. Table A1,  Fig. 6, brown circle |
| x20000045 | OPF | 5 | 5.31 | He | 1 | 1–20 | 295 | Suppl. Mat. Table A1,  Fig. 6, blue triangle |
| x20000046 | OPF | 5 | 2.00 | Ar | 2.5 | 1–20 | 295 | Suppl. Mat. Table A1,  Fig. 7, red diamond |
| x20000047 | OPF | 6 | 2.47 | Ar | 2.5 | 1–25 | 295 | Suppl. Mat. Table A1,  Fig. 7, black square |
| x20000048 | OPF | 5 (6) | 2.13 | Ar | 2.5 | 1–25 | 295 | Suppl. Mat. Table A1,  Fig. 7, brown circle |
| x20000049 | OPF | 2 | 4.00 | Ar | 2.5 | 1–5 | 295 | Suppl. Mat. Table A1,  Fig. 7, blue triangle |
| **Tang *et al.* (2008)** [[36](#_ENREF_36)] | | | | | | | | |
| x20000050 | OPF | 6 | 8.47 | N_2_ | 0.60–1.60 | 1 | 298 | Fig. 8, solid triangle |
| **Huang *et al.* (2006)** [[37](#_ENREF_37)] | | | | | | | | |
| x20000052 | OPF | 9 | 3.62 | N_2_ | 0.60–1.40 | 1 | 298 | Fig. 9, solid circle |
| **Burke *et al.* (2011)** [[38](#_ENREF_38)] | | | | | | | | |
| x20000053 | OPF | 2 (4) | 2.16 | He | 0.3 | 1–10 | 295 | Electr. Suppl. Table S1. Fig. 3, solid square |
| x20000054 | OPF | 1 | 2.00 | He | 0.3 | 5 | 295 | Electr. Suppl. Table S1 |
| x20000055 | OPF | 2 (5) | 2.00 | He | 0.5 | 1–10 | 295 | Electr. Suppl. Table S1 |
| x20000056 | OPF | 3 | 2.00 | He | 0.5 | 1–10 | 295 | Electr. Suppl. Table S1 |
| x20000057 | OPF | 4 (5) | 2.00 | He | 0.7 | 1–10 | 295 | Electr. Suppl. Table S1. Fig. 1 bottom, solid square |
| x20000058 | OPF | 4 (6) | 2.00 | He | 0.7 | 1–25 | 295 | Electr. Suppl. Table S1. Fig. 1 top, solid square |
| x20000059 | OPF | 3 | 2.00 | He | 0.7 | 1–10 | 295 | Electr. Suppl. Table S1. |

| **Qiao *et al.* (2007)** [[39](#_ENREF_39)] | | | | | | | | | |
| --- | --- | --- | --- | --- | --- | --- | --- | --- | --- |
| x20000060 | OPF | 8 | 3.25 | He/N_2_ | 1 | | 1 | 298 | Fig. 3, solid inverse triangle |
| x20000061 | OPF | 8 (12) | 2.00 | Ar/N_2_ | 1 | | 1 | 298 | Fig. 3, solid triangle |
| x20000062 | OPF | 8 (10) | 2.97 | N_2_ | 1 | | 1 | 298 | Fig. 3, solid circle |
| x20000064 | OPF | 9 (11) | 5.45 | Ar/N_2_ | 1.8 | | 1 | 298 | Fig. 4, solid triangle |
| x20000067 | OPF | 4 (11) | 3.87 | N_2_ | 1 | | 0.5 | 298 | Fig. 5, open circle |
| **Dong *et al.* (2009)** [[40](#_ENREF_40)] | | | | | | | | | |
| x20000074 | FCM | 17 (18) | 7.52 | N_2_ | 0.40–2.10 | | 1 | 298 | Fig. 6, open blue diamond |
| **Santner *et al.* (2013)** [[41](#_ENREF_41)] | | | | | | | | | |
| x20000075 | OPF | 3 | 3.64 | He | | 0.85 | 1–10 | 393 | Fig. 2a, open orange triangle, calculated from mass burning rate, no H_2_O dilution |
| x20000076 | OPF | 7 | 2.00 | He/H_2_O | | 0.85 | 2–10 | 393 | Fig. 2a, open blue diamond, calculated from mass burning rate, 5% H_2_O dilution |
| x20000077 | OPF | 4 | 4.36 | He/H_2_O | | 0.85 | 1–4 | 393 | Fig. 2a, open green circle, calculated from mass burning rate, 15% H_2_O dilution |

**Table S6.** Flow reactor outlet concentration measurements of hydrogen−oxygen mixtures

| **ID [.xml]** | ***N_i_*** | ***σ*** | **Diluents** | ***φ*** | ***p* [atm]** | ***T* [K]** | **Comment** |
| --- | --- | --- | --- | --- | --- | --- | --- |
| **Hashemi *et al.* (2009)** [[42](#_ENREF_42)] | | | | | | | |
| x30000027 | 9 | 1.5E–4 (H_2_) 9.6E–5 (O_2_) | N_2_ | 12.07 | 49.3 | 700–900 | Suppl. Mat. Table S2,  Fig. 1 |
| x30000028 | 9 | 1.8E–4 (H_2_) 9.8E–5 (O_2_) | N_2_ | 1.03 | 49.3 | 700–900 | Suppl. Mat. Table S3,  Fig. 2 |
| x30000029 | 9 | 9.7E–5 (H_2_) | N_2_ | 0.05 | 49.3 | 700–900 | Suppl. Mat. Table S4,  Fig. 3 |
| x30000030 | 9 | 1.6E–4 (H_2_) | N_2_ | 0.0009 | 49.3 | 700–900 | Suppl. Mat. Table S5,  Fig. 3 |

# Utilized indirect experimental data – syngas combustion

**Table S7**. Ignition delay time measurements of syngas−oxygen mixtures in shock tubes

| **ID [.xml]** | ***N*_i_** | ***σ* (ln*τ*)** | **Diluents** | ***φ*** | ***p* / atm** | ***T* / K** | **Comment** |
| --- | --- | --- | --- | --- | --- | --- | --- |
| **Petersen et al*.* (2007)** [[43](#_ENREF_43)]; ignition criterion: d*p*/d*t* maximum | | | | | | | |
| x10001001 | 13 (8) | 0.18 | N_2_/CO_2_ | 0.5 | 16.5–32.7 | 943–1148 | Table 1, top, in air |
| **Naumann et al. (2011)** [[16](#_ENREF_16)]; ignition criterion: [OH*] maximum | | | | | | | |
| x10001022 | 15 (14) | 0.10 | Ar | 1 | 3.51–4.25 | 991–1934 | Table 3.1, H_2_/CO/O_2_/Ar , *φ*=1.0, dilution 1:5, H_2_/CO=5/95, 4 bar series |
| x10001023 | 22 (17) | 0.16 | Ar | 1 | 0.85–1.14 | 957–2374 | Table 3.1, H_2_/CO/O_2_/Ar, *φ*=1.0, dilution 1:5, H_2_/CO=5/95, 1 bar series |
| x10001025 | 23 (19) | 0.16 | Ar | 1 | 3.79–5.07 | 943–1364 | Table 3.1, H_2_/CO/O_2_/Ar, *φ*=1.0, dilution 1:5, H_2_/CO=50/50, 4 bar series |
| x10001026 | 23 (18) | 0.17 | Ar | 1 | 0.84–1.18 | 924–2220 | Table 3.1, H_2_/CO/O_2_/Ar, *φ*=1.0, dilution 1:5, H_2_/CO=50/50, 1 bar series |
| x10001028 | 16 | 0.24 | Ar | 0.5 | 3.68–4.56 | 1042–1824 | Table 3.1, H_2_/CO/O_2_/Ar, *φ*=0.5, dilution 1:5, H_2_/CO=5/95, 4 bar series |
| x10001029 | 13 (11) | 0.14 | Ar | 0.5 | 0.89–1.17 | 943–2246 | Table 3.1, H_2_/CO/O_2_/Ar, *φ*=0.5, dilution 1:5, H_2_/CO=5/95, 1 bar series |
| x10001030 | 15 | 0.14 | Ar | 0.5 | 15.06–17.16 | 1007–1249 | Table 3.1, H_2_/CO/O_2_/Ar, *φ*=0.5, dilution 1:5, H_2_/CO=50/50, 16 bar series |
| x10001031 | 26 (23) | 0.10 | Ar | 0.5 | 3.6–4.83 | 970–1418 | Table 3.1, H_2_/CO/O_2_/Ar, *φ*=0.5, dilution 1:5, H_2_/CO=50/50, 4 bar series |
| x10001032 | 37 (26) | 0.17 | Ar | 0.5 | 0.82–1.12 | 882–2031 | Table 3.1, H_2_/CO/O_2_/Ar, *φ*=0.5, dilution 1:5, H_2_/CO=50/50, 1 bar series |
| **Kalitan et al. (2007)** [[44](#_ENREF_44)]; ignition criterion: d[OH*]/d*t* maximum | | | | | | | |
| x10001033 | 19 (10) | 0.12 | N_2_ | 0.5 | 0.9–1.2 | 916–1151 | Table 2, H_2_/CO=80/20 (mixture 1), low pressure series, in air |
| x10001034 | 9 (6) | 0.18 | N_2_ | 0.5 | 1.0–1.1 | 914–1241 | Table 2, H_2_/CO=60/40 (mixture 2), low pressure series, in air |
| x10001035 | 14 (10) | 0.16 | N_2_ | 0.5 | 0.9–1.1 | 900–1169 | Table 2, H_2_/CO=40/60 (mixture 3)  low pressure series, in air |
| x10001036 | 14 (7) | 0.16 | N_2_ | 0.5 | 1.1–1.2 | 934–1183 | Table 2, H_2_/CO=20/80 (mixture 4), low pressure series, in air |
| x10001037 | 11 (6) | 0.22 | N_2_ | 0.5 | 2.5–3.1 | 929–1063 | Table 2, H_2_/CO=20/80 (mixture 4), mid pressure series, in air |
| x10001038 | 6 | 0.15 | N_2_ | 0.5 | 13.7–17.9 | 1015–1190 | Table 2, H_2_/CO=20/80 (mixture 4), high pressure series, in air |
| x10001039 | 10 (8) | 0.10 | N_2_ | 0.5 | 1.1–1.2 | 960–1197 | Table 2, H_2_/CO=10/90 (mixture 5),  low pressure series, in air |
| x10001040 | 12 (10) | 0.13 | N_2_ | 0.5 | 2.1–2.7 | 981–1148 | Table 2, H_2_/CO=10/90 (mixture 5)  mid pressure series, in air |
| x10001041 | 10 | 0.10 | N_2_ | 0.5 | 11.9–17.1 | 1063–1265 | Table 2, H_2_/CO=10/90 (mixture 5), high pressure series, in air |
| x10001042 | 18 (14) | 0.13 | N_2_ | 0.5 | 1.0–1.2 | 968–1263 | Table 2, H_2_/CO=5/95 (mixture 6), low pressure series, in air |
| x10001043 | 13 (12) | 0.10 | N_2_ | 0.5 | 1.7–2.3 | 977–1304 | Table 2, H_2_/CO=5/95 (mixture 6), mid pressure series, in air |
| x10001044 | 8 | 0.25 | N_2_ | 0.5 | 12.7–19.2 | 1074–1163 | Table 2, H_2_/CO=5/95 (mixture 6), high pressure series, in air |
| **Dean and Johnson (1980)** [[45](#_ENREF_45)]; ignition criterion: measurement-specific CO_2_ level | | | | | | | |
| x10001049 | 9 | 0.10 | Ar | 1.65 | 1.2–1.9 | 2050–2810 | Table 2, mixture C, *τ*_1_ definition |
| x10001050 | 9 | 0.10 | Ar | 1.65 | 1.2–1.9 | 2050–2810 | Table 2, mixture C, *τ*_2_ definition |
| x10001051 | 13 | 0.10 | Ar | 6.11 | 1.4–2.2 | 2070–2869 | Table 2, mixture D, *τ*_1_ definition |
| x10001052 | 13 | 0.10 | Ar | 6.11 | 1.4–2.2 | 2070–2869 | Table 2, mixture D, *τ*_2_ definition |

| **Herzler and Naumann (2008)** [[46](#_ENREF_46)]; ignition criterion: [OH*] maximum | | | | | | | |
| --- | --- | --- | --- | --- | --- | --- | --- |
| x10001053 | 7 | 0.10 | Ar | 0.5 | 14.31–16.68 | 1046–1132 | Table 2, H_2_/CO/O_2_/Ar, *φ*=0.5, dilution 1:2, H_2_/CO=5/95, 16 bar series |
| x10001054 | 5 | 0.10 | Ar | 0.5 | 15.49–16.19 | 1107–1206 | Table 2, H_2_/CO/O_2_/Ar, *φ*=0.5, dilution 1:5, H_2_/CO=5/95, 16 bar series |
| x10001055 | 5 | 0.10 | Ar | 0.5 | 15.69–16.19 | 1165–1259 | Table 2, H_2_/CO/O_2_/Ar, *φ*=0.5, dilution 1:10, H_2_/CO=5/95, 16 bar series |
| x10001056 | 5 | 0.18 | Ar | 0.5 | 13.92–15.59 | 1019–1097 | Table 2, H_2_/CO/O_2_/Ar, *φ*=0.5, dilution 1:2, H_2_/CO=50/50, 16 bar series |
| x10001057 | 7 | 0.15 | Ar | 0.5 | 15.10–15.79 | 1048–1128 | Table 2, H_2_/CO/O_2_/Ar, *φ*=0.5, dilution 1:5, H_2_/CO=50/50, 16 bar series |
| x10001058 | 6 | 0.10 | Ar | 0.5 | 15.30–15.69 | 1054–1140 | Table 2, H_2_/CO/O_2_/Ar, *φ*=0.5, dilution 1:10, H_2_/CO=50/50, 16 bar series |
| **Krejci et al. (2012)** [[47](#_ENREF_47)]; ignition criterion: d[OH*]/d*t* maximum | | | | | | | |
| x10001059 | 12 (11) | 0.10 | Ar | 0.5 | 1.48–1.79 | 982–1702 | Table A5, H_2_/CO/O_2_/Ar= 0.008/0.002/0.01/0.98,  low pressure series |
| x10001060 | 8 | 0.23 | Ar | 0.5 | 11.8–12.8 | 1096–1298 | Table A5, H_2_/CO/O_2_/Ar= 0.008/0.002/0.01/0.98,  mid pressure series |
| x10001061 | 5 | 0.10 | Ar | 0.5 | 30.4–32.3 | 1180–1262 | Table A5, H_2_/CO/O_2_/Ar= 0.008/0.002/0.01/0.98,  high pressure series |
| x10001062 | 15 (14) | 0.10 | Ar | 0.5 | 1.38–1.86 | 991–2001 | Table A6, H_2_/CO/O_2_/Ar= 0.005/0.005/0.01/0.98,  low pressure series |
| x10001063 | 10 | 0.10 | Ar | 0.5 | 11.2–12.7 | 1098–1383 | Table A6, H_2_/CO/O_2_/Ar= 0.005/0.005/0.01/0.98,  mid pressure series |
| x10001064 | 6 | 0.25 | Ar | 0.5 | 31.7–32.8 | 1174–1326 | Table A6, H_2_/CO/O_2_/Ar= 0.005/0.005/0.01/0.98,  high pressure series |
| x10001065 | 10 | 0.10 | Ar | 0.5 | 1.52–1.94 | 1017–1845 | Table A7, H_2_/CO/O_2_/Ar= 0.004/0.006/0.01/0.98,  low pressure series |
| x10001066 | 13 | 0.10 | Ar | 0.5 | 11.9–13.3 | 1092–1442 | Table A7, H_2_/CO/O_2_/Ar= 0.004/0.006/0.01/0.98,  mid pressure series |
| x10001067 | 8 | 0.10 | Ar | 0.5 | 30.6–32.2 | 1143–1300 | Table A7, H_2_/CO/O_2_/Ar= 0.004/0.006/0.01/0.98,  high pressure series |
| x10001068 | 8 | 0.10 | Ar | 0.5 | 1.37–1.90 | 1057–1923 | Table A8, H_2_/CO/O_2_/Ar= 0.002/0.008/0.01/0.98,  low pressure series |
| x10001069 | 8 | 0.21 | Ar | 0.5 | 11.8–12.9 | 1097–1388 | Table A8, H_2_/CO/O_2_/Ar= 0.002/0.008/0.01/0.98,  mid pressure series |
| x10001070 | 6 | 0.17 | Ar | 0.5 | 30.3–32.2 | 1179–1320 | Table A8, H_2_/CO/O_2_/Ar= 0.002/0.008/0.01/0.98,  high pressure series |
| x10001071 | 15 | 0.10 | Ar | 0.5 | 1.41–1.97 | 1026–1991 | Table A9, H_2_/CO/O_2_/Ar= 0.001/0.009/0.01/0.98,  low pressure series |
| x10001072 | 9(8) | 0.10 | Ar | 0.5 | 11.0–13.0 | 1116–1388 | Table A9, H_2_/CO/O_2_/Ar= 0.001/0.009/0.01/0.98,  mid pressure series |
| x10001073 | 6 | 0.23 | Ar | 0.5 | 30.2–32.3 | 1161–1329 | Table A9, H_2_/CO/O_2_/Ar= 0.001/0.009/0.01/0.98,  high pressure series |

| **Thi et al. (2014)** [[48](#_ENREF_48)] – using volume-time histories; ignition criterion: d[OH*]/d*t* maximum | | | | | | | |
| --- | --- | --- | --- | --- | --- | --- | --- |
| x10001074 | 14 | 0.10 | Ar | 1 | 1.50–1.88 | 902–1161 | Suppl. Mat. Table 2, SH70 (mixture 1), 0.2 MPa series |
| x10001075 | 11 | 0.13 | Ar | 1 | 9.10–9.98 | 1005–1264 | Suppl. Mat. Table 2, SH70 (mixture 1), 1.0 MPa series |
| x10001076 | 9 | 0.21 | Ar | 1 | 17.24–20.36 | 1024–1183 | Suppl. Mat. Table 2, SH70 (mixture 1), 2.0 MPa series |
| x10001077 | 11 | 0.10 | Ar | 1 | 1.73–1.95 | 896–1201 | Suppl. Mat. Table 2, SH33 (mixture 2), 0.2 MPa series |
| x10001078 | 11 | 0.10 | Ar | 1 | 8.54–10.21 | 1012–1203 | Suppl. Mat. Table 2, SH33 (mixture 2), 1.0 MPa series, **data point at 19.80 atm moved to x10001079.xml** |
| x10001079 | 6 | 0.28 | Ar | 1 | 19.24–20.49 | 1049–1234 | Suppl. Mat. Table 2, SH33 (mixture 2), 2.0 MPa series |
| x10001080 | 11 | 0.10 | Ar/CO_2_ | 1 | 1.79–1.97 | 916–1200 | Suppl. Mat. Table 2, SH50C30 (mixture 3), 0.2 MPa series |
| x10001081 | 10 | 0.10 | Ar/CO_2_ | 1 | 8.17–10.22 | 1016–1258 | Suppl. Mat. Table 2, SH50C30 (mixture 3), 1.0 MPa series |
| x10001082 | 9 | 0.14 | Ar/CO_2_ | 1 | 19.26–20.48 | 1051–1239 | Suppl. Mat. Table 2, SH50C30 (mixture 3), 2.0 MPa series |
| x10001083 | 12 | 0.10 | Ar/N_2_ | 1 | 1.78–1.98 | 925–1150 | Suppl. Mat. Table 2, SH26N58 (mixture 4), 0.2 MPa series |
| x10001084 | 11 | 0.10 | Ar/N_2_ | 1 | 9.55–10.25 | 1023–1308 | Suppl. Mat. Table 2, SH26N58 (mixture 4), 1.0 MPa series |
| x10001085 | 7 | 0.10 | Ar/N_2_ | 1 | 19.42–20.53 | 1088–1244 | Suppl. Mat. Table 2, SH26N58 (mixture 4), 2.0 MPa series |

**Table S8** Ignition delay time measurements of hydrogen−oxygen mixtures in rapid compression machines (RCM)

| **ID [.xml]** | ***N*_i_** | ***σ* (ln*τ*)** | **Diluents** | ***φ*** | ***p* / atm** | ***T* / K** | **Comment** |
| --- | --- | --- | --- | --- | --- | --- | --- |
| **Mittal et al. (2006)** [[19](#_ENREF_19)] − only those data points were used for which pressure profiles were provided  by the authors; ignition criterion: d*p*/d*t* maximum | | | | | | | |
| x40001003 | 1 (5) | 0.10 | Ar/N_2_ | 1.0 | 49.35 | 964–1044 | Fig. 6, open square, CO/H_2_=0.33 |
| x40001004 | 1 (5) | 0.15 | Ar/N_2_ | 1.0 | 49.35 | 964–1044 | Fig. 6, open diamond, CO/H_2_=1 |
| x40001005 | 1 (4) | 0.10 | Ar/N_2_ | 1.0 | 49.35 | 984–1044 | Fig. 6, open triangle, CO/H_2_=1.86 |
| x40001006 | 1 (4) | 0.13 | Ar/N_2_ | 1.0 | 49.35 | 984–1044 | Fig. 6, open reverse triangle, CO/H_2_=4 |
| x40001008 | 1 (5) | 0.10 | Ar/N_2_ | 1.0 | 29.61 | 979–1046 | Fig. 7, open square, CO/H_2_=0.33 |
| x40001009 | 1 (5) | 0.14 | Ar/N_2_ | 1.0 | 29.61 | 979–1046 | Fig. 7, open diamond, CO/H_2_=1 |
| x40001010 | 1 (4) | 0.10 | Ar/N_2_ | 1.0 | 29.61 | 994–1046 | Fig. 7, open triangle, CO/H_2_=1.86 |
| x40001011 | 1 (4) | 0.14 | Ar/N_2_ | 1.0 | 29.61 | 994–1046 | Fig. 7, open reverse triangle, CO/H_2_=4 |
| x40001013 | 1 (5) | 0.12 | Ar/N_2_ | 1.0 | 14.8 | 977–1046 | Fig. 8, open square, CO/H_2_=0.33 |
| x40001014 | 1 (5) | 0.12 | Ar/N_2_ | 1.0 | 14.8 | 977–1046 | Fig. 8, open diamond, CO/H_2_=1 |
| x40001015 | 1 (4) | 0.10 | Ar/N_2_ | 1.0 | 14.8 | 994–1046 | Fig. 8, open triangle, CO/H_2_=1.86 |
| x40001016 | 1 (4) | 0.10 | Ar/N_2_ | 1.0 | 14.8 | 994–1046 | Fig. 8, open reverse triangle, CO/H_2_=4 |
| **Kéromnès et al. (2013)** [[17](#_ENREF_17)]; ignition criterion: d*p*/d*t* maximum | | | | | | | |
| x40001031 | 15 | 0.10 | Ar/N_2_ | 0.35 | 7.8–7.9 | 989–1036 | H_2_/CO=85/15, *φ*=0.35, 8 bar series |
| x40001033 | 8 | 0.10 | Ar/N_2_ | 0.35 | 29.3–30.1 | 952–977 | H_2_/CO=85/15, *φ*=0.35, 32 bar series |
| x40001034 | 14 | 0.11 | Ar/N_2_ | 0.5 | 7.8–7.9 | 976–1030 | H_2_/CO=85/15, *φ*=0.5, 8 bar series |
| x40001035 | 21 | 0.10 | Ar/N_2_ | 0.5 | 15.1–15.3 | 960–1033 | H_2_/CO=85/15, *φ*=0.5, 16 bar series |
| x40001036 | 15 | 0.23 | Ar/N_2_ | 0.5 | 29.6–33.0 | 940–984 | H_2_/CO=85/15, *φ*=0.5, 32 bar series |
| x40001037 | 12 | 0.10 | Ar/N_2_ | 0.5 | 15.5–15.6 | 1021–1062 | H_2_/CO=85/15, *φ*=0.5, dilution experiment,16 bar series |
| x40001039 | 18 | 0.10 | Ar/N_2_ | 0.35 | 15.4–15.5 | 971–1028 | H_2_/CO=50/50, *φ*=0.35, 16 bar series |
| x40001040 | 15 | 0.18 | Ar/N_2_ | 0.35 | 29.9–30.1 | 954–997 | H_2_/CO=50/50, *φ*=0.35, 32 bar series |
| x40001041 | 13 | 0.12 | Ar/N_2_ | 0.5 | 8.0–8.1 | 978–1022 | H_2_/CO=50/50, *φ*=0.5, 8 bar series |
| x40001042 | 21 | 0.17 | Ar/N_2_ | 0.5 | 15.2–15.3 | 951–1021 | H_2_/CO=50/50, *φ*=0.5, 16 bar series |
| x40001043 | 28 | 0.19 | Ar/N_2_ | 0.5 | 29.5–30.8 | 933–990 | H_2_/CO=50/50, *φ*=0.5, 32 bar series |
| x40001045 | 12 | 0.16 | Ar/N_2_ | 0.35 | 8.3–8.6 | 1018–1053 | H_2_/CO=25/75, *φ*=0.35, 8 bar series |
| x40001046 | 21 | 0.15 | Ar/N_2_ | 0.35 | 15.5–16.0 | 986–1057 | H_2_/CO=25/75, *φ*=0.35, 16 bar series |
| x40001047 | 13 | 0.14 | Ar/N_2_ | 0.35 | 29.2–31.2 | 971–1018 | H_2_/CO=25/75, *φ*=0.35, 32 bar series |
| x40001048 | 10 | 0.10 | Ar/N_2_ | 0.5 | 8.5–8.6 | 1010–1045 | H_2_/CO=25/75, *φ*=0.5, 8 bar series |
| x40001049 | 21 | 0.10 | Ar/N_2_ | 0.5 | 15.7–15.9 | 974–1045 | H_2_/CO=25/75, *φ*=0.5, 16 bar series |
| x40001050 | 10 | 0.16 | Ar/N_2_ | 0.5 | 31.0–31.2 | 963–999 | H_2_/CO=25/75, *φ*=0.5, 32 bar series |
| x40001052 | 12 | 0.21 | Ar/N_2_ | 0.35 | 14.9–15.0 | 1022–1057 | H_2_/CO=5/95, *φ*=0.35, 16 bar series |
| x40001053 | 12 | 0.15 | Ar/N_2_ | 0.35 | 29.0–29.2 | 98–1042 | H_2_/CO=5/95, φ=0.35, 32 bar series |
| x40001055 | 20 | 0.13 | Ar/N_2_ | 0.5 | 14.8–15.0 | 979–1049 | H_2_/CO=5/95, *φ*=0.5, 16 bar series |
| **Mansfield and Wooldridge (2014)** [[49](#_ENREF_49)] − only those data points were used for which pressure profiles were  provided by the authors, ***σ* values had to be assumed**;  ignition criterion: 50% of (*p*_min_ + *p*_max_) after end of compression | | | | | | | |
| x40001057 | 5 | 0.15 | N_2_/Ar/CO_2_ | 0.1 | 13.0–16.6 | 1066–1145 | Suppl. Mat. Table A, 15 atm series |
| x40001059 | 2 | 0.15 | N_2_/CO_2_ | 0.1 | 4.4–5.7 | 1012–1043 | Suppl. Mat. Table A, 5 atm series |
| x40001060 | 4 (3) | 0.15 | N_2_/CO_2_ | 0.1 | 2.4–3.0 | 992–1017 | Suppl. Mat. Table A, 3 atm series, **first data point excluded** |

**Table S9** Laminar flame speed measurements of syngas−oxygen mixtures

| **ID [.xml]** | **Type** | ***N*_i_** | ***σ* /  cm s^–1^** | **Diluents** | ***φ*** | ***p* / atm** | ***T* / K** | **Comment** |
| --- | --- | --- | --- | --- | --- | --- | --- | --- |
| **Burke et al. (2010)** [[35](#_ENREF_35)] | | | | | | | | |
| x20001001 | OPF | 6 | 6.52 | Ar | 2.5 | 1–25 | 295 | Suppl. Mat. Table A1, Fig. 10, full triangle, H_2_/CO=50/50 |
| x20001003 | OPF | 6 | 9.23 | CO_2_ | 2.5 | 1–25 | 295 | Suppl. Mat. Table A1, Fig. 12, full red diamond, *T_f_*=1500 K, **H_2_/O_2_/CO_2_ system** |
| x20001004 | OPF | 6 | 16.17 | CO_2_ | 2.5 | 1–25 | 295 | Suppl. Mat. Table A1, Fig. 12, full black square, *T_f_*=1600 K, **H_2_/O_2_/CO_2_ system** |
| x20001005 | OPF | 6 | 15.33 | CO_2_ | 2.5 | 1–25 | 295 | Suppl. Mat. Table A1, Fig. 12, full black circle, *T_f_*=1700 K, **H_2_/O_2_/CO_2_ system** |
| x20001006 | OPF | 6 | 27.48 | CO_2_ | 2.5 | 1–25 | 295 | Suppl. Mat. Table A1, Fig. 12, full blue triangle, *T_f_*=1800 K, **H_2_/O_2_/CO_2_ system** |
| **Das et al. (2011)** [[50](#_ENREF_50)] | | | | | | | | |
| x20001007 | CTF | 5 | 2.00 | N_2_/H_2_O | 0.6 | 1 | 323 | Suppl. Mat. Table, Fig. 4, open circle, H_2_/CO=0.053, H_2_O addition, in air |
| x20001008 | CTF | 5 | 2.01 | N_2_/H_2_O | 0.7 | 1 | 323 | Suppl. Mat. Table, Fig. 4, open square, H_2_/CO=0.053, H_2_O addition, in air |
| x20001009 | CTF | 5 | 2.00 | N_2_/H_2_O | 0.8 | 1 | 323 | Suppl. Mat. Table, Fig. 4, open diamond, H_2_/CO=0.053, H_2_O addition, in air |
| x20001010 | CTF | 5 | 2.00 | N_2_/H_2_O | 0.9 | 1 | 323 | Suppl. Mat. Table, Fig. 4, open triangle, H_2_/CO=0.053, H_2_O addition, in air |
| x20001013 | CTF | 5 | 2.00 | N_2_/H_2_O | 0.6 | 1 | 323 | Suppl. Mat. Table, Fig. 6, open diamond, H_2_/CO=1, H_2_O addition, in air |
| x20001014 | CTF | 5 | 2.00 | N_2_/H_2_O | 0.4 | 1 | 323 | Suppl. Mat. Table, Fig. 6, open circle, H_2_/CO=1, H_2_O addition, in air |
| x20001015 | CTF | 5 | 2.00 | N_2_/H_2_O | 0.5 | 1 | 323 | Suppl. Mat. Table, Fig. 6, open square, H_2_/CO=1, H_2_O addition, in air |
| x20001016 | CTF | 5 | 2.00 | N_2_/H_2_O | 0.6 | 1 | 323 | Suppl. Mat. Table, Fig. 7, open square, H_2_/CO=0.111, H_2_O addition, in air |
| x20001017 | CTF | 5 | 2.00 | N_2_/H_2_O | 0.6 | 1 | 323 | Suppl. Mat. Table, Fig. 7, open triangle, H_2_/CO=0.176, H_2_O addition, in air |
| x20001018 | CTF | 5 | 2.00 | N_2_/H_2_O | 0.6 | 1 | 323 | Suppl. Mat. Table, Fig. 7, full circle, H_2_/CO=0.250, H_2_O addition, in air |
| **Sun et al. (2007)** [[51](#_ENREF_51)] | | | | | | | | |
| x20001022 | OPF | 11 | 5.21 | N_2_ | 0.6–4.0 | 1 | 298 | Fig. 1, top, red circle,H_2_/CO=50/50, in air |
| x20001023 | OPF | 12 | 2.69 | N_2_ | 0.6–4.5 | 1 | 298 | Fig. 1, top, orange triangle, H_2_/CO=25/75, in air |
| x20001024 | OPF | 12 | 2.00 | N_2_ | 0.6–4.5 | 1 | 298 | Fig. 1, top, yellow square, H_2_/CO=5/95, in air |
| x20001025 | OPF | 10 | 3.33 | He | 0.8–3.5 | 5 | 298 | Fig. 2, top, red circle, H_2_/CO=50/50 |
| x20001026 | OPF | 12 | 2.70 | He | 0.6–4.0 | 10 | 298 | Fig. 2, top, yellow triangle, H_2_/CO=50/50 |
| x20001027 | OPF | 10 | 3.87 | He | 0.8–3.5 | 5 | 298 | Fig. 2, middle, red circle, H_2_/CO=25/75 |
| x20001028 | OPF | 12 | 2.10 | He | 0.8–4.0 | 10 | 298 | Fig. 2, middle, orange triangle, H_2_/CO=25/75 |
| x20001029 | OPF | 9 | 2.00 | He | 0.5–3.5 | 20 | 298 | Fig. 2, middle, yellow square, H_2_/CO=25/75 |
| x20001030 | OPF | 8 | 2.00 | He | 1.2–3.5 | 5 | 298 | Fig. 2, bottom, redcircle,H_2_/CO=5/95 |
| x20001031 | OPF | 10 | 2.26 | He | 0.8–3.5 | 10 | 298 | Fig. 2, bottom, orange triangle, H_2_/CO=5/95 |
| x20001032 | OPF | 9 | 2.00 | He | 1.0–3.5 | 20 | 298 | Fig. 2, bottom, yellow square, H_2_/CO=5/95 |
| x20001033 | OPF | 7 | 2.00 | He | 1.2–3.0 | 40 | 298 | Fig. 2, bottom, green diamond, H_2_/CO=5/95 |
| x20001137 | OPF | 9 | 2.00 | N_2_ | 0.8–4.2 | 1 | 298 | Fig. 1, top, green diamond, H_2_/CO=1/99, in air |
| x20001138 | OPF | 10 | 3.17 | N_2_ | 0.6–4.0 | 2 | 298 | Fig. 1, bottom, red circle, H_2_/CO= 50/50, in air |
| x20001139 | OPF | 12 | 2.11 | N_2_ | 0.6–4.5 | 2 | 298 | Fig. 1, bottom, orange circle, H_2_/CO=25/75, in air |
| x20001140 | OPF | 12 | 2.00 | N_2_ | 0.6–4.5 | 2 | 298 | Fig. 1, bottom, yellow triangle, H_2_/CO=5/95, in air |

| **Natarajan et al. (2009)** [[52](#_ENREF_52)] | | | | | | | | |
| --- | --- | --- | --- | --- | --- | --- | --- | --- |
| x20001034 | FCM | 9 | 3.12 | He | 0.6–1.2 | 10 | 300 | Fig. 2, open red triangle, H_2_/CO=50/50 |
| x20001035 | FCM | 12 | 2.00 | N_2_ | 0.6–1.0 | 1 | 300 | Fig. 3, open blue diamond, H_2_/CO=50/50, in air |
| x20001036 | FCM | 10 | 4.18 | N_2_ | 0.6–1.0 | 1 | 400 | Fig. 3, open red triangle, H_2_/CO=50/50, in air |
| x20001037 | FCM | 7 | 9.28 | N_2_ | 0.6–1.0 | 1 | 500 | Fig. 3, open green square, H_2_/CO=50/50, in air |
| x20001038 | FCM | 10 | 6.52 | N_2_ | 0.6–1.0 | 1 | 600 | Fig. 3, open brown circle, H_2_/CO=50/50, in air |
| x20001040 | FCM | 13 | 3.76 | He | 0.6 | 15 | 600 | Fig. 4, open triangle, varying H_2_/CO |
| x20001041 | FCM | 6 | 2.33 | He | 0.8 | 15 | 300 | Fig. 5, open diamond, varying H_2_/CO |
| x20001042 | FCM | 5 | 2.34 | He/CO_2_ | 0.75 | 15 | 600 | Fig. 8, open triangle, varying H_2_/CO,  40% CO_2_ dilution |
| **Prathap et al. (2008)** [[53](#_ENREF_53)] | | | | | | | | |
| x20001043 | OPF | 9 | 3.79 | N_2_ | 0.6–3.5 | 0.99 | 303 | Table 1, H_2_/CO/N_2_=50/50/0, in air |
| x20001044 | OPF | 8 | 2.97 | N_2_ | 0.6–3.0 | 0.99 | 303 | Table 2, H_2_/CO/N_2_=40/40/20, in air |
| x20001045 | OPF | 7 | 3.11 | N_2_ | 0.6–2.5 | 0.99 | 303 | Table 3, H_2_/CO/N_2_=30/30/40, in air |
| x20001046 | OPF | 7 | 2.00 | N_2_ | 0.6–1.8 | 0.99 | 303 | Table 4, H_2_/CO/N_2_=20/20/60, in air |
| **Bouvet et al. (2007)** [[54](#_ENREF_54)] | | | | | | | | |
| x20001049 | FCM | 5 | 2.00 | N_2_ | 0.7–0.9 | 1 | 298 | Fig. 7, pink square, H_2_/CO=70/30, in air |
| x20001050 | FCM | 7 | 2.00 | N_2_ | 0.7–1.05 | 1 | 298 | Fig. 7, red triangle, H_2_/CO=60/40, in air |
| x20001051 | FCM | 9 | 2.00 | N_2_ | 0.6–1.0 | 1 | 298 | Fig. 7, dark blue diamond, H_2_/CO=50/50, in air |
| x20001052 | FCM | 8 | 2.00 | N_2_ | 0.65–1.05 | 1 | 298 | Fig. 7, yellow diamond, H_2_/CO=40/60, in air |
| x20001053 | FCM | 8 | 2.00 | N_2_ | 0.65–1.1 | 1 | 298 | Fig. 7, black circle, H_2_/CO=30/70, in air |
| x20001054 | FCM | 8 | 2.00 | N_2_ | 0.65–1.2 | 1 | 298 | Fig. 7, green triangle, H_2_/CO=20/80, in air |
| x20001055 | FCM | 9 | 2.84 | N_2_ | 0.6–1.2 | 1 | 298 | Fig. 7, blue circle, H_2_/CO=10/90, in air |
| x20001056 | FCM | 4 | 2.00 | N_2_ | 0.6–0.7 | 1 | 298 | Fig. 8, dark blue diamond, H_2_/CO=10/90, in air |
| x20001057 | FCM | 7 | 2.00 | N_2_ | 0.6–0.85 | 1 | 298 | Fig. 8, pink square, H_2_/CO=5/95, in air |
| x20001058 | FCM | 8 | 2.00 | N_2_ | 0.6–0.95 | 1 | 298 | Fig. 8, red triangle, H_2_/CO=3/97, in air |
| x20001059 | FCM | 5 | 2.00 | N_2_ | 0.9–1.15 | 1 | 298 | Fig. 8, green circle, H_2_/CO=1/99, in air |
| **McLean et al. (1994)** [[55](#_ENREF_55)] | | | | | | | | |
| x20001060 | OPF | 11 | 2.25 | N_2_ | 0.6–4.4 | 1 | 300 | open circle, H_2_/CO=50/50, in air |
| x20001061 | OPF | 20 | 2.00 | N_2_ | 0.4–5.8 | 1 | 300 | open diamond, H_2_/CO=5/95, in air |
| **Burbano et al. (2011)** [[56](#_ENREF_56)] | | | | | | | | |
| x20001062 | FCM | 12 | 2.7 | N_2_ | 0.7–4.3 | 0.95 | 303 | Fig. 4, lower square, H_2_/CO=25/75, in air |
| x20001063 | FCM | 13 | 4.63 | N_2_ | 0.7–4.3 | 0.95 | 303 | Fig. 7, square, H_2_/CO=50/50, in air, 0% N_2_ dilution |
| x20001064 | FCM | 9 | 2.23 | N_2_ | 0.8–3.8 | 0.95 | 303 | Fig. 7, triangle, H_2_/CO=50/50, in air, 20% N_2_ dilution |
| x20001065 | FCM | 11 | 2.48 | N_2_ | 0.8–3.8 | 0.95 | 303 | Fig. 7, circle, H_2_/CO=50/50, in air, 40% N_2_ dilution |
| x20001066 | FCM | 6 | 7.01 | N_2_ | 1.2–2.8 | 0.95 | 303 | Fig. 7, diamond, H_2_/CO=50/50, in air, 60% N_2_ dilution |
| x20001067 | FCM | 11 | 2.42 | N_2_/CO_2_ | 0.8–4.3 | 0.95 | 303 | Fig. 8,triangle, H_2_/CO=50/50, in air, 10% CO_2_ dilution |
| x20001068 | FCM | 10 | 3.63 | N_2_/CO_2_ | 0.8–3.4 | 0.95 | 303 | Fig. 8, diamond, H_2_/CO=50/50, in air, 20% CO_2_ dilution |

| **Natarajan et al. (2007)** [[57](#_ENREF_57)] | | | | | | | | |
| --- | --- | --- | --- | --- | --- | --- | --- | --- |
| x20001069 | FCM | 12 | 2.00 | N_2_ | 0.6–1.0 | 1 | 300 | Fig. 5, dark blue square, H_2_/CO=50/50, in air, 0% CO_2_ dilution |
| x20001070 | FCM | 13 | 2.00 | N_2_/CO_2_ | 0.6–1.0 | 1 | 300 | Fig. 5, red triangle, H_2_/CO=50/50, in air, 20% CO_2_ dilution |
| x20001071 | FCM | 13 | 2.00 | N_2_ | 0.65–1.05 | 1 | 300 | Fig. 7, dark blue square, H_2_/CO=5/95, in air, 0% CO_2_ dilution |
| x20001072 | FCM | 8 | 2.00 | N_2_/CO_2_ | 0.7–1.0 | 1 | 300 | Fig. 7, red triangle, H_2_/CO=5/95, in air, 10% CO_2_ dilution |
| x20001074 | FCM | 14 | 2.00 | N_2_/CO_2_ | 0.6–1.0 | 1 | 300 | Fig. 8, red triangle, H_2_/CO=95/5, in air, 20% CO_2_ dilution |
| x20001075 | FCM | 10 | 2.56 | N_2_ | 0.5–0.8 | 1 | 700 | Fig. 12, blue star, H_2_/CO=5/95, in air |
| x20001076 | FCM | 10 | 2.06 | N_2_ | 0.55–0.9 | 1 | 600 | Fig. 12, brown circle, H_2_/CO=5/95, in air |
| x20001077 | FCM | 9 | 2.00 | N_2_ | 0.6–0.95 | 1 | 500 | Fig. 12, green square, H_2_/CO=5/95, in air |
| x20001078 | FCM | 10 | 2.00 | N_2_ | 0.6–1.0 | 1 | 400 | Fig. 12, red triangle, H_2_/CO=5/95, in air |
| x20001079 | FCM | 13 | 2.00 | N_2_ | 0.65–1.05 | 1 | 300 | Fig. 12, dark blue diamond, H_2_/CO=5/95, in air |
| x20001080 | FCM | 11 | 5.23 | N_2_/CO_2_ | 0.45–0.74 | 1 | 600 | Fig. 13, blue circle, H_2_/CO=5/95, in air, 20% CO_2_ dilution |
| x20001081 | FCM | 14 | 4.81 | N_2_/CO_2_ | 0.5–0.9 | 1 | 500 | Fig. 13, green square, H_2_/CO=5/95, in air, 20% CO_2_ dilution |
| x20001082 | FCM | 12 | 2.74 | N_2_/CO_2_ | 0.55–1.0 | 1 | 400 | Fig. 13, red triangle, H_2_/CO=5/95, in air, 20% CO_2_ dilution |
| x20001083 | FCM | 14 | 2.22 | N_2_/CO_2_ | 0.6–1.05 | 1 | 300 | Fig. 13, dark blue diamond, H_2_/CO=5/95, in air, 20% CO2 dilution |
| **Bouvet et al. (2011)** [[58](#_ENREF_58)] | | | | | | | | |
| x20001084 | OPF | 16 | 2.00 | N_2_ | 0.4–5.0 | 1 | 295 | Fig. 5, yellow square, H_2_/CO=5/95, in air |
| x20001085 | OPF | 14 | 2.00 | N_2_ | 0.4–5.0 | 1 | 295 | Fig. 6, yellow square, H_2_/CO=10/90, in air |
| **Hassan et al. (1997)** [[59](#_ENREF_59)] | | | | | | | | |
| x20001088 | OPF | 13 | 2.00 | N_2_ | 0.6–5.0 | 1 | 298 | Table 1, H_2_/CO=5/95, in air |
| x20001089 | OPF | 13 | 3.66 | N_2_ | 0.6–5.0 | 1 | 298 | Table 1, H_2_/CO=10/90, in air |
| x20001091 | OPF | 12 | 4.80 | N_2_ | 0.6–4.5 | 1 | 298 | Table 1, H_2_/CO=50/50, in air |
| x20001105 | OPF | 11 | 2.18 | N_2_/CO_2_ | 0.6–5.0 | 1 | 298 | Table 1, H_2_/CO=3/97, in air |
| x20001106 | OPF | 11 | 2.00 | N_2_ | 0.8–4.5 | 0.5 | 298 | Table 2, H_2_/CO=5/95, p=0.5 atm, in air |
| x20001107 | OPF | 12 | 2.00 | N_2_ | 0.6–5.0 | 2 | 298 | Table 2, H_2_/CO=5/95, p=2 atm, in air |
| x20001108 | OPF | 11 | 2.00 | N_2_ | 0.6–5.0 | 4 | 298 | Table 2, H_2_/CO=5/95, p=4 atm, in air |
| **Ratna Kishore et al. (2011)** [[60](#_ENREF_60)] | | | | | | | | |
| x20001092 | HFM | 5 | 2.00 | N_2_ | 0.8–1.6 | 1 | 308 | Fig. 2, open square, H_2_/CO/CO_2_=5/95/0, in air |
| x20001093 | HFM | 5 | 2.00 | N_2_/CO_2_ | 1.0–2.0 | 1 | 303 | Fig. 2, half-filled open square, H_2_/CO/CO_2_=5/45/50, in air |
| x20001094 | HFM | 7 | 2.00 | N_2_/CO_2_ | 0.8–2.0 | 1 | 303 | Fig. 3, open circle, H_2_/CO/CO_2_=40/10/50, in air |
| x20001095 | HFM | 7 | 2.00 | N_2_/CO_2_ | 0.8–2.0 | 1 | 303 | Fig. 3, open square, H_2_/CO/CO_2_=25/25/50, in air |
| x20001096 | HFM | 7 | 2.00 | N_2_/CO_2_ | 0.8–2.0 | 1 | 303 | Fig. 3, open diamond, H_2_/CO/CO_2_=10/40/50, in air |
| x20001097 | HFM | 6 | 2.00 | N_2_/CO_2_ | 0.8–1.8 | 1 | 303 | Fig. 8, open circle, H_2_/CO/CO_2_=32/8/60, in air |
| x20001098 | HFM | 6 | 2.00 | N_2_/CO_2_ | 0.8–1.8 | 1 | 303 | Fig. 8, open reverse triangle, H_2_/CO/CO_2_=20/20/60, in air |
| x20001099 | HFM | 6 | 2.00 | N_2_/CO_2_ | 0.8–1.8 | 1 | 303 | Fig. 8, open square, H_2_/CO/CO_2_=8/32/60, in air |
| x20001100 | HFM | 1 | 2.00 | N_2_/CO_2_ | 0.8 | 1 | 303 | Fig. 9, open circle, H_2_/CO/CO_2_=48/12/40, in air |
| x20001101 | HFM | 7 | 2.00 | N_2_/CO_2_ | 0.8–2.0 | 1 | 303 | Fig. 9, open reverse triangle, H_2_/CO/CO_2_=30/30/40, in air |
| x20001102 | HFM | 7 | 2.00 | N_2_/CO_2_ | 0.8–2.0 | 1 | 303 | Fig. 9, open square, H_2_/CO/CO_2_=12/48/40, in air |
| **Konnov et al. (2002)** [[61](#_ENREF_61)] | | | | | | | | |
| x20001103 | HFM | 14 | 2.00 | N_2_/CO_2_ | 0.8–2.3 | 1 | 298 | Fig. 1, cross, H_2_/CO/CO_2_=5.05/44.75/50.2, in air |
| **Huang et al. (2004)** [[62](#_ENREF_62)] | | | | | | | | |
| x20001109 | CTF | 8 | 2.00 | N_2_ | 0.7–1.4 | 1 | 298 | Fig. 13, open circle, H_2_/CO/N_2_=28/25/47, in air |

| **Vagelopoulos and Egolfopoulos** (**1994)** [[63](#_ENREF_63)] | | | | | | | | |
| --- | --- | --- | --- | --- | --- | --- | --- | --- |
| x20001110 | CTF | 10 | 2.00 | N_2_ | 0.6 | 1 | 298 | Fig. 1, (*X*_CO_+*X*_H2_)=0.20, H_2_/CO ratio varied, in air |
| x20001111 | CTF | 7 | 2.00 | N_2_ | 0.49 | 1 | 298 | Fig. 1, (*X*_CO_+*X*_H2_)=0.17, H_2_/CO ratio varied, in air |
| x20001112 | CTF | 4 | 2.00 | N_2_ | 0.39 | 1 | 298 | Fig. 1, (*X*_CO_+*X*_H2_)=0.14, H_2_/CO ratio varied, in air |
| **Jahn** [[64](#_ENREF_64)] (reprinted by Lewis and von Elbe (1961) [[65](#_ENREF_65)]) | | | | | | | | |
| x20001113 | FCM | 22 | 2.00 | N_2_/H_2_O | 0.3–6.7 | 1 | 298 | Fig. 190, H_2_/CO/O_2_/N_2_/H_2_O, *f*=0.985 |
| x20001114 | FCM | 16 | 2.00 | N_2_/H_2_O | 0.4–3.4 | 1 | 298 | Fig. 190, H_2_/CO/O_2_/N_2_/H_2_O, *f*=0.80 |
| x20001115 | FCM | 12 | 2.00 | N_2_/H_2_O | 0.3–4.0 | 1 | 298 | Fig. 190, H_2_/CO/O_2_/N_2_/H_2_O, *f*=0.60 |
| x20001116 | FCM | 18 | 2.00 | N_2_/H_2_O | 0.8–5.6 | 1 | 298 | Fig. 190, H_2_/CO/O_2_/N_2_/H_2_O, *f*=0.40 |
| x20001117 | FCM | 16 | 2.00 | N_2_/H_2_O | 1.1–6.6 | 1 | 298 | Fig. 190, H_2_/CO/O_2_/N_2_/H_2_O, *f*=0.30 |
| x20001118 | FCM | 12 | 2.00 | N_2_/H_2_O | 1.4–4.2 | 1 | 298 | Fig. 190, H_2_/CO/O_2_/N_2_/H_2_O,f=0.21, in air |
| x20001119 | FCM | 10 | 2.00 | N_2_/H_2_O | 1.6–4.5 | 1 | 298 | Fig. 190, H_2_/CO/O_2_/N_2_/H_2_O,*f*=0.17 |
| x20001120 | FCM | 7 | 2.00 | N_2_/H_2_O | 2.0–3.4 | 1 | 298 | Fig. 190, H_2_/CO/O_2_/N_2_/H_2_O, *f*=0.13 |
| x20001121 | FCM | 17 | 2.00 | N_2_/H_2_O | 0.3–4.3 | 1 | 298 | Fig. 191, CO/O_2_/N_2_/H_2_O, *f*=0.985 |
| x20001122 | FCM | 12 | 2.00 | N_2_/H_2_O | 1.3–4.0 | 1 | 298 | Fig. 191, CO/O_2_/N_2_/H_2_O, *f*=0.21, in air |
| x20001123 | FCM | 20 | 2.15 | H_2_O | 0.3–6.8 | 1 | 298 | Fig. 192, H_2_/CO/O_2_/CO_2_/H_2_O, *f*=1.00 |
| x20001124 | FCM | 16 | 2.00 | CO_2_/H_2_O | 0.5–2.8 | 1 | 298 | Fig. 192 H_2_/CO/O_2_/CO_2_/H_2_O, *f*=0.80 |
| x20001125 | FCM | 21 | 2.00 | CO_2_/H_2_O | 0.7–5.9 | 1 | 298 | Fig. 192, H_2_/CO/O_2_/CO_2_/H_2_O, *f*=0.60 |
| x20001126 | FCM | 17 | 2.00 | CO_2_/H_2_O | 1.0–5.8 | 1 | 298 | Fig. 192, H_2_/CO/O_2_/CO_2_/H_2_O, *f*=0.40 |
| x20001127 | FCM | 17 | 2.00 | CO_2_/H_2_O | 1.1–5.7 | 1 | 298 | Fig. 192, H_2_/CO/O_2_/CO_2_/H_2_O, *f*=0.30 |
| x20001128 | FCM | 10 | 2.00 | CO_2_/H_2_O | 1.6–4.5 | 1 | 298 | Fig. 192, H_2_/CO/O_2_/CO_2_/H_2_O, *f*=0.21 |
| **Singh *et al.* (2012)** [[66](#_ENREF_66)] | | | | | | | | |
| x20001129 | OPF | 5 | 13.11 | N_2_ | 0.6–3.0 | 1 | 298 | Fig. 2, H_2_/CO=75/25, in air |
| x20001130 | OPF | 4 | 2.00 | N_2_ | 0.8–3.0 | 1 | 298 | Fig. 2, H_2_/CO=25/75, in air |
| x20001131 | OPF | 5 | 3.11 | N_2_ | 0.6–3.0 | 1 | 298 | Fig. 2, H_2_/CO=5/95, in air |
| x20001132 | OPF | 5 | 20.24 | N_2_ | 0.6–3.0 | 1 | 500 | Fig. 3, H_2_/CO=50/50, T=500 K, in air |
| x20001133 | OPF | 5 | 19.37 | N_2_ | 0.6–3.0 | 1 | 400 | Fig. 3, H_2_/CO=50/50, T=400 K, in air |
| x20001134 | OPF | 5 | 7.36 | N_2_ | 0.6–3.0 | 1 | 298 | Fig. 3, H_2_/CO=50/50, T=298 K, in air |
| x20001135 | OPF | 4 | 2.69 | N_2_/H_2_O | 1.0 | 1 | 400 | Fig. 5, H_2_/CO=50/50, varying H_2_O addition, in air |
| x20001136 | OPF | 5 | 2.30 | N_2_/H_2_O | 1.0 | 1 | 400 | Fig. 5, H_2_/CO=5/95, varying H_2_O addition, in air |
| **Scholte and Vaags (1959)** [[67](#_ENREF_67)] | | | | | | | | |
| x20001142 | FCM | 7 | 3.13 | N_2_ | 1.28–4.42 | 1 | 298 | Data in: [[68](#_ENREF_68)], Fig. 3, CO/(CO+H_2_)=0.303, in air |
| x20001143 | FCM | 7 | 3.71 | N_2_ | 1.28–4.42 | 1 | 298 | Data in: [[68](#_ENREF_68)], Fig. 3, CO/(CO+H_2_)=0.567, in air |
| x20001144 | FCM | 7 | 4.86 | N_2_ | 1.28–4.42 | 1 | 298 | Data in: [[68](#_ENREF_68)], Fig. 3, CO/(CO+H_2_)=0.759, in air |
| x20001145 | FCM | 7 | 2.00 | N_2_ | 1.28–4.42 | 1 | 298 | Data in: [[68](#_ENREF_68)], Fig. 3, CO/(CO+H_2_)=0.895, in air |
| x20001146 | FCM | 7 | 2.08 | N_2_ | 1.28–4.42 | 1 | 298 | Data in: [[68](#_ENREF_68)], Fig. 3, CO/(CO+H_2_)=0.97, in air |
| x20001147 | FCM | 7 | 2.00 | N_2_ | 1.28–4.42 | 1 | 298 | Data in: [[68](#_ENREF_68)], Fig. 3, CO/(CO+H_2_)=0.9945, in air |
| **Krejci et al. (2012)** [[69](#_ENREF_69)] | | | | | | | | |
| x20001151 | OPF | 19 | 2.00 | N_2_/H_2_O | 0.6–5.0 | 1 | 320–325 | Table A1, H_2_/CO=5/95, 7.5% H_2_O addition, in air |
| x20001152 | OPF | 14 | 5.66 | He | 0.55–4.8 | 5 | 322–325 | Table A1, H_2_/CO=50/50, no H_2_O addition |
| x20001153 | OPF | 11 | 2.00 | He/H_2_O | 0.65–4.2 | 5 | 373–374 | Table A2, H_2_/CO=5/95, 15% H_2_O addition |
| x20001154 | OPF | 12 | 4.11 | He/H_2_O | 0.6–3.4 | 10 | 372–374 | Table A2, H_2_/CO=50/50, 7.5% H_2_O addition |
| x20001155 | OPF | 18 | 2.29 | N_2_/H_2_O | 0.6–5.0 | 1 | 422–423 | Table A3, H_2_/CO=50/50, 15% H_2_O addition, in air |
| x20001156 | OPF | 13 | 4.95 | He | 0.6–5.0 | 10 | 422–424 | Table A3, H_2_/CO=5/95, no H_2_O addition |

| **Zhang et al. (2009)** [[70](#_ENREF_70)] | | | | | | | | |
| --- | --- | --- | --- | --- | --- | --- | --- | --- |
| x20001157 | OPF | 12 | 5.65 | N_2_ | 0.6–4.0 | 0.99 | 310 | Fig. 18, full circle, H_2_/CO=66.7/33.3, in air |
| x20001158 | OPF | 11 | 5.80 | N_2_ | 0.6–3.5 | 0.99 | 310 | Fig. 20a, open circle, H_2_/CO=66.7/33.3,  N_2_ dilution, dilution ratio 0.1 |
| x20001161 | OPF | 4 | 6.99 | N_2_ | 0.8–1.4 | 0.99 | 310 | Fig. 20a, open diamond, H_2_/CO= 66.7/33.3,  N_2_ dilution, dilution ratio 0.4 |
| x20001162 | OPF | 11 | 7.00 | N_2_/CO_2_ | 0.6–3.5 | 0.99 | 310 | Fig. 20b, open circle, H_2_/CO=66.7/33.3,  CO_2_ dilution, dilution ratio 0.1, in air |
| x20001164 | OPF | 5 | 8.73 | N_2_/CO_2_ | 0.8–1.6 | 0.99 | 310 | Fig. 20b, open reverse triangle, H_2_/CO= 66.7/33.3, CO_2_ dilution, dilution ratio 0.3, in air |
| x20001165 | OPF | 3 | 7.97 | N_2_/CO_2_ | 1.0–1.4 | 0.99 | 310 | Fig. 20b, open diamond, H2/CO=66.7/33.3, CO_2_ dilution, dilution ratio 0.4, in air |
| x20001192 | OPF | 12 | 5.51 | N_2_ | 0.6–4.0 | 0.99 | 360 | Fig. 17a, open circle, H_2_/CO=66.7/33.3, in air |
| x20001193 | OPF | 12 | 7.99 | N_2_ | 0.6–4.0 | 0.99 | 410 | Fig. 17a, open triangle, H_2_/CO=66.7/33.3, in air |
| x20001194 | OPF | 5 | 13.67 | N_2_ | 2.0–4.0 | 2.47 | 310 | Fig. 17b, open circle, H_2_/CO=66.7/33.3, in air |
| x20001195 | OPF | 3 | 10.04 | N_2_ | 3.0–4.0 | 4.93 | 310 | Fig. 17b, open triangle, H_2_/CO=66.7/33.3, in air |
| **Dong et al. (2009)** [[40](#_ENREF_40)] | | | | | | | | |
| x20001167 | FCM | 15 | 2.15 | N_2_ | 0.7–2.1 | 1 | 293 | Fig. 6, red diamond, H_2_/(CO+H_2_)=10%, in air |
| x20001168 | FCM | 16 | 2.74 | N_2_ | 0.6–2.1 | 1 | 293 | Fig. 6, blue triangle, H_2_/(CO+H_2_)=20%, in air |
| x20001169 | FCM | 16 | 5.14 | N_2_ | 0.6–2.1 | 1 | 293 | Fig. 6, sea green triangle, H_2_/(CO+H_2_)=30%, in air |
| x20001170 | FCM | 17 | 4.81 | N_2_ | 0.5–2.1 | 1 | 293 | Fig. 6, pink triangle, H_2_/(CO+H_2_)=40%, in air |
| x20001171 | FCM | 17 | 2.82 | N_2_ | 0.5–2.1 | 1 | 293 | Fig. 6, olive triangle, H_2_/(CO+H_2_)=50%, in air |
| x20001172 | FCM | 17 | 4.27 | N_2_ | 0.5–2.1 | 1 | 293 | Fig. 6, dark blue diamond, H_2_/(CO+H_2_)=60%, in air |
| x20001173 | FCM | 17 | 5.78 | N_2_ | 0.5–2.1 | 1 | 293 | Fig. 6, dark red circle, H_2_/(CO+H_2_)=70%, in air |
| x20001174 | FCM | 17 | 3.52 | N_2_ | 0.5–2.1 | 1 | 293 | Fig. 6, magenta circle, H_2_/(CO+H_2_)=80%, in air |
| x20001175 | FCM | 17 | 6.65 | N_2_ | 0.5–2.1 | 1 | 293 | Fig. 6, green asterisk, H_2_/(CO+H_2_)=90%, in air |
| **Burke et al. (2007)** [[71](#_ENREF_71)] | | | | | | | | |
| x20001176 | OPF | 9 | 3.17 | N_2_ | 0.6–4.0 | 1 | 298 | Fig. 6, square, CO/H_2_=50/50, in air |
| x20001177 | OPF | 8 | 2.00 | He | 0.8–3.0 | 10 | 298 | Fig. 7a, triangle, CO/H_2_=75/25 |
| x20001178 | OPF | 9 | 2.28 | He | 0.8–3.0 | 20 | 298 | Fig. 7a, diamond, CO/H_2_=75/25 |
| x20001179 | OPF | 5 | 2.00 | He/CO_2_ | 1 | 10 | 298 | Fig. 7b, triangle, CO/H_2_=75/25, *X*_CO2_ varied |
| **Santner et al. (2013)** [[41](#_ENREF_41)] | | | | | | | | |
| x20001180 | OPF | 3 | 12.22 | He | 0.85 | 1–10 | 393 | Fig. 2 bottom, open green circle, calculated from mass burning rate, CO/H_2_=50/50, no H_2_O dilution |
| x20001181 | OPF | 5 | 2.82 | He/H_2_O | 0.85 | 1–10 | 393 | Fig. 2 bottom, open blue diamond, calculated from mass burning rate, CO/H_2_=50/50, 5% H_2_O dilution |
| x20001182 | OPF | 4 | 7.19 | He/H_2_O | 0.85 | 1–6 | 393 | Fig. 2 bottom, open orange triangle, calculated from mass burning rate, CO/H_2_=50/50, 15% H_2_O dilution |
| **Lohöfener et al. (2012)** [[72](#_ENREF_72)] | | | | | | | | |
| x20001183 | HFM | 12 | 2.00 | N_2_ | 0.7–1.8 | 0.96 | 302 | Fig. p. 11, red circle, H_2_/CO=1, 60% N_2_ dilution |
| x20001184 | HFM | 11 | 2.00 | N_2_ | 0.9–1.9 | 0.96 | 298 | Fig. p. 12, blue circle, H_2_/CO=2,  70% N_2_ dilution |
| x20001188 | HFM | 11 | 2.00 | N_2_/CO_2_ | 0.7–2.0 | 0.96 | 300 | Fig. p. 14, red circle, H_2_/CO=2,  70% dilution (60% N_2_, 10% CO_2_) |
| x20001189 | HFM | 9 | 2.00 | N_2_/CO_2_ | 0.7–1.5 | 0.97 | 300 | Fig. p. 14, green triangle, H_2_/CO=2,  air, 60% dilution (50% N_2_, 10% CO_2_) |
| **Weng et al. (2013)** [[73](#_ENREF_73)] | | | | | | | | |
| x20001196 | HFM | 17 | 2.00 | N_2_ | 0.6–5.6 | 1 | 298 | Fig. 5, full circle, in air |
| x20001197 | HFM | 9 | 2.00 | N_2_ | 0.4–0.8 | 1 | 298 | Fig. 6, full circle, in air |
| x20001198 | HFM | 17 | 2.00 | N_2_ | 0.7–2.3 | 1 | 298 | Fig. 7, full circle, in air, 60% N_2_ dilution |

| **Goswami et al. (2014)** [[74](#_ENREF_74)] | | | | | | | | |
| --- | --- | --- | --- | --- | --- | --- | --- | --- |
| x20001199 | HFM | 11 | 2.00 | N_2_ | 0.6–0.8 | 1 | 298 | Suppl. Mat. Table 1, H_2_/CO=50/50, in air |
| x20001200 | HFM | 9 | 2.00 | He | 0.7 | 1–5 | 298 | Suppl. Mat. Table 1, H_2_/CO=50/50, He/O_2_=90/10 |
| x20001201 | HFM | 7 | 2.00 | N_2_ | 1 | 1–4 | 298 | Suppl. Mat. Table 1, H_2_/CO=50/50, N_2_/O_2_=90/10 |
| x20001202 | HFM | 6 | 2.00 | N_2_ | 0.7 | 1–3.5 | 298 | Suppl. Mat. Table 1, H_2_/CO=50/50, N_2_/O_2_=85/15 |
| x20001203 | HFM | 9 | 2.00 | He | 0.6 | 1–9 | 298 | Suppl. Mat. Table 1, H_2_/CO=50/50, He/O_2_=87.5/12.5 |
| x20001204 | HFM | 7 | 2.00 | N_2_ | 0.7 | 1–4 | 298 | Suppl. Mat. Table 1, H_2_/CO=85/15, N_2_/O_2_=85/15 |
| **Kim et al. (2010)** [[75](#_ENREF_75)] | | | | | | | | |
| x20001206 | OPF | 8 | 2.18 | N_2_ | 0.8–3.0 | 1 | 298 | Fig. 2a, H_2_/CO=50/50, in air |
| x20001207 | OPF | 10 | 2.86 | N_2_/CO_2_ | 0.8–5.0 | 1 | 298 | Fig. 2b, H_2_/CO=50/50, 10% CO_2_ dilution, in air |
| **Wu et al. (2011)** [[76](#_ENREF_76)] | | | | | | | | |
| x20001208 | OPF | 9 | 2.00 | N_2_ | 1.40–1.59 | 1 | 293 | Fig. 8, *ϕ*_F_=0.70, parameter *R*_H_ varied, in air |
| x20001209 | OPF | 16 | 2.00 | N_2_ | 2 | 1 | 293 | Fig. 8, *ϕ*_F_=1.00, parameter *R*_H_ varied, in air |
| x20001210 | OPF | 10 | 2.00 | N_2_ | 2.76–3.19 | 1 | 293 | Fig. 8, *ϕ*_F_=1.60, parameter *R*_H_ varied, in air |
| x20001211 | OPF | 8 | 2.00 | He | 3.36 | 20 | 293 | Fig. 14, *ϕ*_F_=1.00, parameter *R*_H_ varied |
| **Voss et al. (2014)** [[77](#_ENREF_77)] | | | | | | | | |
| x20001212 | HFM | 10 | 2.00 | N_2_ | 0.6–1.5 | ≈0.96 | 298 | Fig. 9, black cross, H_2_/CO=33.3/66.7, 70% N_2_ dilution |
| x20001213 | HFM | 12 | 2.00 | N_2_ | 0.6–1.5 | ≈0.95 | 298 | Fig. 8, black cross, H_2_/CO=50/50, 70% N_2_ dilution |
| x20001214 | HFM | 10 | 2.00 | N_2_ | 0.6–1.5 | ≈0.95 | 298 | Fig. 9, blue cross, H_2_/CO=66.7/33.3, 70% N_2_ dilution |
| x20001215 | HFM | 9 | 2.00 | N_2_ | 0.7–1.5 | ≈0.96 | 298 | Fig. 8, blue cross, H_2_/CO=80/20, 70% N_2_ dilution |
| x20001216 | HFM | 9 | 2.00 | N_2_ | 1 | ≈0.95 | 298 | Fig. 6, cross, H_2_/CO=66.7/33.3, N_2_ dilution varied |
| x20001217 | HFM | 4 | 2.00 | N_2_ | 1 | ≈0.96 | 298 | Fig. 7, cross, H_2_/CO varied,70% N_2_ dilution |
| **Zhang et al. (2014)** [[78](#_ENREF_78)] | | | | | | | | |
| x20001218 | CTF | 9 | 2.00 | N_2_ | 0.4 | 1 | 298 | Fig. 4, full black square |
| x20001219 | CTF | 9 | 2.00 | N_2_ | 0.5 | 1 | 298 | Fig. 4, full red circle |
| x20001220 | CTF | 9 | 2.00 | N_2_ | 0.6 | 1 | 298 | Fig. 4, full blue triangle |
| x20001221 | CTF | 6 | 2.79 | N_2_ | 0.7 | 1 | 298 | Fig. 4, full green reverse triangle |
| **Li et al. (2015)** [[79](#_ENREF_79)] | | | | | | | | |
| x20001222 | OPF | 4 | 3.38 | CO_2_ | 2.5 | 5–25 | 353 | Table S2, Exp. No. 10a–d, **scatter error could not be determined. Average experimental error was chosen instead** |

Some measurements found in literature could not be modeled successfully due to convergence problems with the PREMIX solver, particularly at conditions where low ﬂame velocities occur ([[35](#_ENREF_35)] – Suppl. Mat. Table A1, Fig. 10, full diamond, H_2_/CO=10/90; [[39](#_ENREF_39)] – Fig. 3, Fig. 4, Fig. 6; [[67](#_ENREF_67)] – data in: [[68](#_ENREF_68)], Fig. 4; [[40](#_ENREF_40)] – Fig. 6, black square; [[72](#_ENREF_72)] – Fig. p. 13, Fig. p. 15). Some other measurements were not used, because all mechanisms showed a strong over-prediction with all mechanisms ([[52](#_ENREF_52)] – Fig. 3, blue star). From the two repeated measurements of [[61](#_ENREF_61)], only one was used (Fig. 1, cross, *x20001103.xml*). In total, these unconsidered measurements sum up to 153 data points (equal to 7.2% of all collected flame velocity data points) in 15 datasets from 7 publications.

**Table S10** Flow reactor concentration–time measurements of carbon monoxide−oxygen mixtures

| **ID [.xml]** | ***N*_i_** | ***σ*** | **Diluents** | ***φ*** | ***p* / atm** | ***T* / K** | **Comment** |
| --- | --- | --- | --- | --- | --- | --- | --- |
| **Mueller et al. (1999)** [[80](#_ENREF_80)] | | | | | | | |
| x30001002 | 8 (20) | 2.629E–4 (CO) | N_2_/H_2_O | 0.97 | 1 | 1038 | Fig. 1, open circle |
| x30001003 | 4 (16) | 1.544E–4 (CO) | N_2_/H_2_O | 1.01 | 2.4 | 1038 | Fig. 1, open square |
| x30001004 | 12 (21) | 6.023E–4 (CO) | N_2_/H_2_O | 1.01 | 3.5 | 1038 | Fig. 1, open triangle |
| x30001005 | 10 (15) | 9.900E–5 (CO) | N_2_/H_2_O | 1.01 | 9.6 | 1040 | Fig. 1, open reverse triangle |
| **Kim et al. (1994)** [[81](#_ENREF_81)] | | | | | | | |
| x30001006 | 8 (20) | 1.000E–4 (CO) | N_2_/H_2_O | 1 | 1 | 1040 | Fig. 4, open circle |
| x30001007 | 12 (21) | 6.105E–4 (CO) | N_2_/H_2_O | 1 | 3.46 | 1040 | Fig. 4, full circle |
| x30001008 | 13 (18) | 1.000E–4 (CO) | N_2_/H_2_O | 1 | 9.6 | 1040 | Fig. 6, open square |
| x30001009 | 13 (18) | 1.000E–4 (CO) | N_2_/H_2_O | 1 | 6.5 | 1040 | Fig. 6, full triangle |
| x30001010 | 4 (15) | 2.605E–4 (CO) | N_2_/H_2_O | 1 | 2.45 | 1040 | Fig. 6, full circle |
| x30001011 | 11 (18) | 1.000E–4 (CO) | N_2_/H_2_O | 1 | 6.5 | 1030 | Fig. 9, open circle |
| **Linteris et al. (1991)** [[21](#_ENREF_21)] | | | | | | | |
| x30001026 | 8 (18)  8 (18)  8 (18) | 2.282E–4 (CO)  2.610E–4 (CO_2_)  1.046E–4 (O_2_) | N_2_/H_2_O | 0.46 | 1 | 1115 | Fig. 5 |
| x30001027 | 12 (17) | 1.036E–6 (OH) | N_2_/H_2_O | 0.47 | 1 | 989 | Fig. 7, cross and open square symbols, **time-shifting using the CO profile from x30001023.xml** |
| x30001028 | 7 (15) | 2.301E–6 (OH) | N_2_/H_2_O | 0.46 | 1 | 1115 | Fig. 8, cross and open square symbols, **time-shifting using the CO profile from x30001026.xml** |
| **Yetter et al. (1991)** [[21](#_ENREF_21)] | | | | | | | |
| x30001030 | 13 (15) | 1.621E–6 (CO) | N_2_/H_2_O | 0.004 | 1 | 1074 | Fig. 6, up triangle |
| x30001031 | 4 (15) | 5.152E–6 (CO) | N_2_/H_2_O | 0.013 | 1 | 1132 | Fig. 6, square |
| x30001032 | 6 (15) | 1.489E–6 (CO) | N_2_/H_2_O | 0.004 | 1 | 1130 | Fig. 6, circle |
| x30001033 | 7 (11) | 4.686E–7 (CO) | N_2_/H_2_O | 0.001 | 1 | 1138 | Fig. 6, diamond |
| x30001036 | 14 (14) | 5.286E–5 (CO) | N_2_/H_2_O | 1.696 | 1 | 1042 | Fig. 8, asterisk |
| x30001040 | 15 (17) | 1.251E–4 (CO) | N_2_/H_2_O | 0.024 | 1 | 1041 | Fig. 9, down triangle |
| x30001046 | 4 (15) | 9.058E–5 (CO) | N_2_/H_2_O | 1.024 | 1 | 943 | Fig. 10, square |
| x30001049 | 17 (18)  17 (18)  17 (18) | 9.200E–5 (CO)  8.125E–5 (CO_2_)  4.050E–5 (O_2_) | N_2_/H_2_O | 1.438 | 1 | 1034 | Data in: [[82](#_ENREF_82)], Fig. 10b |

**Table S11.** Flow reactor outlet concentration measurements of carbon monoxide−oxygen mixtures

| **ID [.xml]** | ***N*_i_** | ***σ*** | **Diluents** | ***φ*** | ***p* / atm** | ***T* / K** | **Comment** |
| --- | --- | --- | --- | --- | --- | --- | --- |
| **Glarborg et al. (1996)** [[83](#_ENREF_83)] | | | | | | | |
| x30001014 | 12 | 4.103E–5 (CO) | N_2_/H_2_O | 0.0061 | 1.05 | 910–1200 | Fig. 6 top, set 2 (1% H_2_O), open circle |
| x30001015 | 13 | 2.144E–5 (CO) | N_2_/H_2_O | 0.0056 | 1.05 | 882–1199 | Fig. 6 bottom, set 3 (9% H_2_O), open circle |
| **Alzueta et al. (2001)** [[84](#_ENREF_84)] | | | | | | | |
| x30001016 | 10 | 9.406E–6 (CO) | N_2_/H_2_O | 0.0005 | 1.04 | 893–1201 | Fig. 4 top, open circle, set 1a |
| x30001017 | 14 | 7.454E–6 (CO) | N_2_/H_2_O | 0.0006 | 1.04 | 891–1220 | Fig. 4 bottom, open circle, set 2a |
| x30001018 | 11 | 1.291E–4 (CO) | N_2_/H_2_O | 3.03 | 1.04 | 875–1381 | Fig. 5 top, open circle, set 3a |
| x30001019 | 15 | 7.816E–5 (CO) | N_2_/H_2_O | 1.78 | 1.04 | 891–1295 | Fig. 5 middle, open circle, set 4a |
| x30001020 | 15 | 2.723E–4 (CO) | N_2_/H_2_O | 1.25 | 1.04 | 785–1449 | Fig. 5 bottom, open circle, set 5a |
| x30001022 | 19 | 1.070E–6 (CO) | N_2_/H_2_O | 0.48 | 1.04 | 900–1430 | Fig. 3 bottom, open circle, set 7a |

**Table S12** Shock tube outlet concentration measurements of syngas−oxygen mixtures

| **ID [.xml]** | ***N*_i_** | ***σ*** | **Diluents** | ***φ*** | ***p* / atm** | ***T* / K** | **Comment** |
| --- | --- | --- | --- | --- | --- | --- | --- |
| **Sivaramakrishnan et al. (2007)** [[85](#_ENREF_85)] | | | | | | | |
| x50001001 | 32  32  17 | 8.250E–6 (CO)  1.215E–5 (CO_2_)  1.138E–5 (O_2_) | Ar | 0.95 | 21.0–29.7 | 995–1483 | Supp. Mat. Table S1 |
| x50001003 | 24  24  24 | 6.082E–6 (CO)  5.308E–6 (CO_2_)  7.096E–6 (O_2_) | Ar | 0.95 | 224.6–296.0 | 1109–1448 | Supp. Mat. Table S3 |
| x50001004 | 16  16  16 | 5.943E–6 (CO)  8.152E–6 (CO_2_)  9.742E–6 (O_2_) | Ar | 1.00 | 400.8–499.5 | 1128–1414 | Supp. Mat. Table S4 |
| x50001005 | 23  23  21 | 8.488E–6 (CO)  1.215E–5 (CO_2_)  1.285E–5 (O_2_) | Ar | 0.50 | 20.9–27.2 | 1044–1456 | Supp. Mat. Table S5 |
| x50001006 | 21  21  19 | 9.629E–6 (CO)  1.096E–5 (CO_2_)  1.086E–5 (O_2_) | Ar | 0.50 | 39.5–49.0 | 1069–1495 | Supp. Mat. Table S6 |
| x50001007 | 18  18  18 | 9.394E–6 (CO)  1.461E–5 (CO_2_)  1.040E–5 (O_2_) | Ar | 0.47 | 224.6–285.8 | 1157–1429 | Supp. Mat. Table S7 |

**Table S13** Jet-stirred reactor outlet concentration measurements of syngas−oxygen mixtures

| **ID [.xml]** | ***N*_i_** | ***σ*** | **Diluents** | ***φ*** | ***p* / atm** | ***T* / K** | **Comment** |
| --- | --- | --- | --- | --- | --- | --- | --- |
| **Dagaut et al. (2003)** [[86](#_ENREF_86)] | | | | | | | |
| x00001001 | 8  8  8 | 6.761E–7 (H_2_)  1.262E–5 (CO)  4.345E–5 (CO_2_) | N_2_ | 0.1 | 1 | 850–1200 | Fig. 1a, CO_2_, CO and H_2_ profiles measured |
| x00001002 | 9  9  9 | 1.002E–4 (H_2_)  1.074E–4 (CO)  6.300E–5 (CO_2_) | N_2_ | 1.0 | 1 | 850–1400 | Fig. 2a, CO_2_, CO and H_2_ profiles measured |

# Utilized direct rate coefficient measurements

**Table S14**

Direct measurements considered in the optimization

| Authors | ID | Reference | Bath gas | Number of  data points | *σ*(ln*k*) |
| --- | --- | --- | --- | --- | --- |
| **R1 Ḣ+O_2_ =Ö + ȮH** | | | | | |
| Masten *et al.* (1990) | k00000001 | [[87](#_ENREF_87)] | Ar | 30 | 0.10 |
| Masten *et al.* (1990) | k00000002 | [[87](#_ENREF_87)] | Ar | 14 | 0.24 |
| Du and Hessler (1992) | k00000003 | [[88](#_ENREF_88)] | Ar | 11 | 0.10 |
| Yang *et al.* (1994) | k00000004 | [[89](#_ENREF_89)] | Ar | 20 | 0.10 |
| Ryu *et al.* (1995) | k00000005 | [[90](#_ENREF_90)] | Ar | 178 | 0.10 |
| Pirraglia *et al.* (1989) | k00000008 | [[91](#_ENREF_91)] | Ar | 159 | 0.19 |
| Shin and Michael (1991) | k00000009 | [[92](#_ENREF_92)] | Ar | 124 | 0.27 |
| Hwang *et al.*(2005) | k00000012 | [[93](#_ENREF_93)] | Ar | 189 | 0.10 |
| Hong *et al.* (2011) | k00000050 | [[94](#_ENREF_94)] | Ar | 20 | 0.07 |
| **R2 Ö + H_2_ = Ḣ + ȮH** | | | | | |
| Ryu *et al.* (1995) | k00000019 | [[95](#_ENREF_95)] | Ar | 50 | 0.10 |
| Davidson and Hanson (1990) | k00000020 | [[96](#_ENREF_96)] | Ar | 13 | 0.10 |
| Presser and Gordon (1985) | k00000021 | [[97](#_ENREF_97)] | Ar | 9 | 0.10 |
| Light and Matsumoto (1980) | k00000022 | [[98](#_ENREF_98)] | Ar | 22 | 0.17 |
| Javoy *et al.* (2000) | k00000035 | [[99](#_ENREF_99)] | Ar | 29 | 0.10 |
| Natarajan *et al.* (1987) | k00000036 | [[100](#_ENREF_100)] | Ar | 37 | 0.10 |
| Natarajan *et al.* (1987) | k00000037 | [[100](#_ENREF_100)] | Ar | 11 | 0.10 |
| Sutherland *et al.* (1986) | k00000038 | [[101](#_ENREF_101)] | Ar | 112 | 0.13 |
| Sutherland *et al.* (1986) | k00000039 | [[101](#_ENREF_101)] | Ar | 46 | 0.10 |
| Yang *et al.* (1993) | k00000057 | [[102](#_ENREF_102)] | Ar | 9 | 0.15 |
| **R3 ȮH + H_2_ = Ḣ + H_2_O** | | | | | |
| Michael and Sutherland (1988) | k00000023 | [[103](#_ENREF_103)] | Ar | 105 | 0.21 |
| Oldenborg *et al.* (1992) | k00000024 | [[104](#_ENREF_104)] | Ar | 20 | 0.10 |
| Davidson (1988) | k00000025 | [[105](#_ENREF_105)] | Ar | 19 | 0.20 |
| Frank and Just (1985) | k00000026 | [[106](#_ENREF_106)] | Ar | 19 | 0.12 |
| Ravishankara *et al.* (1981) | k00000027 | [[107](#_ENREF_107)] | Ar | 10 | 0.10 |
| Tully and Ravishankara (1980) | k00000028 | [[108](#_ENREF_108)] | Ar | 8 | 0.10 |
| Lam *et al.* (2013) | k00000060 | [[109](#_ENREF_109)] | Ar | 21 | 0.10 |
| **R4 ȮH + ȮH = Ö + H_2_O** | | | | | |
| Sutherland *et al.* (1990) | k00000029 | [[110](#_ENREF_110)] | Ar | 104 | 0.20 |
| Lifshitz *et al.* (1990) | k00000030 | [[111](#_ENREF_111)] | He | 64 | 0.17 |
| Wagner (1981) | k00000031 | [[112](#_ENREF_112)] | Ar | 3 | 0.30 |
| Gardiner *et al.* (1972) | k00000065 | [[113](#_ENREF_113)] | Ar | 2 | 0.30 |
| **R5 Ḣ + Ḣ + M = H_2_ + M** | | | | | |
| Gay *et al.* (1971) | k00000078 | [[114](#_ENREF_114)] | Ar | 2 | 0.20 |
| **R8 Ḣ + ȮH + M = H_2_O + M** | | | | | |
| Halstead and Jenkins (1969) | k00000070 | [[115](#_ENREF_115)] | N_2_ | 2 | 0.10 |

| **R9 Ḣ + O_2_ + M = HȮ_2_ +M** | | | | | |
| --- | --- | --- | --- | --- | --- |
| Mueller *et al.* (1998) | k00000006 | [[116](#_ENREF_116)] | N_2_ | 6 | 0.10 |
| Mueller *et al.* (1998) | k00000007 | [[116](#_ENREF_116)] | Ar | 4 | 0.10 |
| Ashman and Haynes (1998) | k00000010 | [[117](#_ENREF_117)] | Ar | 7 | 0.10 |
| Ashman and Haynes (1998) | k00000011 | [[117](#_ENREF_117)] | N_2_ | 10 | 0.10 |
| Getzinger and Schott (1965) | k00000013 | [[118](#_ENREF_118)] | Ar | 96 | 0.15 |
| Getzinger and Blair (1969) | k00000014 | [[119](#_ENREF_119)] | N_2_ | 10 | 0.12 |
| Blair and Getzinger (1970) | k00000015 | [[120](#_ENREF_120)] | Ar | 26 | 0.42 |
| Michael *et al.* (2002) | k00000016 | [[121](#_ENREF_121)] | N_2_ | 14 | 0.10 |
| Michael *et al.* (2002) | k00000017 | [[121](#_ENREF_121)] | Ar | 19 | 0.10 |
| Gay and Pratt (1971) | k00000081 | [[114](#_ENREF_114)] | Ar | 2 | 0.20 |
| **R10 HȮ_2_+ Ḣ= H_2_ + O_2_** | | | | | |
| Michael *et al.* (2000) | k00000034 | [[122](#_ENREF_122)] | Ar | 28 | 0.44 |
| **R13 HȮ_2_ + ȮH = H_2_O + O_2_** | | | | | |
| Hong *et al.* (2010) | k00000042 | [[123](#_ENREF_123)] | Ar | 11 | 0.10 |
| Hong *et al.* (2013) | k00000052 | [[124](#_ENREF_124)] | Ar | 15 | 0.12 |
| Srinivasan *et al.* (2006) | k00000083 | [[125](#_ENREF_125)] | Ar | 24 | 0.42 |
| **R15 HȮ_2_ + HȮ_2_ = H_2_O_2_ + O_2_** | | | | | |
| Hong *et al.* (2013) | k00000053 | [[124](#_ENREF_124)] | Ar | 16 | 0.19 |
| Hippler *et al.* (1990) | k00000061 | [[126](#_ENREF_126)] | Ar | 16 | 0.22 |
| Hippler *et al.* (1990) | k00000062 | [[126](#_ENREF_126)] | Ar | 27 | 0.23 |
| Kappel *et al.* (2002) | k00000063 | [[127](#_ENREF_127)] | Ar | 13 | 0.10 |
| **R16 ȮH + ȮH + M = H_2_O_2_ + M** | | | | | |
| Hong *et al.* (2009) | k00000032 | [[128](#_ENREF_128)] | Ar | 40 | 0.10 |
| Hong *et al.* (2010) | k00000033 | [[129](#_ENREF_129)] | Ar | 28 | 0.10 |
| Kappel *et al.* (2002) | k00000045 | [[127](#_ENREF_127)] | Ar | 13 | 0.21 |
| Kappel *et al.* (2002) | k00000046 | [[127](#_ENREF_127)] | Ar | 7 | 0.23 |
| Kappel *et al.* (2002) | k00000047 | [[127](#_ENREF_127)] | Ar | 7 | 0.31 |
| Hong *et al.* (2012) | k00000075 | [[130](#_ENREF_130)] | Ar | 18 | 0.10 |
| **R23 CO + O_2_ = CO_2_ + Ö** | | | | | |
| Thielen *et al.* (1983) | k50001001 | [[131](#_ENREF_131)] | Ar | 39 | 0.19 |
| **R24 CO + ȮH = CO_2_ + Ḣ** | | | | | |
| Wooldridge *et al.* (1994) | k10001001 | [[132](#_ENREF_132)] | Ar | 44 | 0.10 |
| Westenberg *et al.* (1973) | k10001002 | [[133](#_ENREF_133)] | He | 5 | 0.10 |
| Ravishankara *et al.* (1983) | k10001003 | [[134](#_ENREF_134)] | Ar | 21 | 0.10 |
| Brabbs *et al.* (1970) | k10001004 | [[135](#_ENREF_135)] | Ar | 15 | 0.29 |
| Golden *et al.* (1998) | k10001005 | [[136](#_ENREF_136)] | He | 4 | 0.10 |
| Golden *et al.* (1998) | k10001006 | [[136](#_ENREF_136)] | He | 5 | 0.10 |
| Golden *et al.* (1998) | k10001007 | [[136](#_ENREF_136)] | He | 3 | 0.10 |
| Golden *et al.* (1998) | k10001008 | [[136](#_ENREF_136)] | He | 2 | 0.10 |
| Golden *et al.* (1998) | k10001009 | [[136](#_ENREF_136)] | He | 5 | 0.10 |
| Gardiner *et al.* (1973) | k10001013 | [[113](#_ENREF_113)] | Ar | 2 | 0.20 |
| Wooldridge *et al.* (1996) | k10001014 | [[137](#_ENREF_137)] | Ar | 48 | 0.10 |
| Vandooren *et al.* (1975) | k10001015 | [[138](#_ENREF_138)] | O_2_ | 27 | 0.10 |
| Peeters *et al.* (1972) | k10001016 | [[139](#_ENREF_139)] | Ar | 10 | 0.10 |
| Eberius *et al.* (1973) | k10001017 | [[140](#_ENREF_140)] | O_2_ | 9 | 0.17 |
| Eberius *et al.* (1973) | k10001018 | [[140](#_ENREF_140)] | O_2_ | 8 | 0.10 |

| **R26 HĊO + M = Ḣ + CO + M** | | | | | |
| --- | --- | --- | --- | --- | --- |
| Hippler *et al.* (2004) | k20001008 | [[141](#_ENREF_141)] | He | 23 | 0.10 |
| Hippler *et al.* (2004) | k20001009 | [[141](#_ENREF_141)] | He | 29 | 0.11 |
| Hippler *et al.* (2004) | k20001010 | [[141](#_ENREF_141)] | He | 31 | 0.14 |
| Hippler *et al.* (2004) | k20001011 | [[141](#_ENREF_141)] | He | 25 | 0.16 |
| Hippler *et al.* (2004) | k20001012 | [[141](#_ENREF_141)] | He | 35 | 0.14 |
| Hippler *et al.* (2004) | k20001013 | [[141](#_ENREF_141)] | N_2_ | 8 | 0.17 |
| Krasnoperov *et al.* (2004) | k20001014 | [[142](#_ENREF_142)] | He | 11 | 0.33 |
| Krasnoperov *et al.* (2004) | k20001015 | [[142](#_ENREF_142)] | He | 8 | 0.10 |

# Arrhenius plots of the optimized rate coefficients

The Arrhenius plots show the optimized rate coefficients and their posterior uncertainties with red solid and dashed lines, respectively. The mean values and uncertainty limits of Nagy *et al.* are given with blue solid and dashed lines, respectively. Additional results from experimental and theoretical studies, and recommended values from review articles are also plotted.


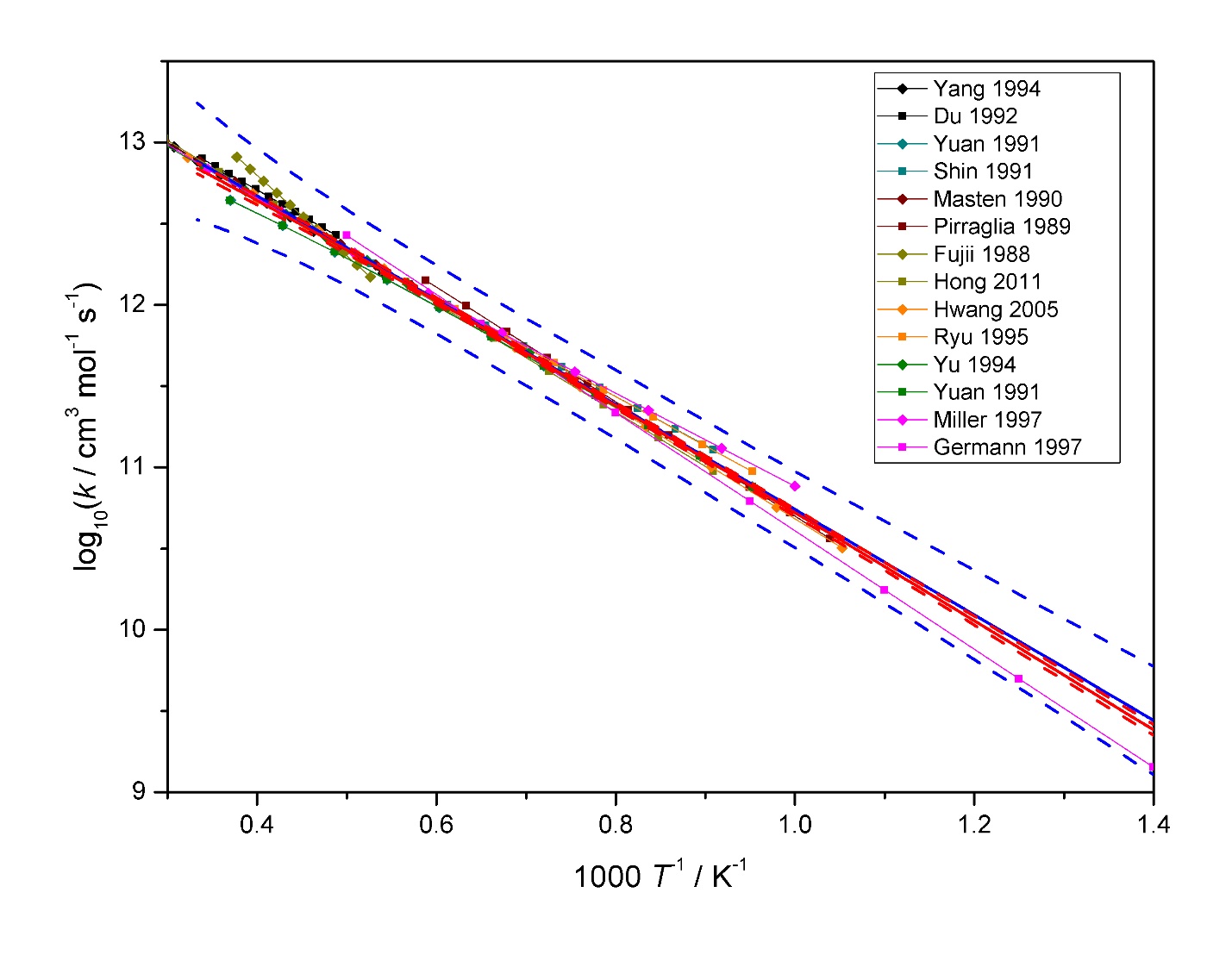


**Figure S1** Arrhenius plot of optimized rate coefficient of reaction Ḣ + O_2_ = Ö + ȮH (R1). Additional results from [[87-89](#_ENREF_87), [91-95](#_ENREF_91), [143-147](#_ENREF_143)] are also plotted.


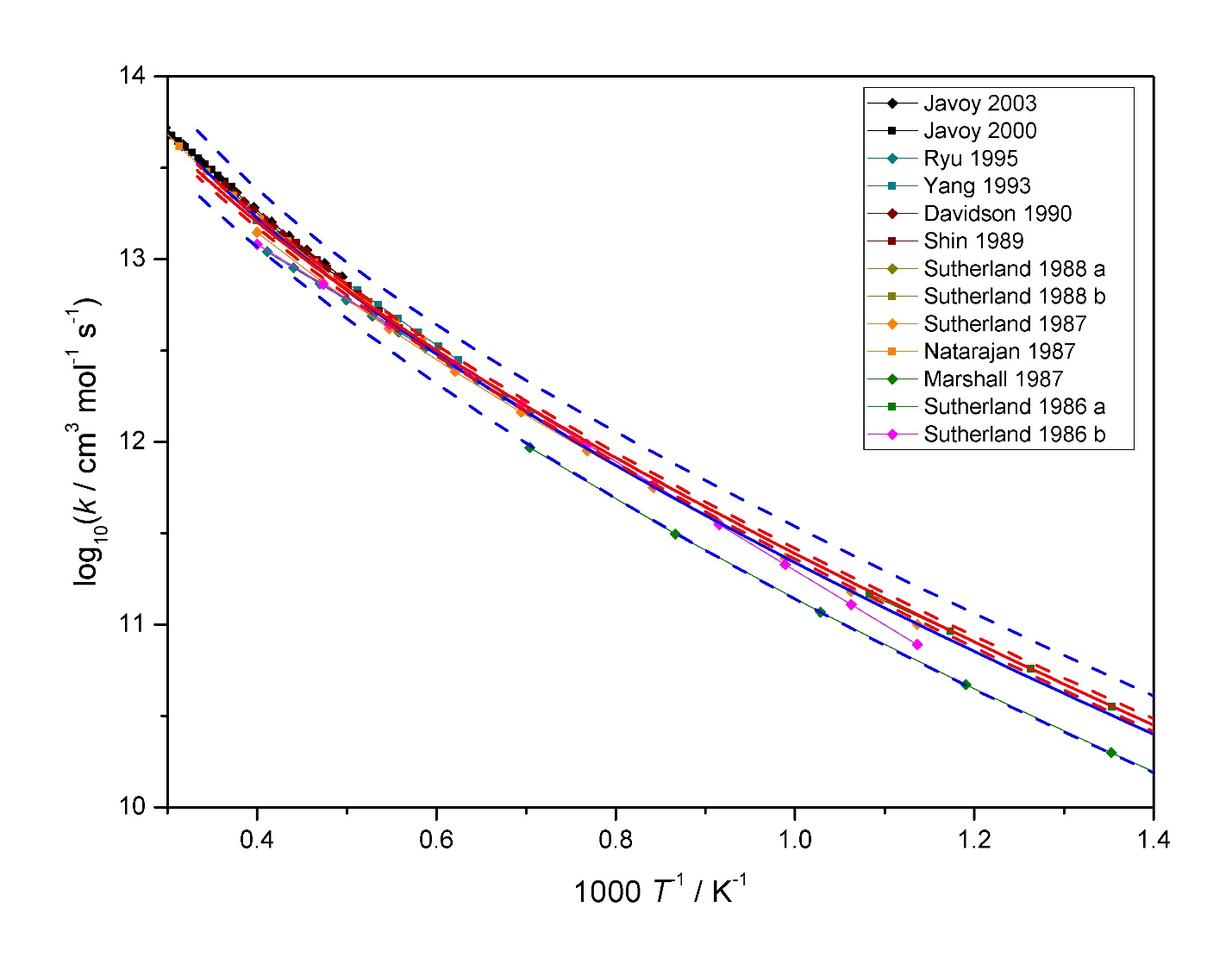


**Figure S2** Arrhenius plot of optimized rate coefficient of reaction Ö + H_2_ = Ḣ + ȮH (R2). Additional results from [[95](#_ENREF_95), [96](#_ENREF_96), [99-102](#_ENREF_99), [148-151](#_ENREF_148)] are also plotted.


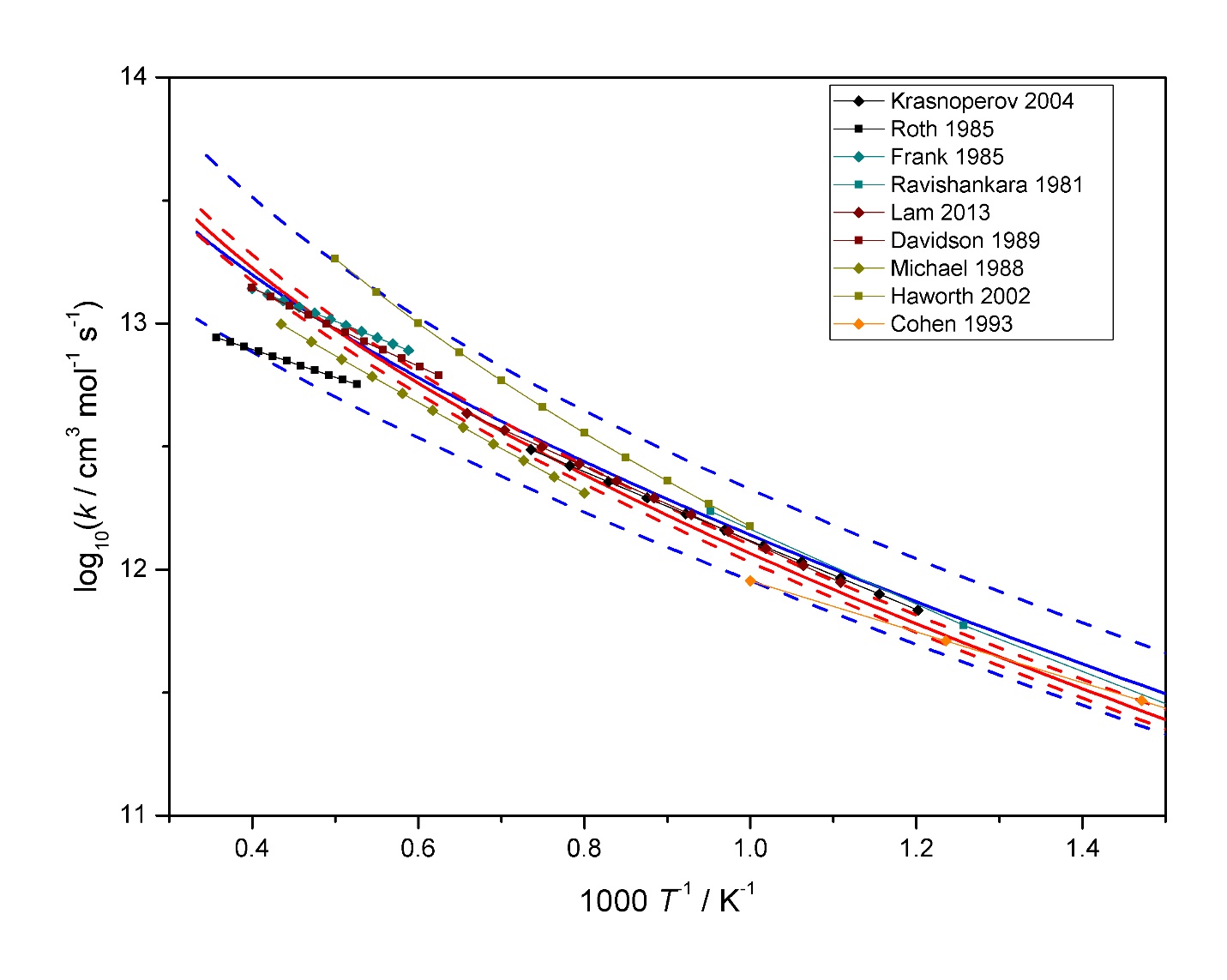


**Figure S3** Arrhenius plot of optimized rate coefficient of reaction ȮH + H_2_ = Ḣ + H_2_O (R3). Additional results from [[103](#_ENREF_103), [105-107](#_ENREF_105), [109](#_ENREF_109), [142](#_ENREF_142), [152-154](#_ENREF_152)] are also plotted.


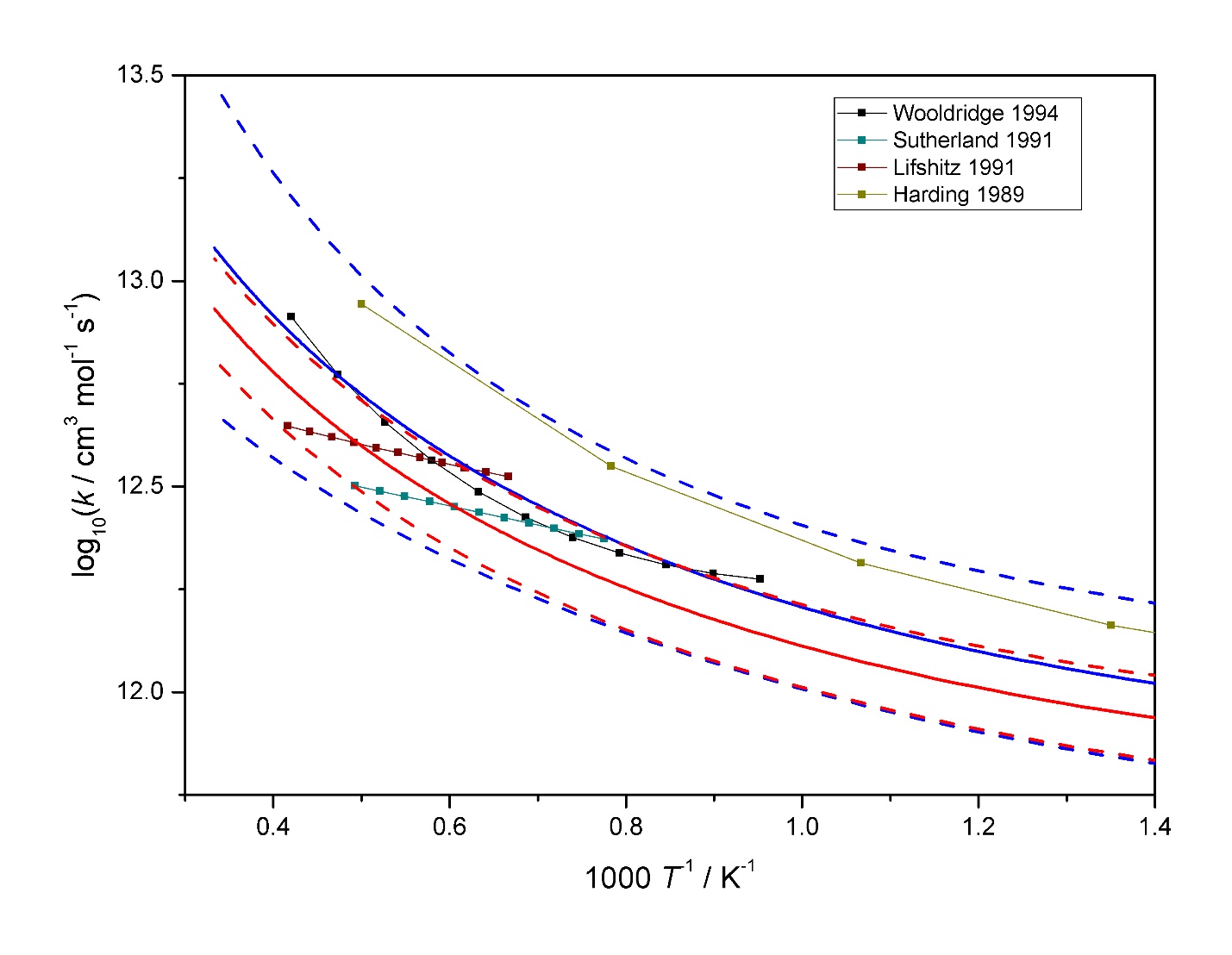


**Figure S4** Arrhenius plot of optimized rate coefficient of reaction ȮH + ȮH = Ö + H_2_O (R4). Additional results from [[111](#_ENREF_111), [155-157](#_ENREF_155)] are also plotted.


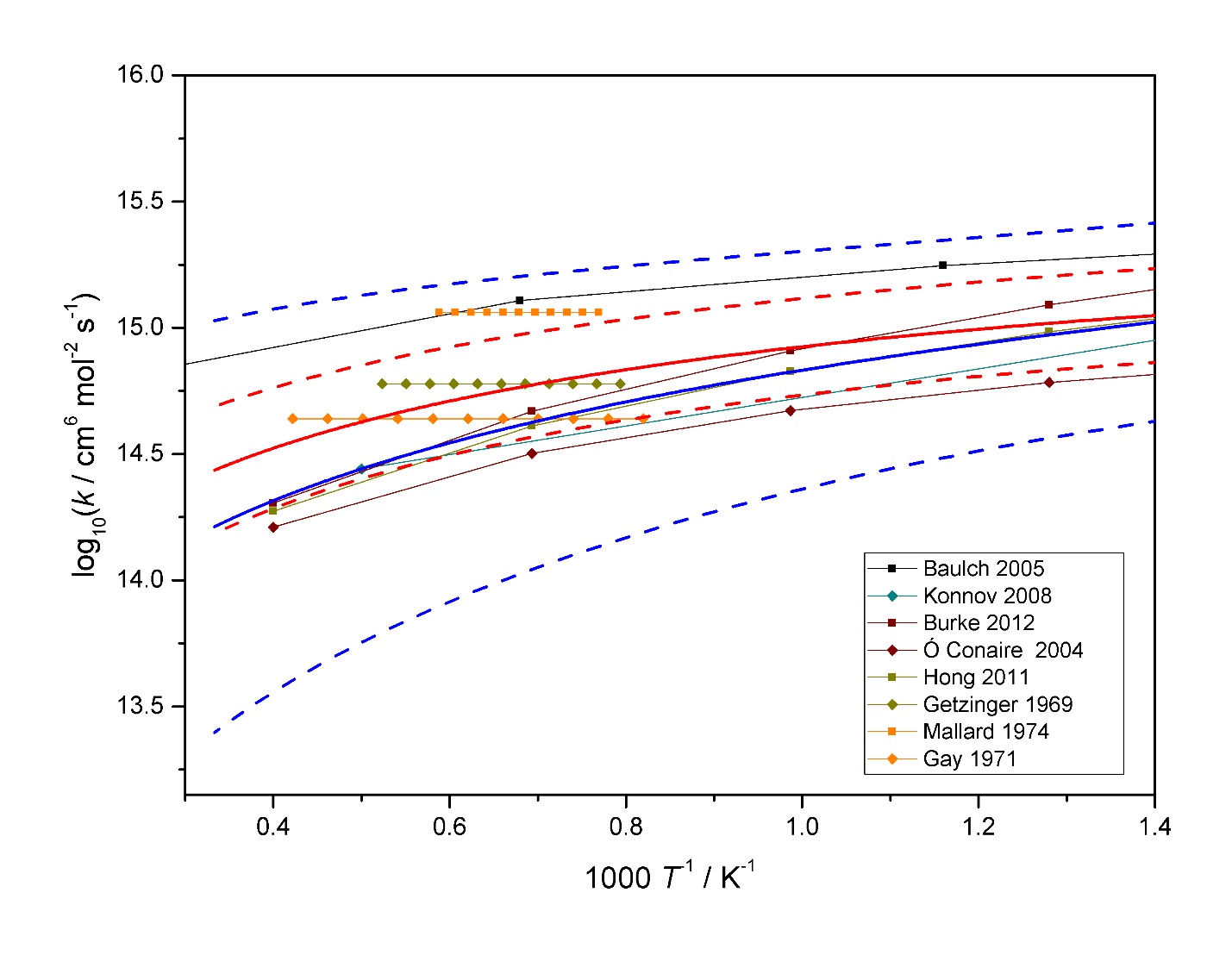


**Figure S5** Arrhenius plot of optimized low-pressure limiting rate coefficient of reaction Ḣ + Ḣ + M = H_2_ + M (R5). Additional results from [[114](#_ENREF_114), [119](#_ENREF_119), [158-163](#_ENREF_158)] are also plotted.


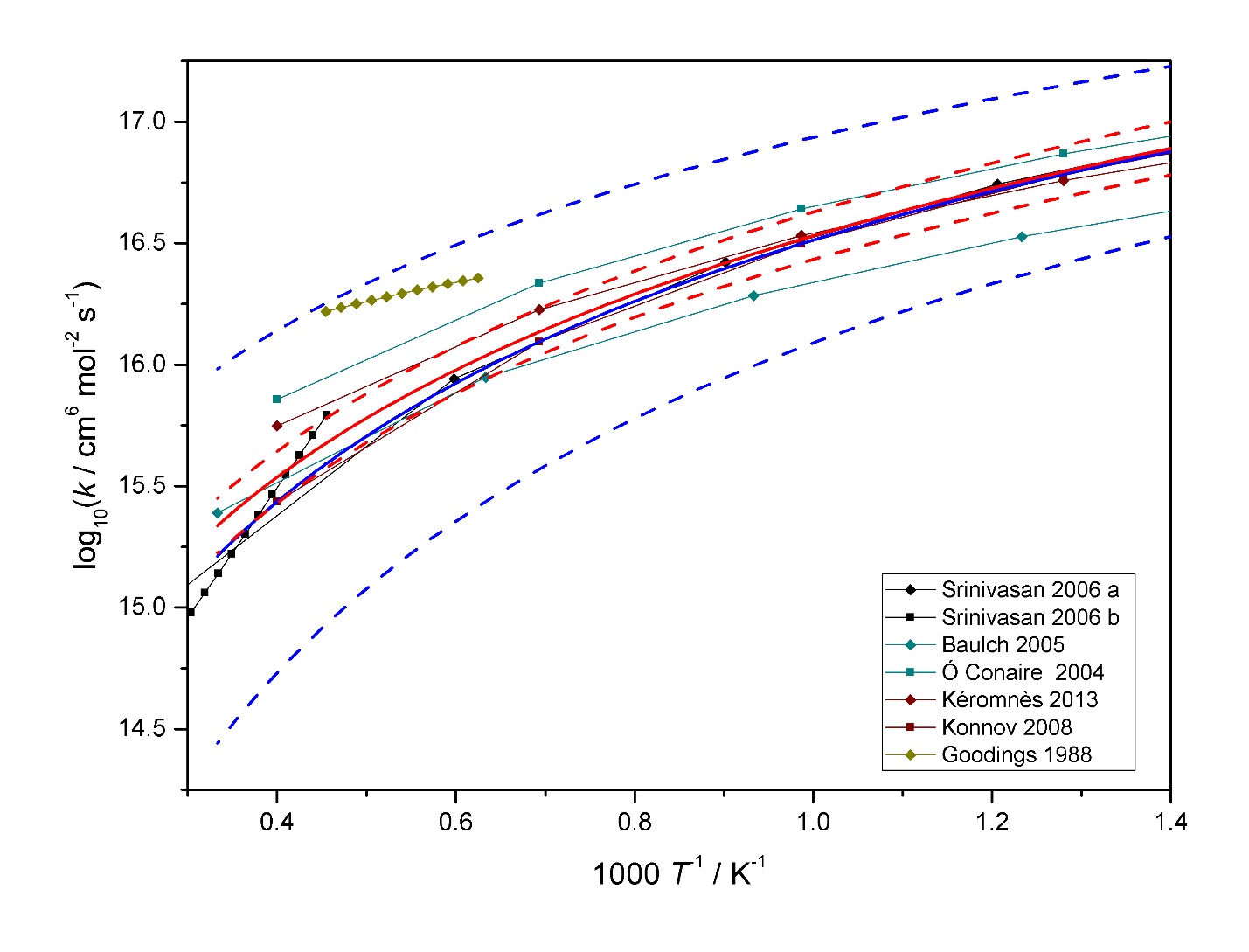


**Figure S6** Arrhenius plot of optimized low-pressure limiting rate coefficient of reaction Ḣ + ȮH + M = H_2_O + M (R8). Additional results from [[17](#_ENREF_17), [125](#_ENREF_125), [158](#_ENREF_158), [159](#_ENREF_159), [161](#_ENREF_161), [164](#_ENREF_164)] are also plotted.


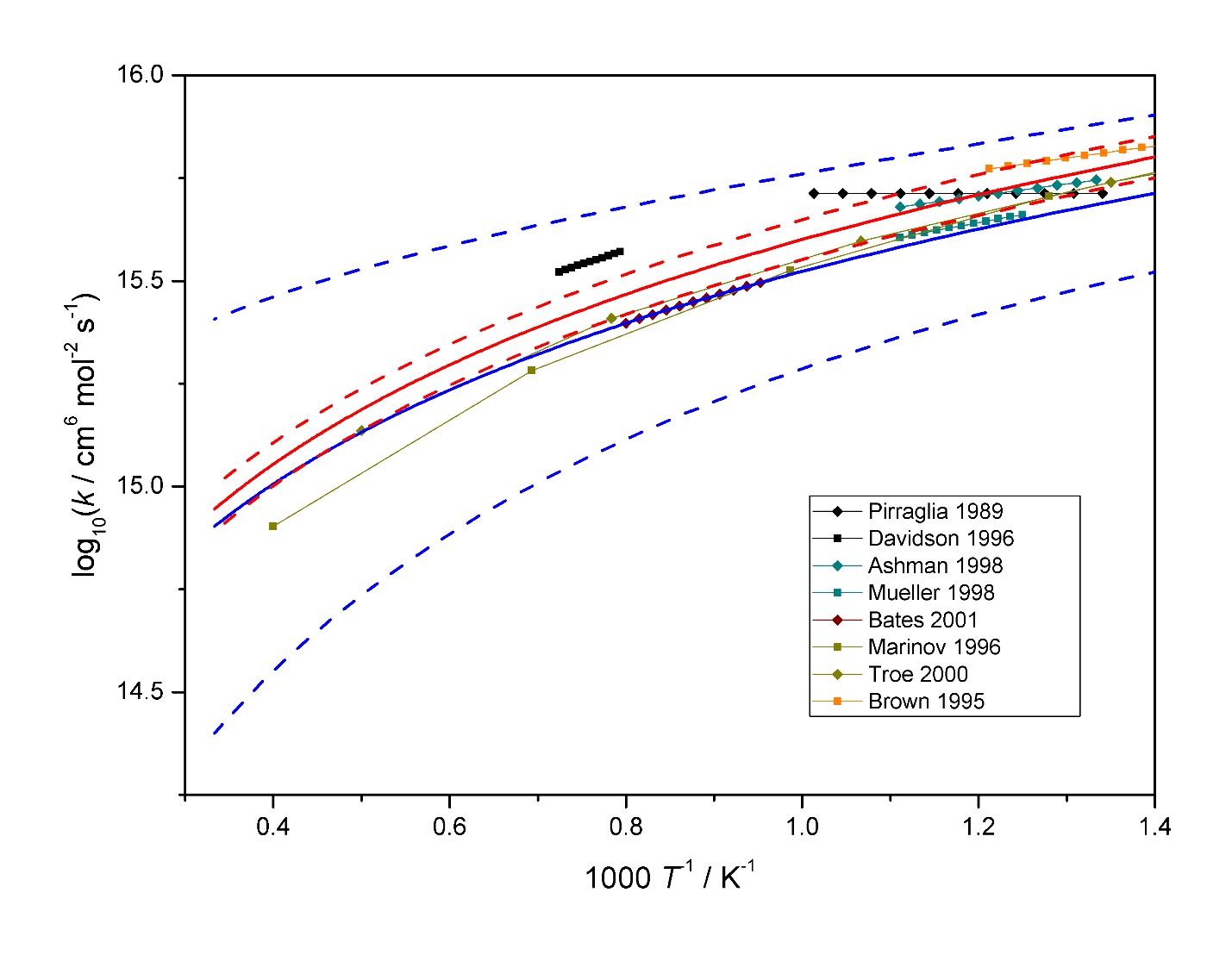


**Figure S7** Arrhenius plot of optimized low-pressure limiting rate coefficient of reaction Ḣ + O_2_ + M = HȮ_2_ + M (R9). Additional results from [[91](#_ENREF_91), [116](#_ENREF_116), [165-170](#_ENREF_165)] are also plotted.


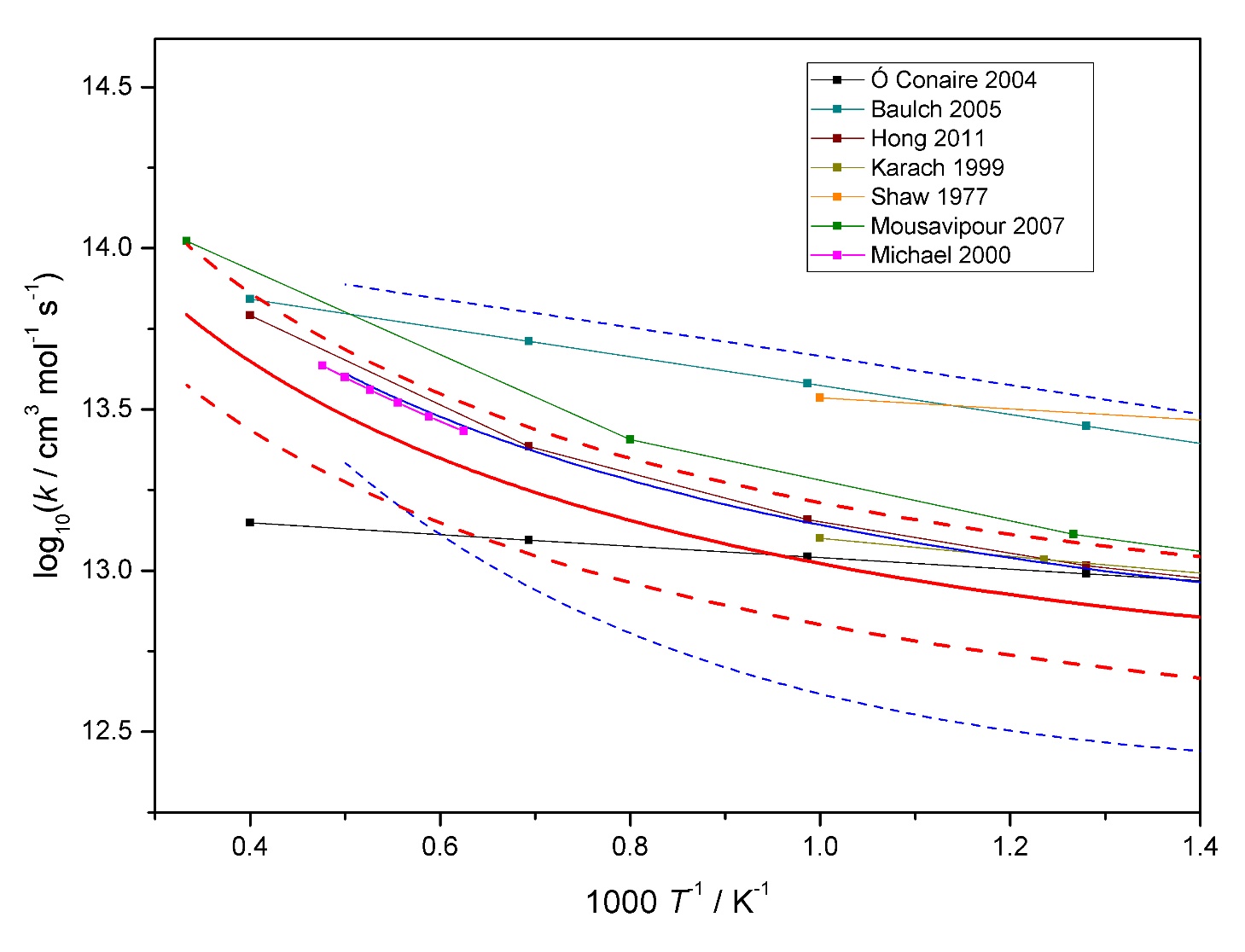


**Figure S8** Arrhenius plot of optimized rate coefficient of reaction Ḣ + HȮ_2_ = H_2_ + O_2_ (R10). Additional results from [[122](#_ENREF_122), [158](#_ENREF_158), [161](#_ENREF_161), [162](#_ENREF_162), [171-173](#_ENREF_171)] are also plotted.


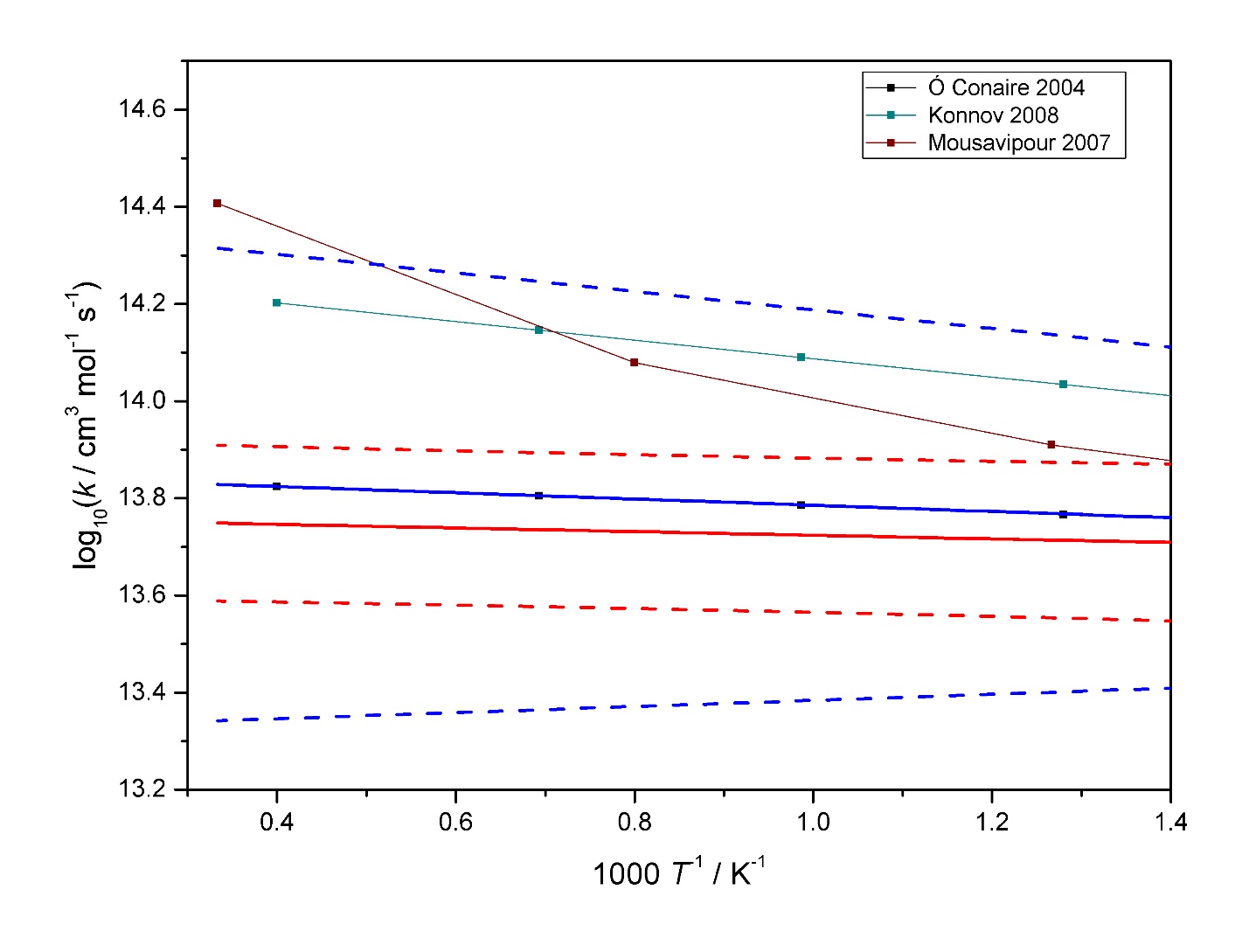


**Figure S9** Arrhenius plot of optimized rate coefficient of reaction HȮ_2_ + Ḣ = ȮH + ȮH (R11). Additional results from [[159](#_ENREF_159), [161](#_ENREF_161), [173](#_ENREF_173)] are also plotted.


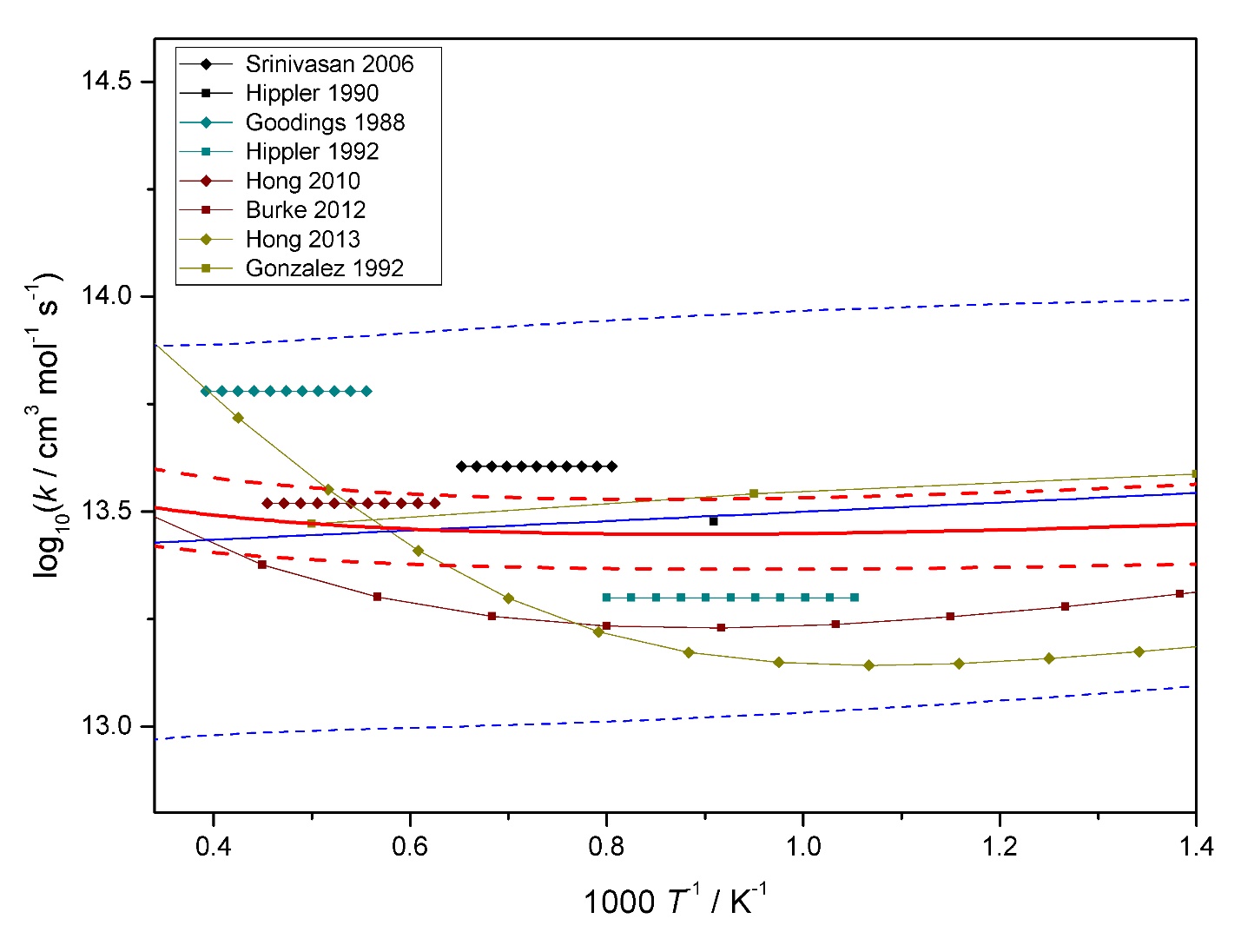


**Figure S10** Arrhenius plot of optimized rate coefficient of reaction HȮ_2_ + ȮH = H_2_O + O_2_ (R13). Additional results from [[123-126](#_ENREF_123), [160](#_ENREF_160), [164](#_ENREF_164), [174](#_ENREF_174), [175](#_ENREF_175)] are also plotted.


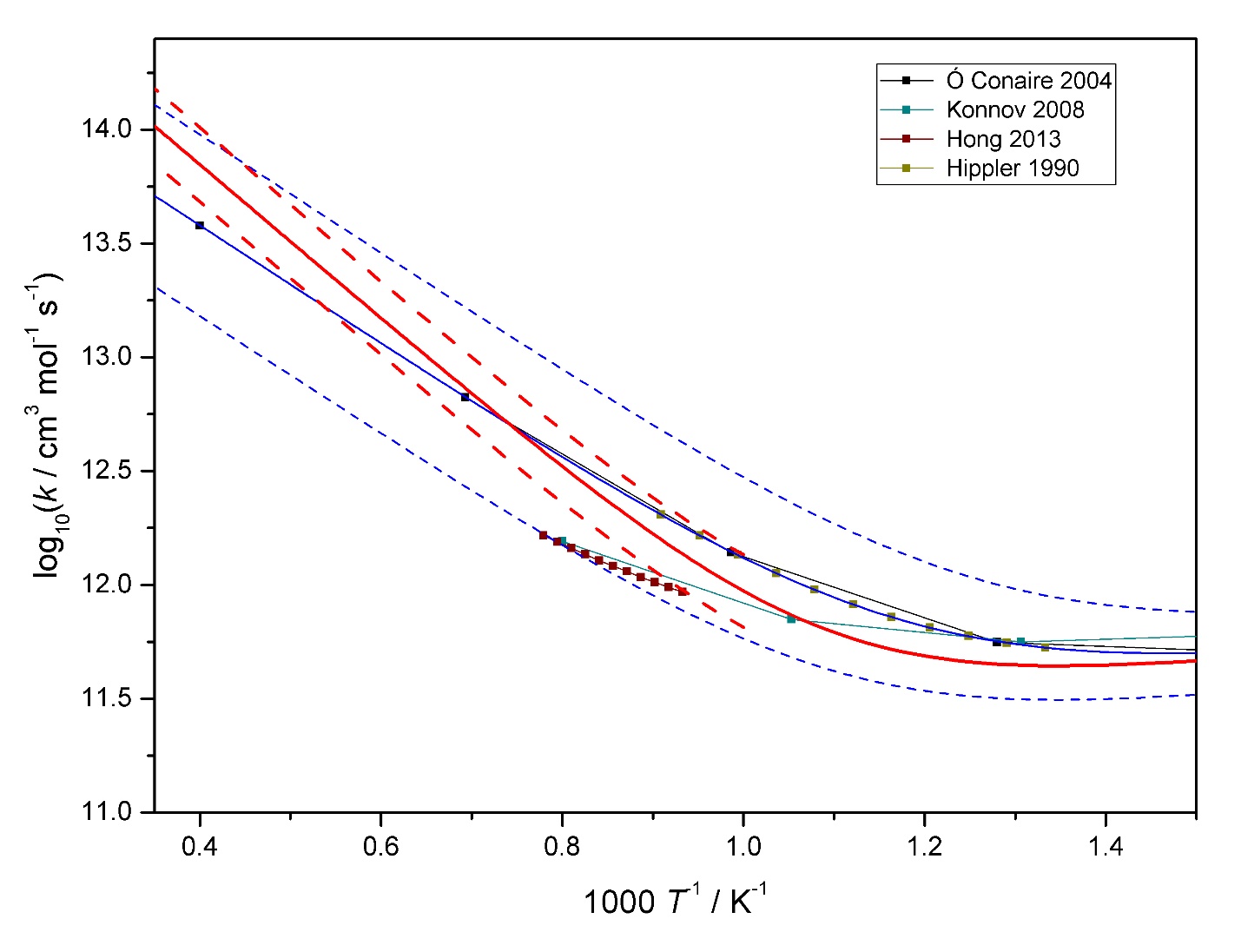


**Figure S11** Arrhenius plot of optimized rate coefficient of reaction HȮ_2_ + HȮ_2_ = H_2_O_2_ + O_2_ (R15). Additional results from [[124](#_ENREF_124), [126](#_ENREF_126), [159](#_ENREF_159), [161](#_ENREF_161)] are also plotted.


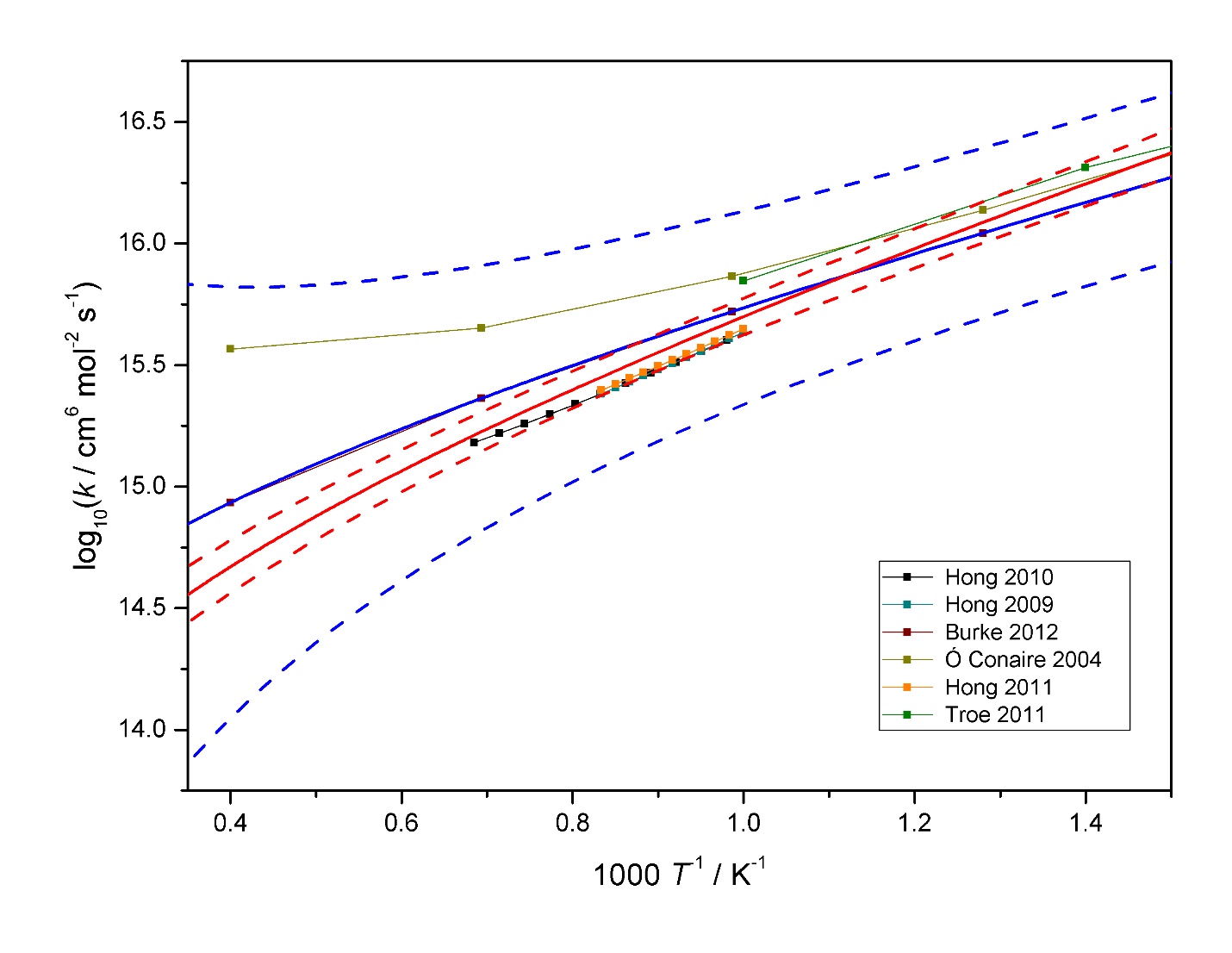


**Figure S12** Arrhenius plot of optimized low-pressure limiting rate coefficient of reaction ȮH + ȮH + M = H_2_O_2_ + M (R16). Additional results from [[128](#_ENREF_128), [129](#_ENREF_129), [160-162](#_ENREF_160), [169](#_ENREF_169)] are also plotted.


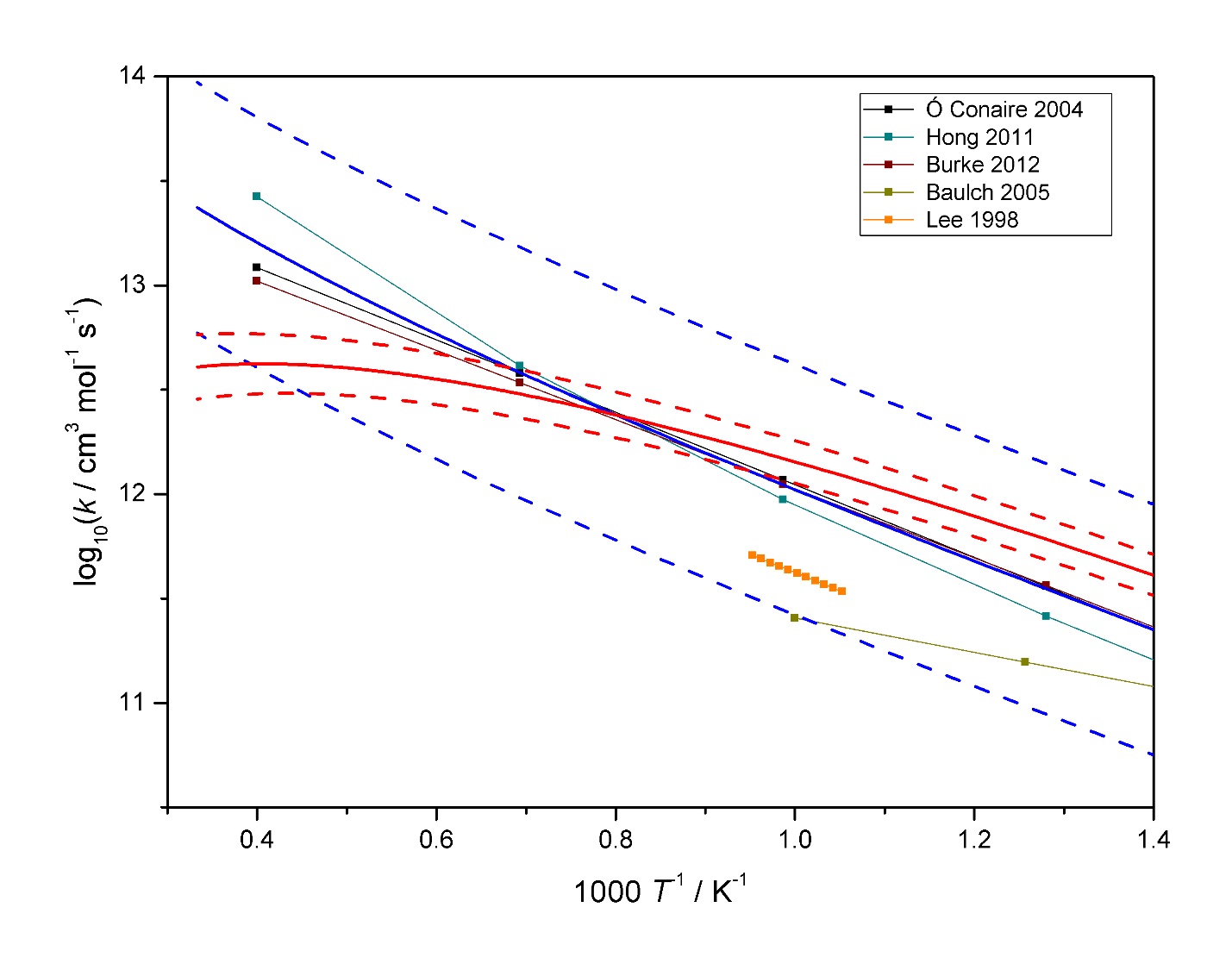


**Figure S13** Arrhenius plot of optimized rate coefficient of reaction H_2_O_2_ + Ḣ = H_2_ + HȮ_2_ (R18). Additional results from [[158](#_ENREF_158), [160-162](#_ENREF_160), [176](#_ENREF_176)] are also plotted.


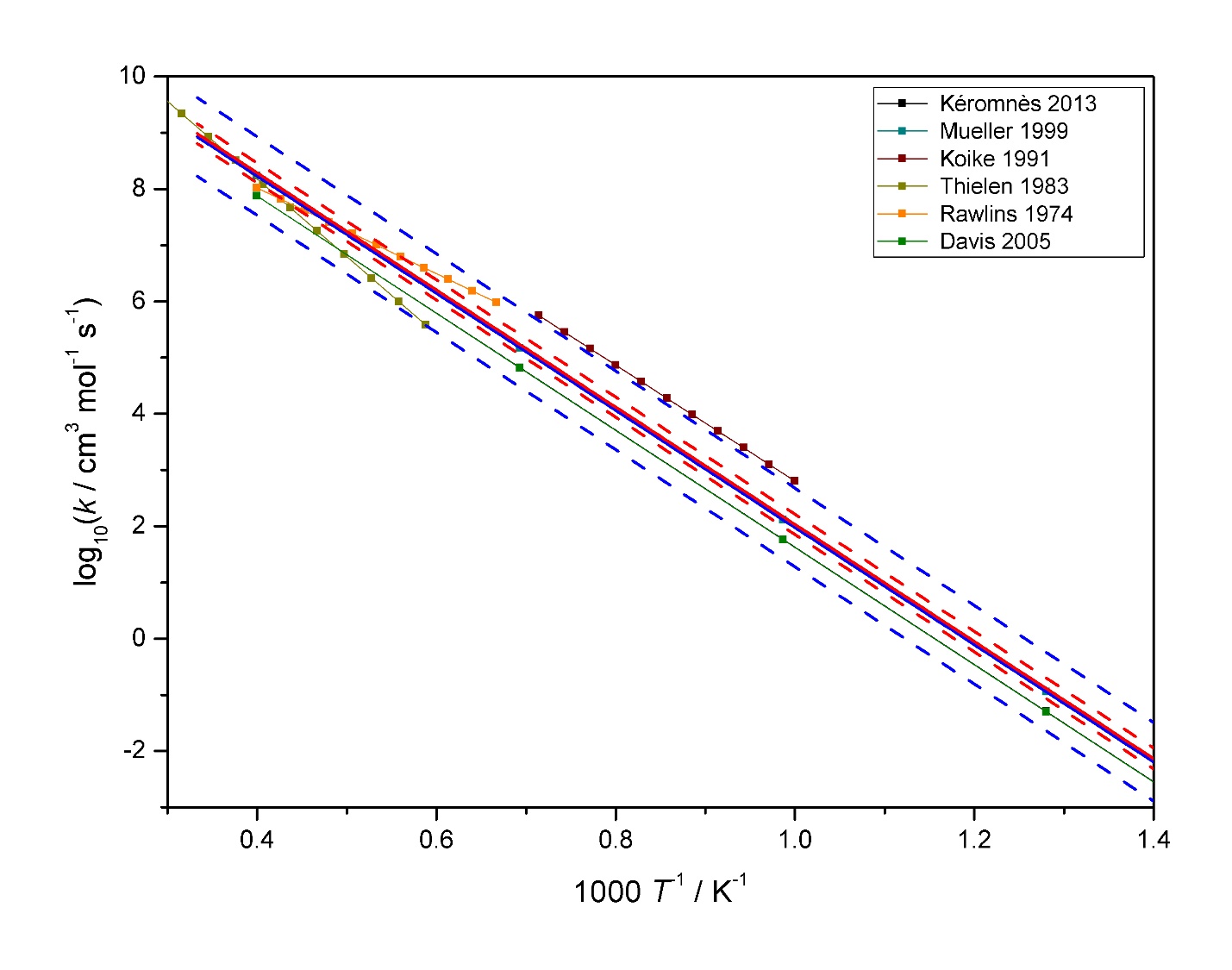


**Figure S14** Arrhenius plot of optimized rate coefficient of reaction CO + O_2_ = CO_2_ + Ö (R23). Additional results from [[17](#_ENREF_17), [20](#_ENREF_20), [131](#_ENREF_131), [177-179](#_ENREF_177)] are also plotted.


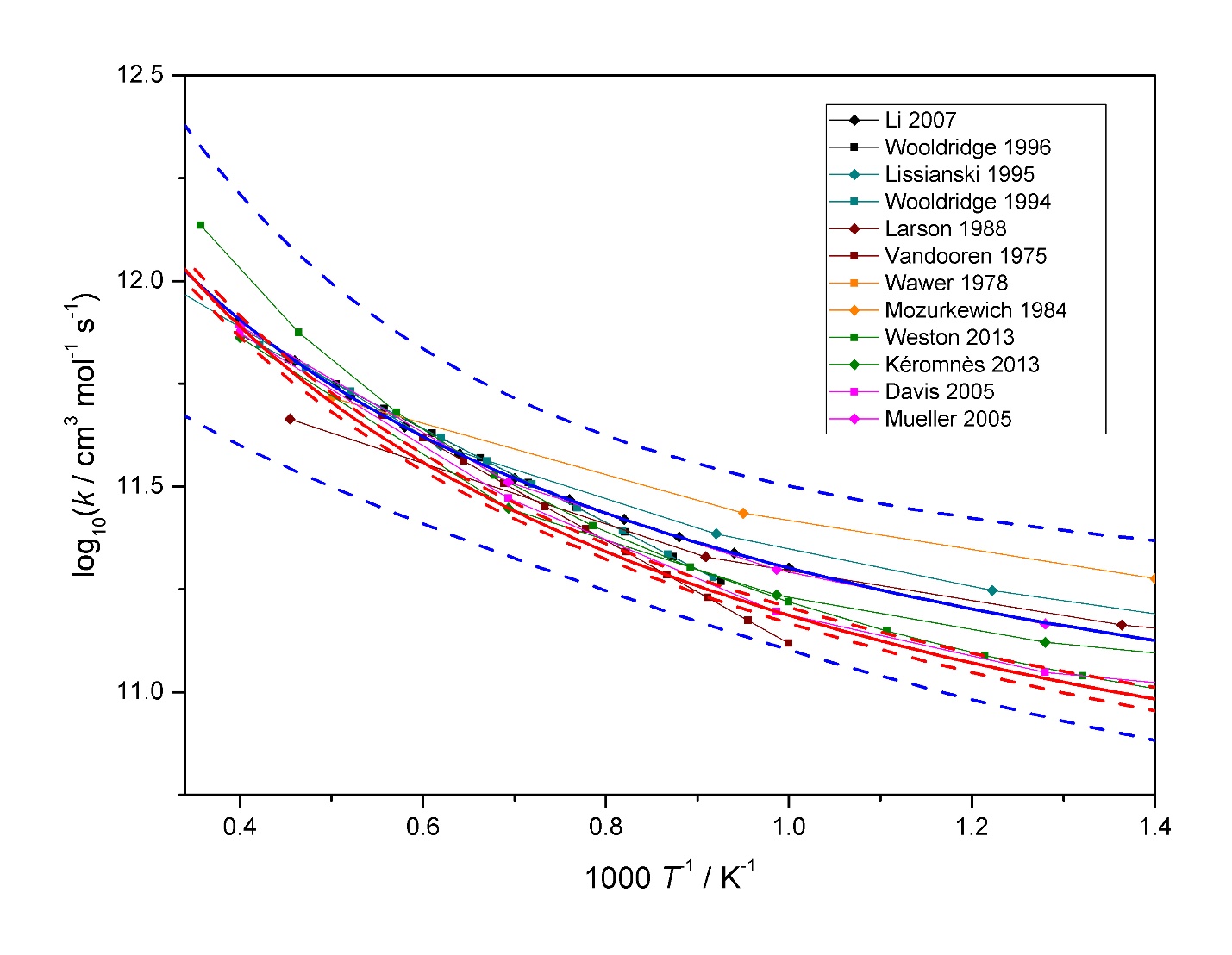


**Figure S15** Arrhenius plot of optimized rate coefficient of reaction CO + ȮH = CO_2_ + Ḣ (R24). Additional results from [[17](#_ENREF_17), [80](#_ENREF_80), [82](#_ENREF_82), [132](#_ENREF_132), [137](#_ENREF_137), [138](#_ENREF_138), [179-184](#_ENREF_179)] are also plotted.


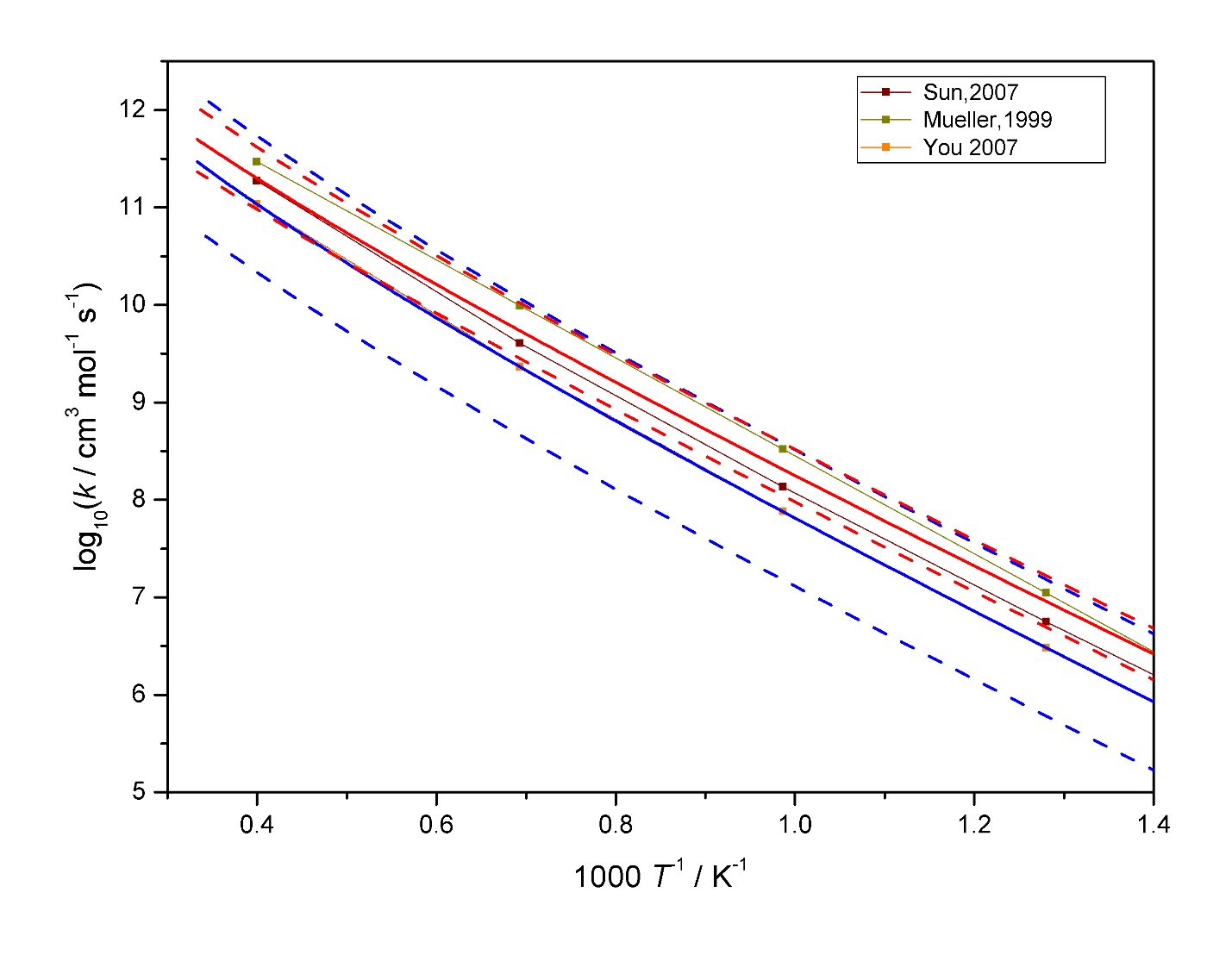


**Figure S16** Arrhenius plot of optimized rate coefficient of reaction CO + HȮ_2_ = CO_2_ + ȮH (R25). Additional results from [[51](#_ENREF_51), [80](#_ENREF_80), [185](#_ENREF_185)] are also plotted.


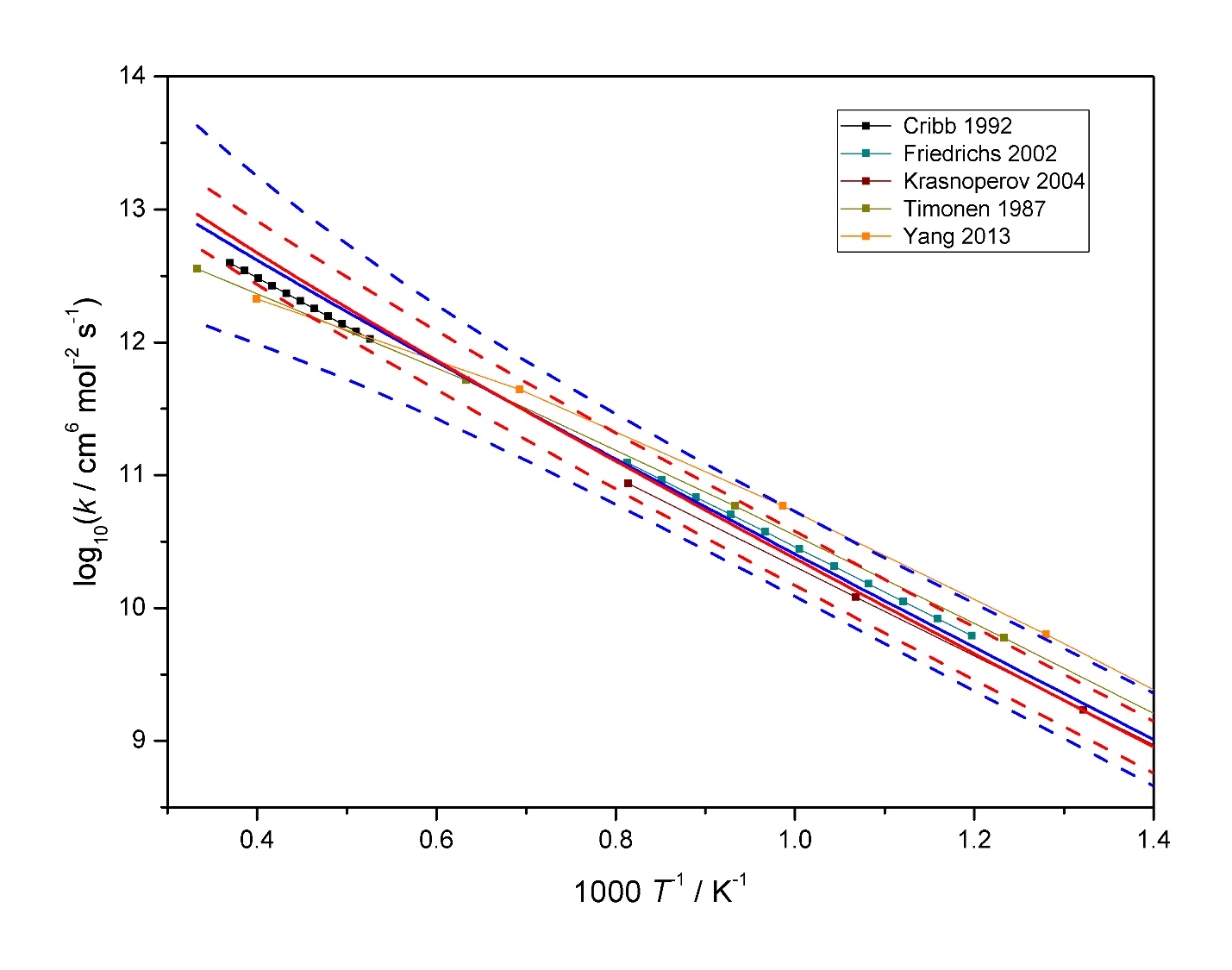


**Figure S17** Arrhenius plot of optimized low-pressure limiting rate coefficient of reaction HĊO + M = Ḣ + CO + M (R26). Additional results from [[142](#_ENREF_142), [186-189](#_ENREF_186)] are also plotted.


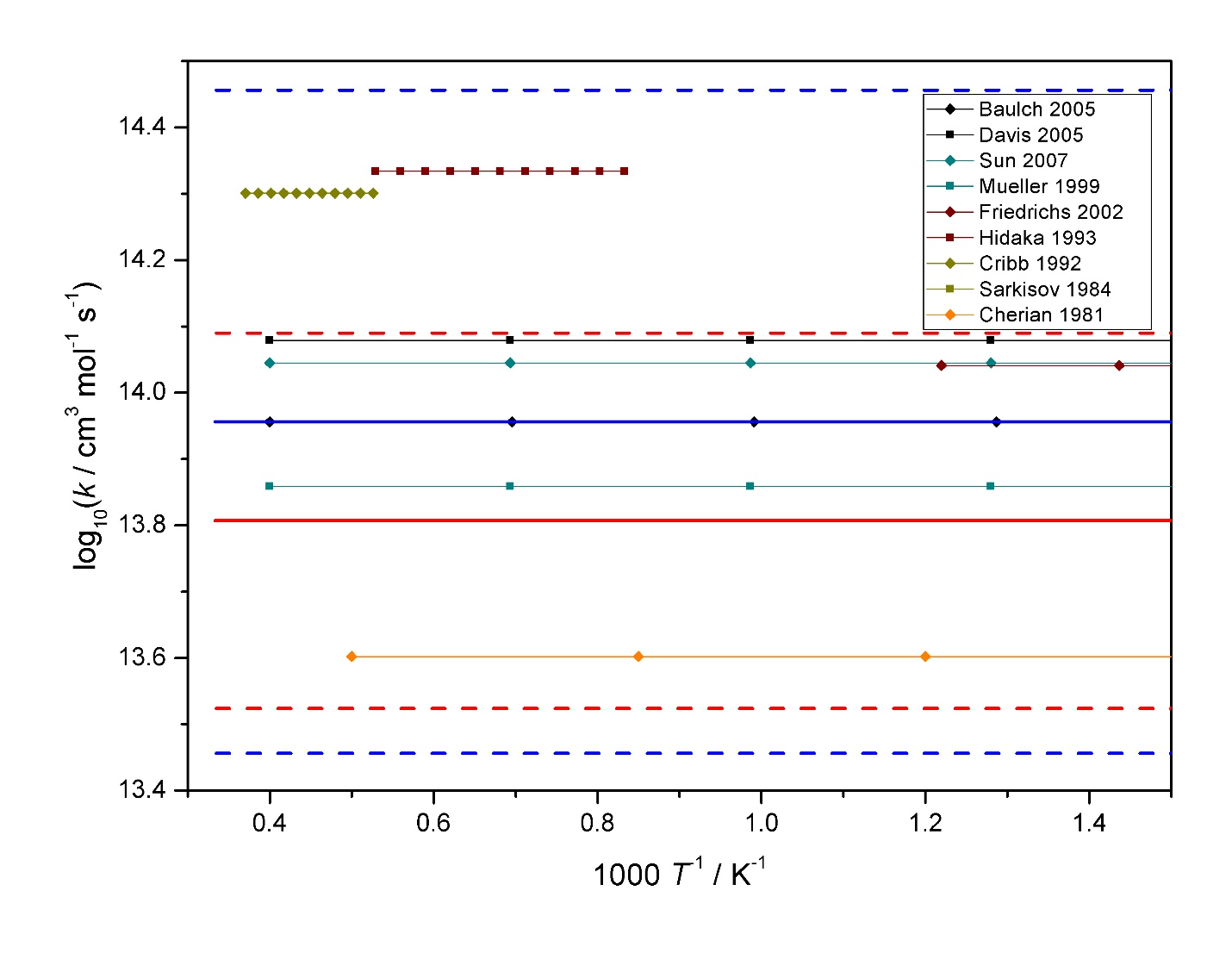


**Figure S18** Arrhenius plot of optimized rate coefficient of reaction HĊO + Ḣ = CO + H_2_ (R28). Additional results from [[51](#_ENREF_51), [68](#_ENREF_68), [80](#_ENREF_80), [158](#_ENREF_158), [179](#_ENREF_179), [186](#_ENREF_186), [187](#_ENREF_187), [190](#_ENREF_190), [191](#_ENREF_191)] are also plotted.

# Temperature dependent uncertainties of the optimized rate coefficients

**Figure S19** Temperature dependent uncertainty values for the optimized rate coefficients


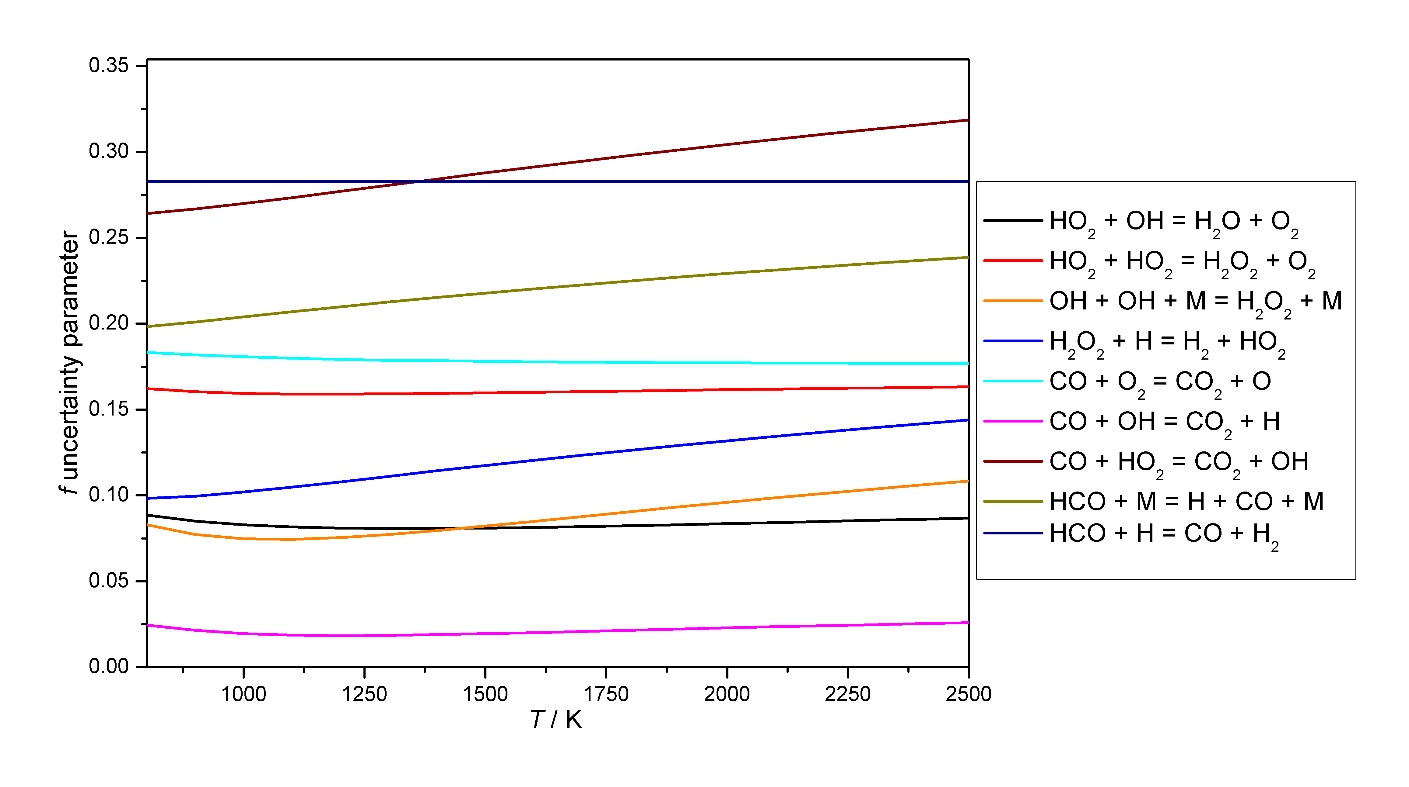


**Figure S20** Temperature dependent uncertainty values for the optimized rate coefficients


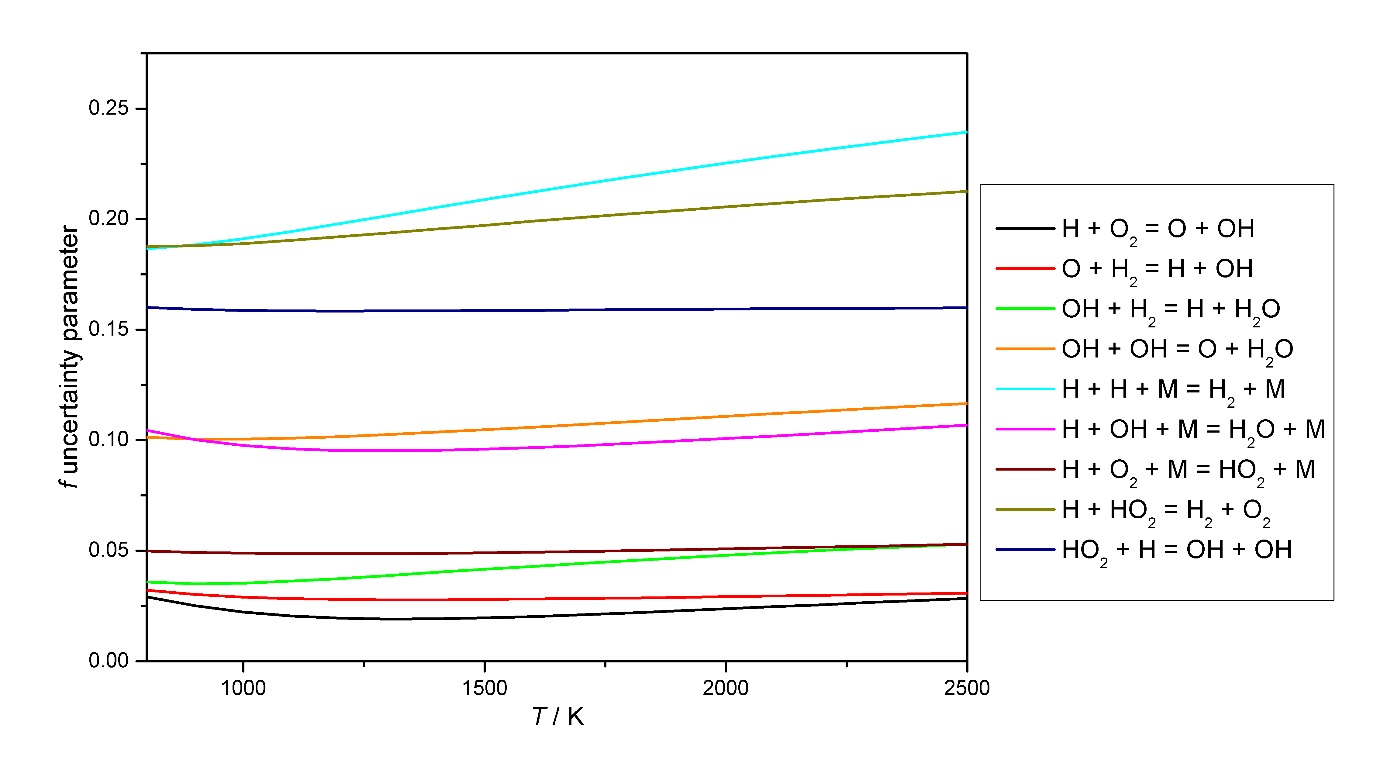


# Residuals for spline fits of burning velocity datasets

As described in Section 5 of the manuscript, the standard deviation of each dataset was determined based on fitting a polynomial or spline function to the data, and the residual of the fit was used to calculate the standard deviation. Figures S21 and S22 show such residuals for laminar burning velocity measurements, as a function of the measured burning velocities. The figures demonstrate that the residuals do not have an obvious dependence on the measured burning velocities, and the selection of constant absolute errors is justified.

The assigned absolute standard deviation levels are given with dashed lines, and the relative standard deviation levels that could be calculated from the same residuals are given with dotted lines. Note that the indicated standard deviations belong to the plotted residuals. The standard deviations assigned to the datasets were also scaled considering the degrees of freedom of the polynomial/spline fitting. If the obtained standard deviation was lower than 2 cm/s, then this threshold value was assigned. Therefore, the standard deviations indicated in the figures do not correspond directly to those given in Tables S1–S14.

**Figure S21** Residuals, their absolute standard deviation (dashed line), and their relative standard deviation (dotted line) for the laminar burning velocity dataset of Tse *et al.* [[28](#_ENREF_28)].


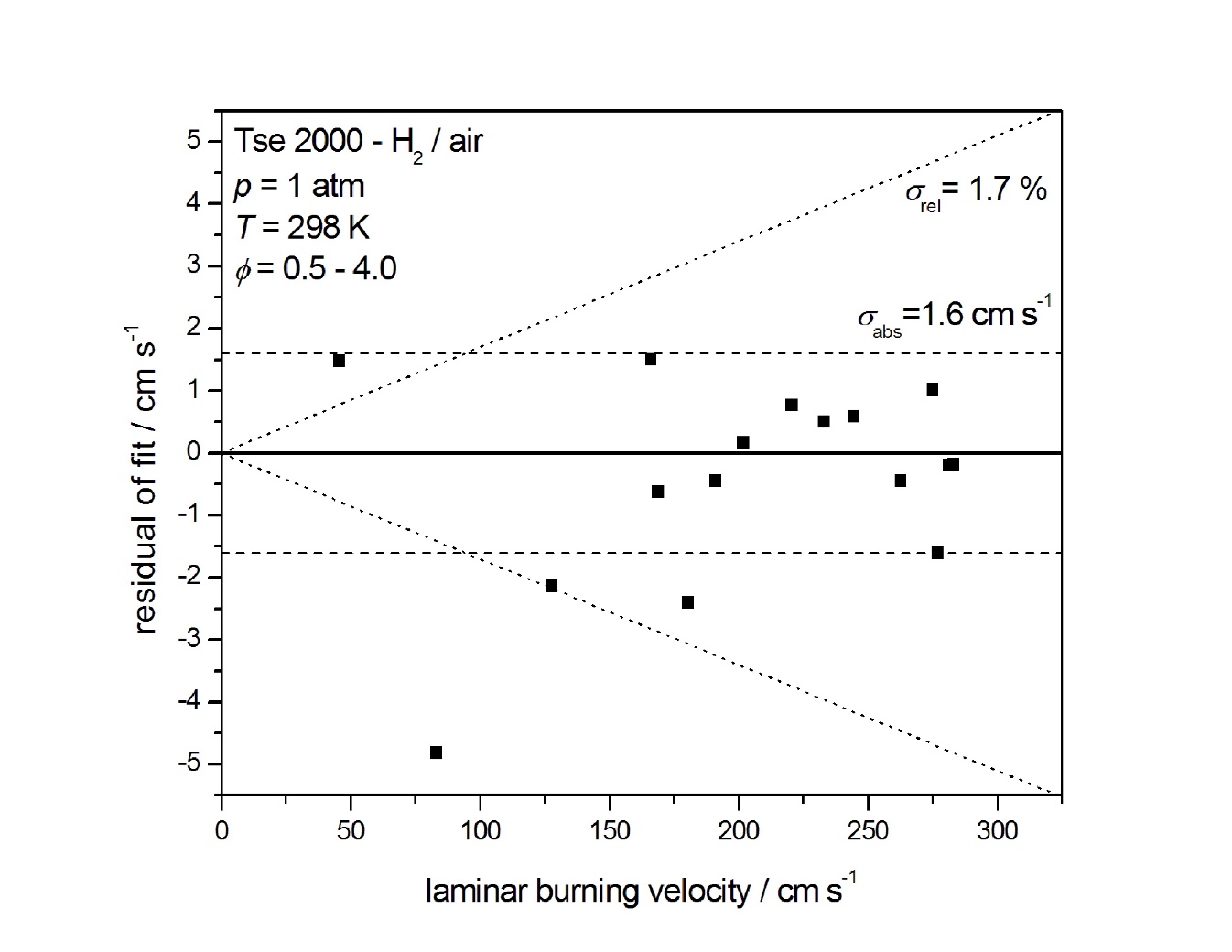


**Figure S22** Residuals, their absolute standard deviation (dashed line), and their relative standard deviation (dotted line) for the laminar burning velocity dataset of Bradley *et al.* [[24](#_ENREF_24)].


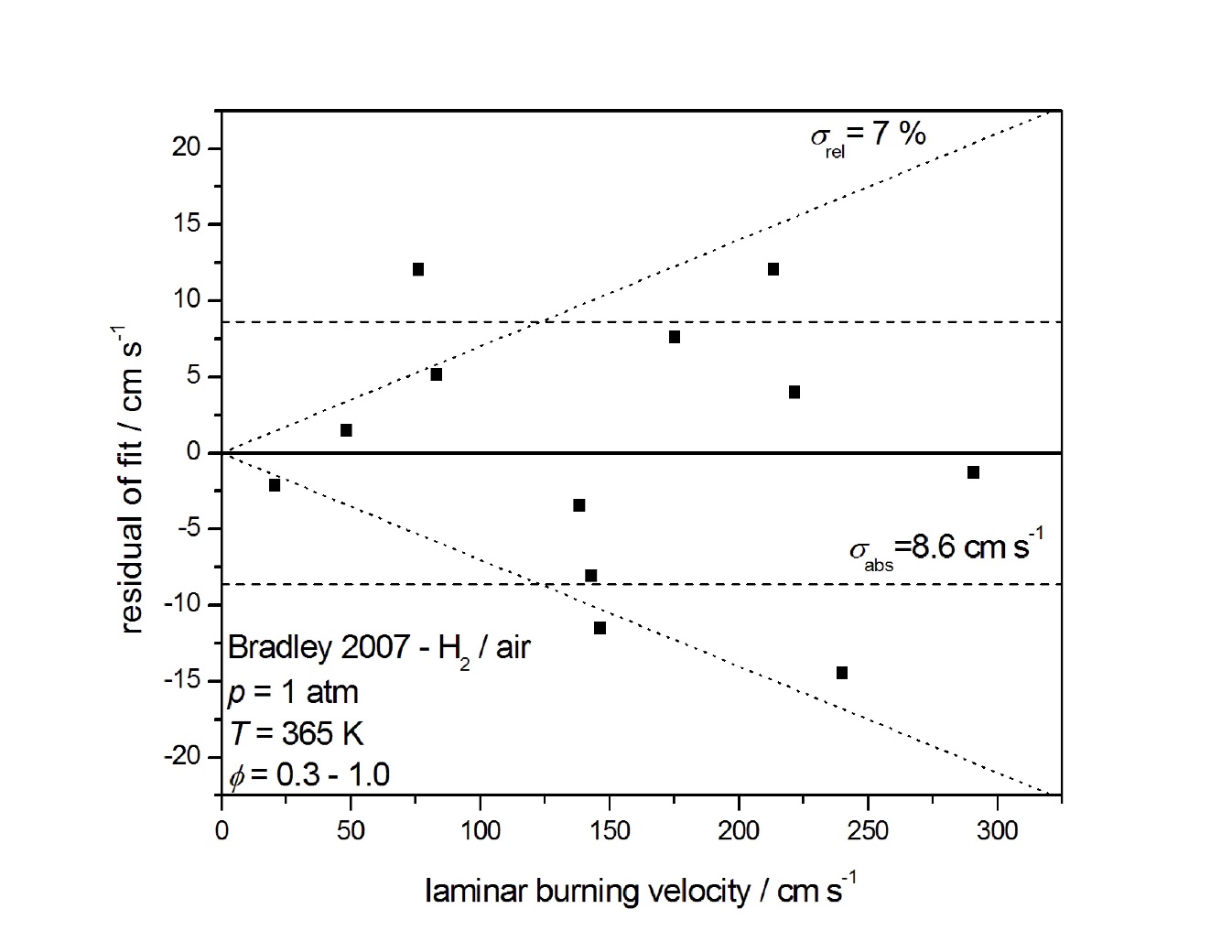


# References

1. C. Olm; I. G. Zsély; R. Pálvölgyi; T. Varga; T. Nagy; H. J. Curran; T. Turányi, Combust. Flame 2014, 161, 2219–2234.

2. C. Olm; I. G. Zsély; T. Varga; H. J. Curran; T. Turányi, Combust. Flame 2015, 162, 1793–1812.

3. G. A. Pang; D. F. Davidson; R. K. Hanson, Proc. Combust. Inst. 2009, 32, 181–188.

4. J. Herzler; C. Naumann, Proc. Combust. Inst. 2009, 32, 213–220.

5. E. L. Petersen; D. F. Davidson; M. Röhrig; R. K. Hanson, Shock Waves 1996, 941–946.

6. R. K. Cheng; A. K. Oppenheim, Combust. Flame 1984, 58, 125–139.

7. A. Cohen; J. Larsen, Technical Report BRL 1386 (Ballistics Research Laboratories) 1967,

8. G. B. Skinner; G. H. Ringrose, J. Chem. Phys. 1965, 42, 2190–2192.

9. G. L. Schott; J. L. Kinsey, J. Chem. Phys. 1958, 29, 1177–1182.

10. M. W. Slack, Combust. Flame 1977, 28, 241–249.

11. E. L. Petersen; D. M. Kalitan; M. J. A. Rickard in: Chemical Kinetics of OH* Chemiluminescence in High-Temperature Reacting Flows Proceedings of the Third Joint Meeting of the U.S. Sections of The Combustion Institute, Chicago, IL, 2003; Chicago, IL.

12. K. A. Bhaskaran; M. C. Gupta; T. H. Just, Combust. Flame 1973, 21, 45–48.

13. B. L. Wang; H. Olivier; H. Gronig, Combust. Flame 2003, 133, 93–106.

14. T. Asaba; W. C. Gardiner; R. F. Stubbelman, Proc. Combust. Inst. 1965, 10, 295–302.

15. Y. Zhang; Z. Huang; L. Wei; J. Zhang; C. K. Law, Combust. Flame 2012, 159, 918–931.

16. C. Naumann; J. Herzler; P. Griebel; H. J. Curran; A. Kéromnès; I. Mantzaras, in: 7^th^ Framework Programme FP7-ENERGY-2008-TREN-1 ENERGY-2008-6-CLEAN COAL TECHNOLOGIES, Deliverable 1.1.3, 2011.

17. A. Kéromnès; W. K. Metcalfe; K. A. Heufer; N. Donohoe; A. K. Das; C.-J. Sung; J. Herzler; C. Naumann; P. Griebel; O. Mathieu; M. C. Krejci; E. L. Petersen; W. J. Pitz; H. J. Curran, Combust. Flame 2013, 160, 995–1011.

18. A. K. Das; C.-J. Sung; Y. Zhang; G. Mittal, Int. J. Hydrogen Energy 2012, 37, 6901–6911.

19. G. Mittal; C. J. Sung; R. A. Yetter, Int. J. Chem. Kinet. 2006, 38, 516–529.

20. M. A. Mueller; T. J. Kim; R. A. Yetter; F. L. Dryer, Int. J. Chem. Kinet. 1999, 31, 113–125.

21. G. T. Linteris; R. A. Yetter; K. Brezinsky; F. L. Dryer, Combust. Flame 1991, 86, 162–170.

22. T. Le Cong; P. Dagaut, Energ. Fuel 2009, 23, 725–734.

23. G. W. Koroll; R. K. Kumar; E. M. Bowles, Combust. Flame 1993, 94, 330–340.

24. D. Bradley; M. Lawes; K. Liu; S. Verhelst; R. Woolley, Combust. Flame 2007, 149, 162–172.

25. S. C. Taylor. Burning Velocity and the Influence of Flame Stretch. University of Leeds, 1991.

26. F. Takahashi; M. Mizomoto; S. Ikai, Alternative Energy Sources III 1983, 5, 447–457.

27. T. IIjima; T. Takeno, Combust. Flame 1986, 65, 35–43.

28. S. D. Tse; D. L. Zhu; C. K. Law, Proc. Combust. Inst. 2000, 28, 1793–1800.

29. C. M. Vagelopoulos; F. N. Egolfopoulos; C. K. Law, Proc. Combust. Inst. 1994, 25, 1341–1347.

30. F. N. Egolfopoulos; C. K. Law, Proc. Combust. Inst. 1990, 23, 333–340.

31. R. T. E. Hermanns; A. A. Konnov; R. J. M. Bastiaans; L. P. H. de Goey, Energ. Fuel 2007, 21, 1977–1981.

32. N. Lamoureux; N. Djebaili-Chaumeix; C. E. Paillard, Exp. Therm. Fluid Sci. 2003, 27, 385–393.

33. K. T. Aung; M. I. Hassan; G. M. Faeth, Combust. Flame 1998, 112, 1–15.

34. O. C. Kwon; G. M. Faeth, Combust. Flame 2001, 124, 590–610.

35. M. P. Burke; M. Chaos; F. L. Dryer; Y. Ju, Combust. Flame 2010, 157, 618–631.

36. W. Tang; K. Brezinsky, Int. J. Chem. Kinet. 2006, 38, 75–97.

37. Z. Huang; Y. Zhang; K. Zeng; B. Liu; Q. Wang; D. Jiang, Combust. Flame 2006, 146, 302–311.

38. M. P. Burke; F. L. Dryer; Y. Ju, Proc. Combust. Inst. 2011, 33, 905–912.

39. L. Qiao; Y. Gu; W. J. A. Dahm; E. S. Oran; G. M. Faeth, Combust. Flame 2007, 151, 196–208.

40. C. Dong; Q. Zhou; Q. Zhao; Y. Zhang; T. Xu; S. Hui, Fuel 2009, 88, 1858–1863.

41. J. Santner; F. L. Dryer; Y. Ju, Proc. Combust. Inst. 2013, 34, 719–726.

42. H. Hashemi; J. M. Christensen; S. Gersen; P. Glarborg, Proc. Combust. Inst. 2015, 35, 553–560.

43. E. L. Petersen; D. M. Kalitan; A. B. Barrett; S. C. Reehal; J. D. Mertens; D. J. Beerer; R. L. Hack; V. G. McDonell, Combust. Flame 2007, 149, 244–247.

44. D. M. Kalitan; J. D. Mertens; M. W. Crofton; E. L. Petersen, J. Propul. Power 2007, 23, 1291–1303.

45. A. M. Dean; R. L. Johnson, Combust. Flame 1980, 37, 109–123.

46. J. Herzler; C. Naumann, Combust. Sci. Technol. 2008, 180, 2015–2028.

47. M. C. Krejci; O. Mathieu; A. J. Vissotski; S. Ravi; T. G. Sikes; E. L. Petersen; A. Kéromnès; W. K. Metcalfe; H. J. Curran, Proceedings of the ASME Turbo Expo, June 11-15, 2012, Copenhagen, Denmark, Paper GT2012–69290

48. L. D. Thi; Y. Zhang; J. Fu; Z. Huang; Y. Zhang, Can. J. Chem. Eng. 2014, 92, 861–870.

49. A. B. Mansfield; M. S. Wooldridge, Combust. Flame 2014, 161, 2242–2251.

50. A. K. Das; K. Kumar; C.-J. Sung, Combust. Flame 2011, 158, 345–353.

51. H. Sun; S. I. Yang; G. Jomaas; C. K. Law, Proc. Combust. Inst. 2007, 31, 439–446.

52. J. Natarajan; Y. Kochar; T. Lieuwen; J. Seitzman, Proc. Combust. Inst. 2009, 32, 1261–1268.

53. C. Prathap; A. Ray; M. R. Ravi, Combust. Flame 2008, 155, 145–160.

54. N. Bouvet; S. Y. Lee; I. Gökalp; R. J. Santoro, Proc. Europ. Combust. Meeting 2007, 3, 6–17.

55. I. C. McLean; D. B. Smith; S. C. Taylor, Proc. Combust. Inst. 1994, 25, 749–757.

56. H. J. Burbano; J. Pareja; A. A. Amell, Int. J. Hydrogen Energy 2011, 36, 3232–3242.

57. J. Natarajan; T. Lieuwen; J. Seitzman, Combust. Flame 2007, 151, 104–119.

58. N. Bouvet; C. Chauveau; I. Gökalp; F. Halter, Proc. Combust. Inst. 2011, 33, 913–920.

59. M. I. Hassan; K. T. Aung; G. M. Faeth, J. Propul. Power 1997, 13, 239–245.

60. V. Ratna Kishore; M. R. Ravi; A. Ray, Combust. Flame 2011, 158, 2149–2164.

61. A. A. Konnov; I. V. Dyakov; J. De Ruyck, Proc. Combust. Inst. 2002, 29, 2171–2177.

62. Y. Huang; C. J. Sung; J. A. Eng, Combust. Flame 2004, 139, 239–251.

63. C. M. Vagelopoulos; F. N. Egolfopoulos, Proc. Combust. Inst. 1994, 25, 1317–1323.

64. G. Jahn. Der Zündvorgang in Gasgemischen (Diss.). Oldenbourg, Berlin, 1934.

65. B. Lewis; G. von Elbe, Combustion, Flames and Explosions of Gases, Academic Press, New York and London, 1961

66. D. Singh; T. Nishiie; S. Tanvir; L. Qiao, Fuel 2012, 94, 448–456.

67. T. G. Scholte; P. B. Vaags, Combust. Flame 1959, 3, 511–524.

68. M. A. Cherian; P. Rhodes; R. J. Simpson; G. Dixon-Lewis, Proc. Combust. Inst. 1981, 18, 385–396.

69. M. C. Krejci; A. J. Vissotski; S. Ravi; W. K. Metcalfe; A. Kéromnès; H. J. Curran; E. L. Petersen, Spring Technical Meeting of the Central States Section of the Combustion Institute, April 22–24, 2012, Dayton, OH

70. X. Zhang; Z. Huang; Z. Zhang; J. Zheng; W. Yu; D. Jiang, Int. J. Hydrogen Energy 2009, 34, 4862–4875.

71. M. P. Burke; X. Qin; Y. Ju; F. L. Dryer, in: 5^th^ US Combustion Meeting Organized by the Western States Section of the Combustion Institute, University of California at San Diego, March 25–28, 2007, Paper #A16.

72. B. Lohöfener; E. Roungos; S. Voss; D. Trimis, in: 2nd Heat Flux Burner Workshop, Warsaw University of Technology, Poland, July 29, 2012.

73. W. B. Weng; W. Wang; Y. He; Y. J. Zhou; J. H. Zhou; K. F. Cen, Proc. Europ. Combust. Meeting 2013, 6, P3–4.

74. M. Goswami; R. J. M. Bastiaans; A. A. Konnov; L. P. H. de Goey, Int. J. Hydrogen Energy 2014, 39, 1485–1498.

75. J. S. Kim; J. Park; D. S. Bae; T. M. Vu; J. S. Ha; T. K. Kim, Int. J. Hydrogen Energy 2010, 35, 1390–1400.

76. F. Wu; A. P. Kelley; C. Tang; D. Zhu; C. K. Law, Int. J. Hydrogen Energy 2011, 36, 13171–13180.

77. S. Voss; S. Hartl; C. Hasse, Int. J. Hydrogen Energy 2014, accepted.

78. Y. Zhang; W. Shen; M. Fan; H. Zhang; S. Li, Combust. Flame 2014, 161, 2492–2495.

79. X. Li; X. You; F. Wu; C. K. Law, Proc. Combust. Inst. 2015, 35, 617–624.

80. M. A. Mueller; R. A. Yetter; F. L. Dryer, Int. J. Chem. Kinet. 1999, 31, 705–724.

81. T. J. Kim; R. A. Yetter; F. L. Dryer, in: Proc. Combust. Inst., 1994; Vol. 25, pp 759–766.

82. J. Li; Z. Zhao; A. Kazakov; M. Chaos; F. L. Dryer; J. J. J. Scire, Int. J. Chem. Kinet. 2007, 39, 109–136.

83. P. Glarborg; D. Kubel; K. Dam-Johansen; H.-M. Chiang; J. W. Bozzelli, Int. J. Chem. Kinet. 1996, 28, 773–790.

84. M. U. Alzueta; R. Bilbao; P. Glarborg, Combust. Flame 2001, 127, 2234–2251.

85. R. Sivaramakrishnan; A. Comandini; R. S. Tranter; K. Brezinsky; S. G. Davis; H. Wang, Proc. Combust. Inst. 2007, 31, 429–437.

86. P. Dagaut; F. Lecomte; J. Mieritz; P. Glarborg, Int. J. Chem. Kinet. 2003, 35, 564–575.

87. D. A. Masten; R. K. Hanson; C. T. Bowman, J. Phys. Chem. 1990, 94, 7119–7128.

88. H. Du; J. P. Hessler, J. Chem. Phys. 1992, 96, 1077–1092.

89. H. Yang; W. C. Gardiner; K. S. Shin; N. Fujii, Chem. Phys. Lett. 1994, 231, 449–453.

90. S. O. Ryu; S. M. Hwang; M. J. Rabinowitz, J. Phys. Chem. 1995, 99, 13984–13991.

91. A. N. Pirraglia; J. V. Michael; J. W. Sutherland; R. B. Klemm, J. Phys. Chem. 1989, 93, 282–291.

92. K. S. Shin; J. V. Michael, J. Chem. Phys. 1991, 95, 262–273.

93. S. M. Hwang; S. O. Ryu; K. J. De Witt; M. J. Rabinowitz, Chem. Phys. Lett. 2005, 408, 107–111.

94. Z. Hong; D. F. Davidson; E. A. Barbour; R. K. Hanson, Proc. Combust. Inst. 2011, 33, 309–316.

95. S. O. Ryu; S. M. Hwang; M. J. Rabinowitz, Chem. Phys. Lett. 1995, 242, 279–284.

96. D. F. Davidson; R. K. Hanson, Combust. Flame 1990, 82, 445–447.

97. N. Presser; R. J. Gordon, J. Chem. Phys. 1985, 82, 1291–1297.

98. G. C. Light; J. H. Matsumoto, Int. J. Chem. Kinet. 1980, 7, 451–468.

99. S. Javoy; V. Naudet; S. Abid; C. E. Paillard, Int. J. Chem. Kinet. 2000, 32, 686-695.

100. K. Natarajan; P. Roth, Combust. Flame 1987, 70, 267-279.

101. J. W. Sutherland; J. V. Michael; A. N. Pirraglia; F. L. Nesbitt; R. B. Klemm, Proc. Combust. Inst. 1986, 21, 929–941.

102. H. X. Yang; K. S. Shin; W. Gardiner, Chem. Phys. Lett. 1993, 207, 69-74.

103. J. V. Michael; J. W. Sutherland, J. Phys. Chem. 1988, 92, 3853–3857.

104. R. C. Oldenborg; G. W. Loge; D. M. Harradine; K. R. Winn, J. Chem. Phys. 1992, 96, 8426–8430.

105. D. F. Davidson; A. Y. Chang; R. K. Hanson, Proc. Combust. Inst. 1988, 22, 1877-1885.

106. P. Frank; T. H. Just, Ber. Bunsenges. Phys. Chem. 1985, 89, 181–187.

107. A. R. Ravishankara; J. M. Nicovich; R. L. Thompson; F. P. Tully, J. Phys. Chem. 1981, 85, 2498–2503.

108. F. P. Tully; A. R. Ravishankara, J. Phys. Chem. 1980, 84, 3126–3130.

109. K. Y. Lam; D. F. Davidson; R. K. Hanson, Int. J. Chem. Kinet. 2013, 45, 363-373.

110. J. W. Sutherland; P. M. Patterson; R. B. Klemm, Proc. Combust. Inst. 1990, 23, 51–57.

111. A. Lifshitz; J. V. Michael, Proc. Combust. Inst. 1991, 23, 59–67.

112. G. Wagner; R. Zellner, Ber. Bunsenges. Phys. Chem. 1981, 85, 1122–1128.

113. W. C. Gardiner Jr; W. G. Mallard; M. McFarland; K. Morinaga; J. H. Owen; W. T. Rawlins; T. Takeyama; B. F. Walker, Proc. Combust. Inst. 1973, 14, 61–75.

114. A. Gay; N. H. Pratt, Proc. Int. Symp. Shock Tubes Waves 1971, 8,

115. C. J. Halstead; D. R. Jenkins, Proc. Combust. Inst. 1969, 12, 979–987.

116. M. A. Mueller; R. A. Yetter; F. L. Dryer, in: Proc. Combust. Inst., 1998; Vol. 27, pp 177–184.

117. P. J. Ashman; B. S. Haynes, in: Proc. Combust. Inst., 1998; Vol. 27, pp 185–191.

118. R. W. Getzinger; G. L. Schott, J. Chem. Phys. 1965, 43, 3237–3247.

119. R. W. Getzinger; L. S. Blair, Combust. Flame 1969, 13, 271–284.

120. L. S. Blair; R. W. Getzinger, Combust. Flame 1970, 14, 5–12.

121. J. V. Michael; M.-C. Su; J. W. Sutherland; J. J. Carroll; A. F. Wagner, J. Phys. Chem. A 2002, 106, 5297–5313.

122. J. V. Michael; J. W. Sutherland; L. B. Harding; A. F. Wagner, in: Proc. Combust. Inst., 2000; Vol. 28, pp 1471–1478.

123. Z. Hong; S. S. Vasu; D. F. Davidson; R. K. Hanson, J. Phys. Chem. A 2010, 114, 5520-5525.

124. Z. Hong; K.-Y. Lam; R. Sur; S. Wang; D. F. Davidson; R. K. Hanson, Proc. Combust. Inst. 2013, 34, 565–571.

125. N. K. Srinivasan; M.-C. Su; J. W. Sutherland; J. V. Michael; B. Ruscic, J. Phys. Chem. A 2006, 110, 6602–6607.

126. H. Hippler; J. Troe; J. Willner, J. Chem. Phys. 1990, 93, 1755–1760.

127. C. Kappel; K. Luther; J. Troe, Phys. Chem. Chem. Phys. 2002, 4, 4392–4398.

128. Z. Hong; A. Farooq; E. A. Barbour; D. F. Davidson; R. K. Hanson, J. Phys. Chem. A 2009, 113, 12919–12925.

129. Z. Hong; R. D. Cook; D. F. Davidson; R. K. Hanson, J. Phys. Chem. A 2010, 114, 5718–5727.

130. Z. Hong; D. F. Davidson; K.-Y. Lam; R. K. Hanson, Combust. Flame 2012, 159, 3007–3013.

131. K. Thielen; P. Roth, Ber. Bunsenges. Phys. Chem. 1983, 87, 920–925.

132. M. S. Wooldridge; R. K. Hanson; C. T. Bowman, Proc. Combust. Inst. 1994, 25, 741–748.

133. A. A. Westenberg; N. deHaas, J. Chem. Phys. 1973, 58, 4061–4065.

134. A. R. Ravishankara; R. L. Thompson, Chem. Phys. Lett. 1983, 99, 377–381.

135. T. A. Brabbs; F. E. Belles; R. S. Brokaw, Proc. Combust. Inst. 1970, 13, 129–136.

136. D. M. Golden; G. P. Smith; A. B. McEwen; C. L. Yu; B. Eiteneer; M. Frenklach; G. L. Vaghjiani; A. R. Ravishankara; F. P. Tully, J. Phys. Chem. A 1998, 102, 8598–8606.

137. M. S. Wooldridge; R. K. Hanson; C. T. Bowman, Int. J. Chem. Kinet. 1996, 28, 361–372.

138. J. Vandooren; J. Peeters; P. J. Van Tiggelen, Proc. Combust. Inst. 1975, 15, 745–753.

139. J. Peeters; G. Mahnen, Proc. Combust. Inst. 1973, 14, 133–146.

140. K. H. Eberius; K. Hoyermann; H. G. Wagner, Proc. Combust. Inst. 1973, 14, 147–156.

141. H. Hippler; N. Krasteva; F. Striebel, Phys. Chem. Chem. Phys. 2004, 6, 3383–3388.

142. L. N. Krasnoperov; E. N. Chesnokov; H. Stark; A. R. Ravishankara, J. Phys. Chem. A 2004, 108, 11526–11536.

143. T. Yuan; C. Wang; C.-L. Yu; M. Frenklach; M. J. Rabinowitz, J. Phys. Chem. 1991, 95, 1258–1265.

144. N. Fujii; K. S. Shin, Chem. Phys. Lett. 1988, 151, 461-465.

145. C.-L. Yu; M. Frenklach; D. A. Masten; R. K. Hanson; C. T. Bowman, J. Phys. Chem. 1994, 98, 4770–4771.

146. J. A. Miller; B. C. Garrett, Int. J. Chem. Kinet. 1997, 29, 275-287.

147. T. C. Germann; W. H. Miller, J. Phys. Chem. A 1997, 101, 6358-6367.

148. S. Javoy; V. Naudet; S. Abid; C. E. Paillard, Exp. Therm. Fluid Sci. 2003, 27, 371–377.

149. K. S. Shin; N. Fujii; W. C. J. Gardiner, Chem. Phys. Lett. 1989, 161, 219-222.

150. J. W. Sutherland; R. B. Klemm, Proc. Combust. Inst. 1987, 16,

151. P. Marshall; A. Fontijn, J. Chem. Phys. 1987, 87, 6988-6994.

152. P. Roth; T. Just, Proc. Combust. Inst. 1985, 20, 807-818.

153. N. L. Haworth; G. B. Bacskay; J. C. Mackie, J. Phys. Chem. A 2002, 106, 1533-1541.

154. M. J. Cohen; A. Willetts; N. C. Handy, J. Chem. Phys. 1993, 99, 5885-5897.

155. M. S. Wooldridge; R. K. Hanson; C. T. Bowman, Int. J. Chem. Kinet. 1994, 26, 389-401.

156. J. W. Sutherland; P. M. Patterson; R. B. Klemm, Proc. Combust. Inst. 1991, 23, 51-57.

157. L. B. Harding; A. F. Wagner, Proc. Combust. Inst. 1989, 22, 983-989.

158. D. L. Baulch; C. T. Bowman; C. J. Cobos; R. A. Cox; T. Just; J. A. Kerr; M. J. Pilling; D. Stocker; J. Troe; W. Tsang; R. W. Walker; J. Warnatz, J. Phys. Chem. Ref. Data 2005, 34, 757-1397.

159. A. A. Konnov, Combust. Flame 2008, 152, 507-528.

160. M. P. Burke; M. Chaos; Y. Ju; F. L. Dryer; S. J. Klippenstein, Int. J. Chem. Kinet. 2012, 44, 444-474.

161. M. Ó Conaire; H. J. Curran; J. M. Simmie; W. J. Pitz; C. K. Westbrook, Int. J. Chem. Kinet. 2004, 36, 603-622.

162. Z. Hong; D. F. Davidson; R. K. Hanson, Combust. Flame 2011, 158, 633-644.

163. W. G. Mallard; J. H. Owen, Int. J. Chem. Kinet. 1974, 6, 753-761.

164. J. M. Goodings; A. N. Hayhurst, Journal of Chemical Society, Faraday Transactions 2 1988, 84, 745-762.

165. D. F. Davidson; E. L. Petersen; M. Rohrig; R. K. Hanson; C. T. Bowman, Proc. Combust. Inst. 1996, 26, 481-488.

166. P. J. Ashman; B. S. Haynes, Proc. Combust. Inst. 1998, 27, 185-191.

167. R. W. Bates; D. M. Golden; R. K. Hanson; C. T. Bowman, PCCP 2001, 3, 2337-2342.

168. N. M. Marinov; C. K. Westbrook; W. J. Pitz, Proceedings of the Eighth International Symposium on Transport Phenomena in Combustion 1996, 1, 118-129.

169. J. Troe, Combust. Flame 2011, 158, 594-601.

170. R. C. Brown; C. E. Kolb; R. A. Yetter; F. L. Dryer; H. Rabitz, Combust. Flame 1995, 101, 221–238.

171. S. P. Karach; V. I. Osherov, J. Chem. Phys. 1999, 110, 11918-11927.

172. R. Shaw, Int. J. Chem. Kinet. 1977, 9, 929-941.

173. S. H. Mousavipour; V. Saheb, Bulletin of the Chemical Society of Japan 2007, 80, 1901-1913.

174. H. Hippler; J. Troe, Chem. Phys. Lett. 1992, 192, 333.

175. C. Gonzalez; J. Theisen; H. B. Schlegel; W. L. Hase; E. W. Kaiser, J. Phys. Chem. 1992, 96, 1767-1774.

176. D. Lee; S. Hochgreb, Int. J. Chem. Kinet. 1998, 30, 385–406.

177. T. Koike, Bulletin of the Chemical Society of Japan 1991, 64, 1726-1730.

178. W. T. Rawlins; W. C. J. Gardiner, J. Phys. Chem. 1974, 7, 497-500.

179. S. G. Davis; A. V. Joshi; H. Wang; F. Egolfopoulos, Proc. Combust. Inst. 2005, 30, 1283–1292.

180. V. Lissianski; H. Yang; Z. Qin; M. R. Mueller; K. S. Shin; W. C. Gardiner, Chem. Phys. Lett. 1995, 240, 57-62.

181. C. W. Larson; P. H. Stewart; D. M. Golden, Int. J. Chem. Kinet. 1988, 20, 27-40.

182. A. Wawer; M. Zielski, Nukleonika 1978, 23, 903-910.

183. M. Mozurkewich; J. J. Lamb; S. W. Benson, J. Phys. Chem. 1984, 88, 6435-6441.

184. R. E. Weston; T. L. Nguyen; J. F. Stanton; J. R. Barker, J. Phys. Chem. A 2013, 117, 821-835.

185. X. Q. You; H. Wang; E. Goos; C. J. Sung; S. J. Klippenstein, J. Phys. Chem. A 2007, 111, 4031-4042.

186. P. H. Cribb; J. E. Dove; S. Yamazaki, Combust. Flame 1992, 88, 169-185.

187. G. Friedrichs; J. T. Herbon; D. F. Davidson; R. K. Hanson, Phys. Chem. Chem. Phys. 2002, 4, 5778 - 5788.

188. R. S. Timonen; E. Ratajczak; D. Gutman; A. F. Wagner, J. Phys. Chem. 1987, 91, 5325-5332.

189. X. Yang; T. Tan; P. Diévart; E. A. Carter; Y. Ju in: Theoretical assessment on reaction kinetics HCO and CH2OH unimolecular decomposition, 8th U. S. National Combustion Meeting, 2013.

190. Y. Hidaka; T. Taniguchi; T. Kamesawa; H. Masaoka; K. Inami; H. Kawano, Int. J. Chem. Kinet. 1993, 25, 305-322.

191. O. M. Sarkisov; S. G. Cheskis; V. A. Nadtochenko; E. A. Sviridenkov; V. I. Vedeneev, Archivum Combustionis 1984, 4,
